# The changing mouse embryo transcriptome at whole tissue and single-cell resolution

**DOI:** 10.1038/s41586-020-2536-x

**Published:** 2020-07-29

**Authors:** Peng He, Brian A. Williams, Diane Trout, Georgi K. Marinov, Henry Amrhein, Libera Berghella, Say-Tar Goh, Ingrid Plajzer-Frick, Veena Afzal, Len A. Pennacchio, Diane E. Dickel, Axel Visel, Bing Ren, Ross C. Hardison, Yu Zhang, Barbara J. Wold

**Affiliations:** 1 Division of Biology and Biological Engineering, California Institute of Technology, Pasadena, CA USA; 2 Department of Genetics, Stanford University, Palo Alto, CA USA; 3 Environmental Genomics and Systems Biology Division, Lawrence Berkeley National Laboratory, Berkeley, CA USA; 4 Department of Energy Joint Genome Institute, Lawrence Berkeley National Laboratory, Berkeley, CA USA; 5 Comparative Biochemistry Program, University of California, Berkeley, Berkeley, CA USA; 6 School of Natural Sciences, University of California, Merced, Merced, CA USA; 7 Department of Cellular and Molecular Medicine, University of California, San Diego, La Jolla, CA USA; 8 Department of Biochemistry and Molecular Biology, Pennsylvania State University, University Park, PA USA; 9 Department of Statistics, Pennsylvania State University, University Park, PA USA; 10 Present Address: European Bioinformatics Institute (EMBL-EBI), Cambridge, UK

**Keywords:** Network topology, Developmental biology, Transcriptional regulatory elements, Transcriptomics

## Abstract

During mammalian embryogenesis, differential gene expression gradually builds the identity and complexity of each tissue and organ system^1^. Here we systematically quantified mouse polyA-RNA from day 10.5 of embryonic development to birth, sampling 17 tissues and organs. The resulting developmental transcriptome is globally structured by dynamic cytodifferentiation, body-axis and cell-proliferation gene sets that were further characterized by the transcription factor motif codes of their promoters. We decomposed the tissue-level transcriptome using single-cell RNA-seq (sequencing of RNA reverse transcribed into cDNA) and found that neurogenesis and haematopoiesis dominate at both the gene and cellular levels, jointly accounting for one-third of differential gene expression and more than 40% of identified cell types. By integrating promoter sequence motifs with companion ENCODE epigenomic profiles, we identified a prominent promoter de-repression mechanism in neuronal expression clusters that was attributable to known and novel repressors. Focusing on the developing limb, single-cell RNA data identified 25 candidate cell types that included progenitor and differentiating states with computationally inferred lineage relationships. We extracted cell-type transcription factor networks and complementary sets of candidate enhancer elements by using single-cell RNA-seq to decompose integrative *cis*-element (IDEAS) models that were derived from whole-tissue epigenome chromatin data. These ENCODE reference data, computed network components and IDEAS chromatin segmentations are companion resources to the matching epigenomic developmental matrix, and are available for researchers to further mine and integrate.

## Abstract (web summary)

RNA expression is quantified at a tissue level in seventeen mouse tissues across embryonic development, and at the single-cell level in the developing limb.

## Main

**Fig. 1:**
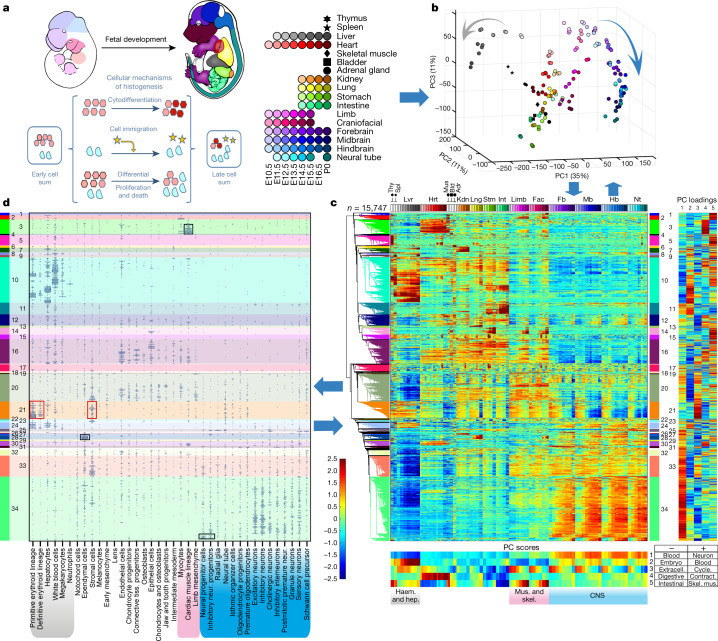
Whole-tissue polyA-RNA transcriptome structure with cell-type decomposition. **a**, Schematic of E10.5 and E15.5 embryos shows the colour key for organ identity and developmental stage across the timespan of the study with the complete key adjacent and the major cellular mechanisms of histogenesis below. **b**, Whole-tissue transcriptome top three PCs; colour code from **a** (viewable in 3D, Supplementary Video 1). *n* = 156 biological replicates. **c**, Hierarchical clustering of differentially expressed genes, heat map (bottom) for normalized log_2_(FPKM) values; two biological replicates per tissue. Thy, thymus; Spl, spleen; Lvr, liver; Hrt, heart; Mus, skeletal muscle; Bld, bladder; Adr, adrenal gland; Kdn, kidney; Lng, lung; Stm, stomach; Int, intestine; Lmb, limb; Fac, craniofacial prominence; Fb, forebrain; Mb, midbrain; Hb, hindbrain; Nt, Neural tube. Right, normalized loadings of each gene for the top five PCs. Bottom, normalized scores of the top five PCs (same sample order as clustergram). GO terms for the top 100 positive-loading and top 100 negative-loading genes abbreviated as key words (bottom right). Blood, blood microparticle; Neuron, neuron part; Embryo, embryonic morphogenesis; Extracell., extracellular region part; Cycle, mitotic cell cycle process; Digestive, digestive system process; Contract, contractile fibre part; Intestinal, intestinal epithelial cell differentiation; Skel. mus., skeletal muscle contraction. **d**, Integrating single-cell organogenesis data from whole mouse embryos^11^ with the whole-tissue transcriptome clustering (**c**). *y*-axis, genes are ordered as in **c**; *x*-axis, 38 cell types from ref. ^11^. A point in the diagram indicates expression of a marker gene from ref. ^11^ with horizontal jittering. Boxes highlight specific cell types and gene clusters of interest (see text).

**Extended Data Fig. 1:**
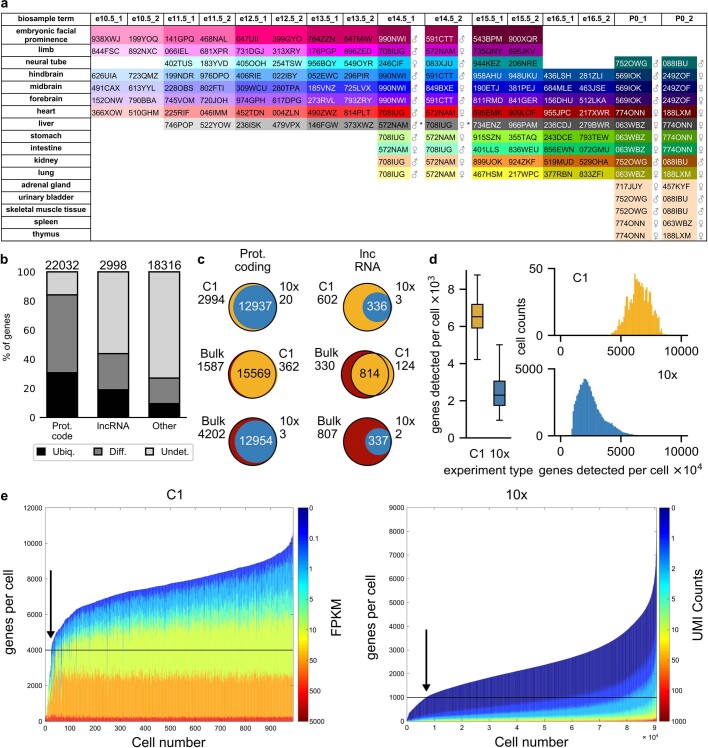
Quality metrics of bulk RNA-seq and scRNA-seq I. **a**, Table representing all bulk RNA tissue/time samples in this study according to the colour scheme in Fig. 1, including ENCODE BioSample accession numbers. The individual embryo samples for E14.5 and P0 were characterized by sex-specific expression markers; embryo sex determinations are indicated. **b**, Percentages of ubiquitous, differential and undetected genes in each of the three categories: Protein-coding genes (Prot. code), lncRNA (long intergenic noncoding RNA), and others. **c**, Pairwise comparisons of detected protein-coding genes and lncRNAs among the three RNA-seq platforms. **d**, Number of genes detected per cell by C1 and 10x platforms in box plots (left), and full histogram distributions (right panel). *n* = 920 cells for C1; *n* = 90,637 cells for 10x. **e**, Genes per cell histogram distributions coloured by abundance values. Cells are sorted in ascending order based on the number of genes detected per cell at the least stringent cutoff. Abundances are shown using the colour scale on the right of the two plots. Arrows represent the ‘knee’ cutoffs we picked for inclusion in the analysis (4,000 genes/cell for C1; 1,000 genes/cell for 10x).

Hierarchical transcription programs regulate mammalian histogenesis, a spatiotemporally coordinated process of changing cell identities, numbers and locations^1^. Contemporary RNA-seq time-courses can comprehensively quantify expression trajectories, including the transcriptional regulators that drive patterning, cell-type specification and differentiation and their regulatory targets. Here we systematically map the mouse polyadenylated RNA transcriptome, tracking 12 major tissues from embryonic day (E) 10.5 to birth (postnatal day (P) 0) (Fig. 1a, b, Extended Data Fig. 1a) to cover much of organogenesis and histogenesis. Pertinent to integrative regulatory analysis and modelling, these RNA expression data are part of the ENCODE Consortium mouse embryo project, which provides companion genome-wide microRNA, DNA methylation, histone mark, and chromatin accessibility datasets for the same sample matrix^2^. To better interpret the core sample set, we added five additional organs at P0, sampling seventeen tissues in all. As these whole-tissue data are intended for community use, including integration with high-resolution single-cell transcriptomes, we chose a widely used RNA-seq method that is robust at both bulk sample and single-cell scales^3^ and has been used for other single-cell RNA-seq (scRNA-seq) experiments in ENCODE^4^ (https://www.encodeproject.org/) and elsewhere (Tabula Muris^5^).

Single-cell RNA-seq data are increasingly used to discover and define constituent cell-types and states that comprise complex tissues such as those in our bulk mRNA-seq matrix^6–9^. For embryogenesis and regenerating systems in particular, scRNA-seq further promises to address longstanding questions about the nature and number of intermediate cell types in a developmental lineage and the regulatory mechanisms that govern transitions between them. Finally, scRNA-seq data offer an important source of input for gene network modelling by unambiguously assigning to an individual cell (or cell group) its transcription factor repertoire. Different contemporary scRNA-seq methods have complementary strengths, with some (for example, Fluidigm SMART-seq) assaying relatively modest numbers of cells with high transcript detection efficiency and RNA isoform discriminating coverage, while others (for example, 10x Genomics) capture larger cell numbers at lower transcript detection efficiency and without isoform or promoter use information^5,10–12^. We present here an ENCODE scRNA-seq resource that contains both data-types for the developing forelimb, a tissue series not represented in the Tabula Muris project^5^. We identify limb cell lineages and stages within them, and extract their corresponding cell-type marker gene sets, transcription factor (TF) networks, and promoter and distal candidate regulatory elements with their TF binding motifs. The higher sensitivity data-type additionally uncovered developmentally precocious low-level transcription of lineage-specific regulators that supports computed lineage inference models.

An emerging goal for developmental genomics is to comprehensively chart the *cis*- and *trans*-acting regulatory codes of embryogenesis with single-cell resolution. Working in this direction, we used the limb scRNA-seq data to deconvolve IDEAS enhancer element models^13,14^ that are based on whole-tissue ENCODE epigenomic data. The resulting collection of candidate active and poised enhancer elements, parsed for cell type and stage, complements matching *trans*-acting TF networks. All primary RNA-seq data and processed quantifications for tissue-level and single-cell experiments are available from the ENCODE portal (https://www.encodeproject.org).

## Results

The developmental timespan from mid-gestation (E10.5) to birth (P0) encompasses much of histogenesis and organogenesis in the mouse (Fig. 1a, Extended Data Fig. 1a). The timecourse transcriptomes clustered according to their respective tissue identities and, within tissues, by developmental time, as shown by principal component analysis (PCA) (Fig. 1b, Supplementary Data 2), *t*-distributed stochastic neighbour embedding (*t*-SNE) (Extended Data Fig. 6a), and hierarchical clustering (Fig. 1c, Extended Data Fig. 6b). Overall, this polyA-RNA transcriptome encompasses 84% of known protein coding genes and 44% of long noncoding RNA (lncRNA) genes, with the majority (15,644 genes) differing in expression level by tenfold or more across the matrix, while another 9,085 genes were more uniformly expressed (Extended Data Figs. 1b, 5a). The FANTOM5 mouse resource^10^ (https://fantom.gsc.riken.jp/5/) covers many of the same tissues and stages but is based on CAGE promoter data; we detected 97% of its 13,999 protein coding genes, plus an additional 5,035 not detected by FANTOM5 (Extended Data Fig. 3c).

## Global transcriptome structure

Neurogenesis and haematopoiesis polarize the global data structure, with transcriptomes from these systems occupying opposite ends of the first two principal components (PCs) (Fig. 1b, c). Nearly one-fifth of the expressed transcriptome (about 5,000 genes) unambiguously defines this differential axis, which was robust to the choice of quantification units (fragments per kilobase of transcript per million mapped reads (FPKM) or transcripts per million (TPM); Extended Data Fig. 6f, g) and to tissue representation (Extended Data Fig. 6d, e). Because whole-tissue data sum over all constituent cell types, their transcriptomes obscure underlying cell identities and relative cell proportions that are fundamental in histogenesis (Fig. 1a). We therefore projected cell-type marker genes and cell identities from a recent single-cell mouse whole embryo survey^11^ into our transcriptome structure (Fig. 1d). This showed that the high-complexity CNS and haematopoetic gene profiles correspond to high cellular diversity defined by the single-cell decomposition, with more than 40% of cell types mapping to CNS and haematopoetic gene clusters. Focusing in, the single-cell projection further identifies tissue-level expression of numerous gene clusters or sub-clusters that can be attributed to specific cell-type contributions (for example, ependymal cells, neural progenitor cells, or cardiomyocytes; Fig. 1d, black boxes).

## Temporal drivers

Developmental changes were expected at the tissue level, but we did not know in advance what genes and functions would most prominently define the temporal axis or how they would distribute in tissue, organ, or cell space. Analysis across all tissues found three classes of temporal drivers:

1) Universal: PC3 captured a strong global time component (Fig. 1b, *z*-axis) that was explained at the gene level by widespread diminution in cell proliferation machinery and early erythroid markers (Extended Data Fig. 5c). The top 100 PC3 positive-loading genes are highly enriched for mitotic cell cycle components (Gene Ontology (GO) *P* = 3 × 10^−13^) that map to expression cluster 21 (Fig. 1c, Supplementary Note 1, Supplementary Fig. 1) which, in turn, maps to the stromal and early erythroid cell types previously reported^11^ (Fig. 1d, red boxes). Furthermore, their stromal cell marker set is itself enriched in cell cycle genes (*P* = 1.8 × 10^−13^, cell cycle) and the reverse is also true. Thus the universal transcriptome time axis of PC3 can be explained, at least in part, by gradual system-wide disappearance of circulating primitive erythrocytes and a decrease in the relative proportion of proliferating stromal cells across many tissues and organs.

2) Specification and differentiation: the most numerous and diverse temporal drivers reflect cell differentiation pathways. For example, PC5 is prominent in differentiating the skeletal muscle systems of limbs and face (*P* = 3 × 10^−12^), with the high-PC5-loading cluster 2 containing genes that are turned on as myogenesis progresses (Fig. 1c, Supplementary Note 1, Supplementary Fig. 1). Neuronal and glial differentiation in CNS tissues is highlighted in PC1 (*P* = 2 × 10^−^^22^), prominently marking genes of cluster 34 (Supplementary Note 1, Supplementary Fig. 1), that are further parsed from single-cell marker distributions by cell sub-type (Fig. 1d).

3) Inter-tissue cell migration: migratory cell populations, either invading or exiting, are important for the development of many tissues, as detailed further below using scRNA-seq data of the limb. At whole-tissue resolution, examples include a blood component (for example, PC2 *P* = 3 × 10^−35^) that emerges prominently in the haematopoietic tissue of origin (liver) and then in other tissues (Fig. 1c, cluster 10 in Supplementary Note 1, Supplementary Fig. 1), while genes that mark maturing B cells^15–18^ in cluster 10 appear in liver, and then in tissues with developing lymphatics (Extended Data Fig. 5b).

## Additional data structure

Much additional dynamic and biological structure is summarized schematically at the major cluster level and is annotated further for individual clusters and sub-clusters (Extended Data Fig. 4, Supplementary Note 1, Supplementary Fig. 1). The anterior–posterior spatial axis was enriched in six of the top 20 PCs of different *Hox* cluster members expressed according to their known positional codes (Supplementary Data 1, 2, expression clusters 19 and 25 in Supplementary Note 1, Supplementary Fig. 1). Reanalysing specific gene groups of interest, such as transcription factors (Extended Data Fig. 7a–e), or applying speciality algorithms can provide additional insights such as anti-correlations of microRNAs with predicted polyA-RNA targets^19^. To evaluate additional effects of metadata features on transcriptome structure, we applied canonical correlation analysis^20,21^ (CCA, see Methods), which identified dissection-based batch effects and sex-specific expression that may be pertinent to some future data uses (for example, differential amounts of maternal blood; thymic contamination of some lung and heart samples; sex-biased samples from embryos of different sex) (Extended Data Figs. 1a, 8, Supplementary Data 3).

## Transcription factor motif topology

**Fig. 2:**
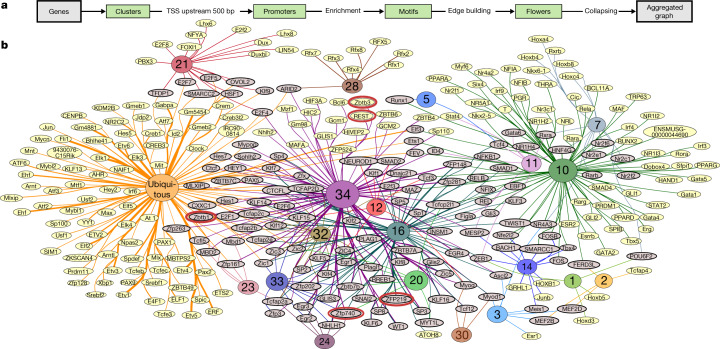
Promoter motif codes for dynamic expression clusters of Fig. 1. **a**, Flowchart for motif enrichment analysis. **b**, A computed graph summary of unique and shared TF recognition sequence motifs. TF motif nodes are labelled as in the CIS-BP database^55^ where all uppercase indicates a human-derived motif and mixed case indicates a mouse-derived motif, and the respective gene cluster source nodes are coloured and numbered per the gene expression clustering in Fig. 1c. Edges connect a motif node with the expression cluster node(s) in which it was enriched, with edge thickness indicating significance (–log_10_*P*). Grey, motifs enriched in more than one cluster; yellow, unique enrichment. The size of each source expression cluster node is proportional to the scaled number of genes in the corresponding cluster.

The patterns of RNA co-expression revealed by clustering (Fig. 1c, Supplementary Note 1, Supplementary Fig. 1) are caused in part by transcriptional co-regulation. Elevated frequencies of TF recognition sequence motifs in promoters of co-expressed genes can computationally link specific TFs or TF families to their likely target genes and regulatory elements. We tested the proximal promoters (500 bp upstream of the transcription start site (TSS)) of all genes in each expression cluster (numbered according to the expression cluster origin in Fig. 1c) for enrichment of all known consensus TF binding motifs (718 motifs; see Methods). A bipartite graph was constructed to identify local and global relationships between the resulting combinatorial motif codes and their source expression clusters (Fig. 2). First, the resulting 307 significantly enriched motifs displayed expected local relationships: fetal liver cluster 10 is characterized by haematopoetic (GATA1, GATA2, RUNX1, BCL11A) and hepatic (SMAD1, PPARG, NR1H2) markers; the highly specific Rfx factor family marks its cilium cluster (cluster 28); and the E2f family is prominent in the previously discussed cell cycle-themed cluster 21 (Supplementary Note 1, Supplementary Fig. 1, Supplementary Data 5).

The graph topology also shows binary and higher-degree motif code-sharing (grey shaded nodes) that selectively connects specific expression cluster promoter nodes from Fig. 1c with each other, suggesting that they jointly use identical or paralogous TFs. At a high level, the prominent separation of neurogenesis (cluster 34) from haematopoiesis (cluster 10) first observed in the transcriptome emerged independently for the motif codes, with only two shared motifs between them, whereas many other clusters share numerous motifs with each of them and with each other. The ubiquitous expression cluster had the strongest and most numerous motif enrichments in the entire transcriptome, with extensive representation of the Ets and Cre families (Fig. 2b, Extended Data Fig. 10e). Enrichment and occupancy of these families have previously been associated with housekeeping genes in humans^22,23^. Finally, the most extensive code-sharing among expression clusters was with CNS neuronal cluster 34, which connects with many other clusters of diverse tissue origins and functional themes (Figs. 1c, 2b). A plausible explanation for this CNS-centric sharing pattern is that many involved TFs (and/or their paralogues) were recruited during evolution to new uses that support increasing mammalian neuronal diversity.

## Cluster-specific regulatory mechanisms

The transcriptome structure and corresponding promoter motif resource provide entry points for identifying cluster-specific regulatory mechanisms. For example, integrating our transcriptome and global epigenomic maps across matched samples showed that the upregulated brain cluster 34 has strong repressive histone mark density (H3K27me3) at early developmental times that declines as its RNA expression trajectories rise (Extended Data Fig. 9a, e). Subsequent global quantification of developmental differentials in H3K27me3 promoter signal relative to RNA output across all clusters found that brain clusters 30, 32 and 34 stand out as candidates for a H3K27me3-mediated de-repression mechanism, even though many other clusters have similarly rising RNA trajectories (Extended Data Fig. 9a). Our previous DNA motif enrichment analysis showed that the neuronal repressor *Rest* (also known as *Nrsf*) motif is specifically and strongly enriched in cluster 34 promoters (Fig. 2b). The putative targets of REST, inferred from an independent ChIP–seq study^24^, are also specifically enriched in cluster 34 (Extended Data Fig. 9b); the expression of *Rest* RNA decreases in brain tissue over time (Extended Data Fig. 9c); and REST-occupied promoters^24^ show even greater H3K27me3 signal enrichment at early times (Extended Data Fig. 9f), all of which is consistent with a significant role for REST in CNS-focused de-repression. This in vivo result is consistent with the results of an earlier in vitro study of neural progenitors^25^, but not with those of an embryonic stem cell study that reported no H3K27me3 enrichment at REST locations^26^. Beyond REST, other candidate repressors whose motifs are enriched in clusters 34 and/or 32 also exhibit expression trajectories that diminish as development progresses (for example, *Zfp219*, *Zbtb1*, *Zbtb3*, *Zfp740*; red oval outlines, Fig. 2b) while additional presumptive C2H2 zinc finger transcriptional repressors whose recognition motifs are unknown are concentrated in the CNS-enriched expression cluster 33 (Extended Data Fig. 7e) with overall downward expression trajectories (Supplementary Note 1, Supplementary Fig. 1). Our working model is that these repressors provide additional targeting diversity and specificity for the pervasive H3K27me3-mediated repression and de-repression process in the developing brain. This will become testable as their individual binding targets and derived motifs are determined (https://www.encodeproject.org/matrix/?type=Experiment&status=released&assay_title=TF+ChIP-seq&award.rfa=ENCODE3&award.rfa=ENCODE4&lab.title=Michael+Snyder%2C+Stanford&lab.title=Richard+Myers%2C+HAIB). In a separate analysis, we examined the large ubiquitous cluster and found evidence suggesting that a post-transcriptional mechanism has a substantial role in setting divergent levels of expression within the ubiquitous cluster (Extended Data Fig. 10).

## Histogenesis at single-cell resolution

**Fig. 3:**
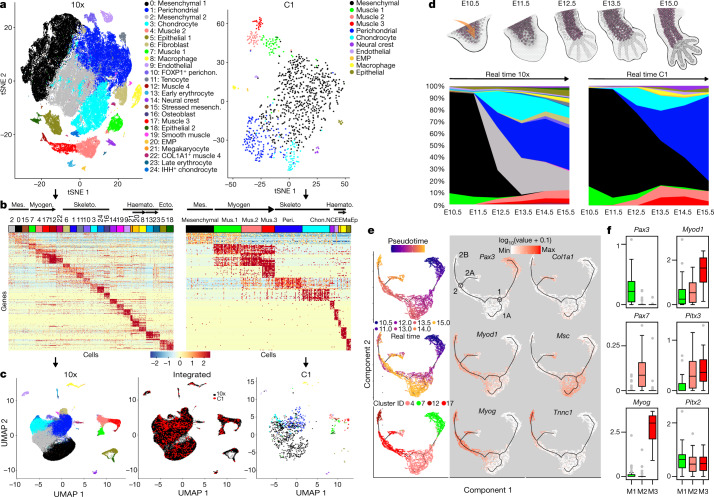
Single cell analyses of forelimb histogenesis. **a**, Two-dimensional *t*-SNE of cell clusters, of 10x (left, *n* = 90,637 cells) and C1 data (right, *n* = 920 cells). Colours indicate provisional cell identities as in Supplementary Note 2. **b**, Cell cluster marker genes (top 15 per cluster), down-sampled for display to 100 cells per cluster for 10x and 30 cells for C1 data. Mes., mesenchymal; Myogen, myogenesis; Skeleto., skeletogenesis; Haemato., haematopoiesis; Ecto, ectoderm; Mus. 1–3, muscle 1–3; Peri, perichondrium; Chon., chondrocytes; NC, neural crest; EE, endothelium and EMP; Ma, macrophage; Ep, epithelium. **c**, Integrated visualization of 10x (left) and C1 (right) single cells on a 2D UMAP plane, separately or jointly projected (centre; see text and Methods). **d**, Limb development schematic (arrow indicates immigrating lineages) and cell type composition plotted as a time series. The colour code corresponds to cell clusters in **a**. **e**, Monocle lineage inference model for skeletal myogenesis. Pseudotime, developmental time and cell type (left); informative marker gene expression mapped on the right. *n* = 7,668 muscle cells. **f**, Box plots of Boolean, graded, and pan-lineage pattern TFs; *n* = 23 muscle 3 cells; *n* = 38 muscle 2 cells; *n* = 54 muscle 1 cells. Boxes, 25th–75th percentiles; centre, median; whiskers, 1.5 × interquartile range.

**Extended Data Fig. 2:**
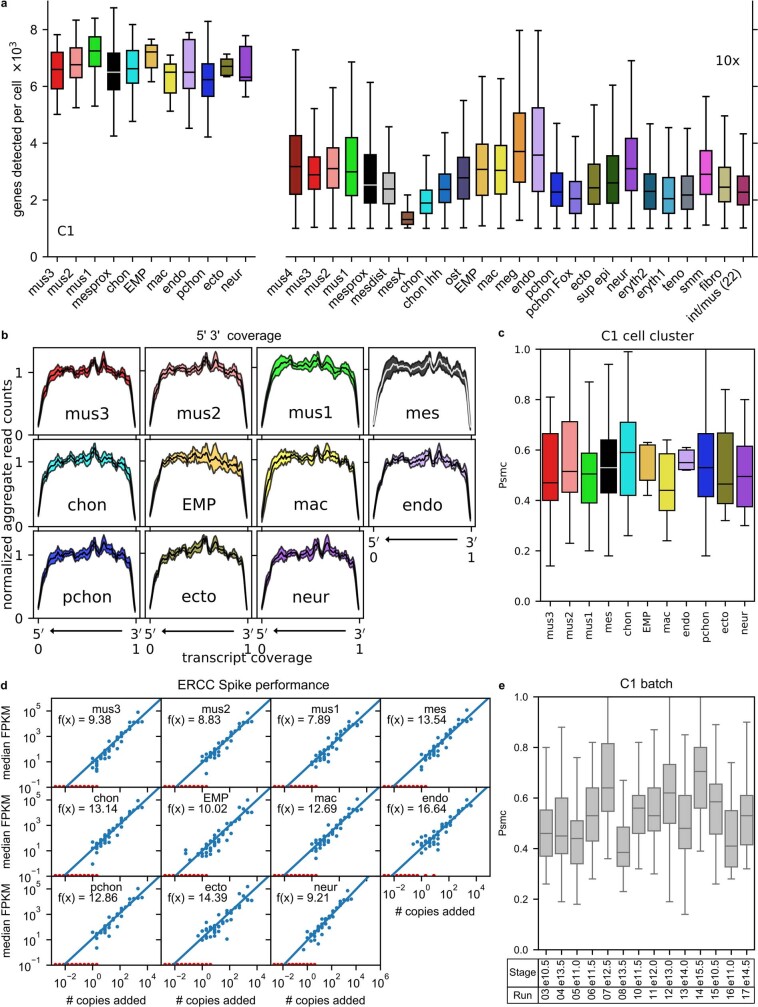
Quality metrics of bulk RNA-seq and scRNA-seq II. **a**, Numbers of genes detected among each cell type defined by the C1 platform (mus3, Muscle 3; mus2, Muscle 2; mus1; Muscle 1; mesprox, Mesenchymal; chon, chondrocyte; EMP, EMP; mac, Macrophage; endo, Endothelial; pchon, Perichondrial; sup epi, Epithelial; neur, Neural crest) and the 10x platform (mus4, Muscle 4; mus3, Muscle 3; mus2, Muscle 2; mus1, Muscle 1; mesprox, Mesenchymal 1; mesdist, Mesenchymal 2; mesX, Stressed mesenchymal; chon, Chondrocyte; chon Ihh, Ihh+ chondrocyte; ost, Osteoblast; EMP, EMP; mac, Macrophage; meg, Megakatyocyte; endo, Endothelial; pchon, Perichondrial; pchon Fox, Foxp1+ perichondrial; ecto, Epithelial 1; sup epi, Epithelial 2; neur, Neural crest; eryth2, Late erythrocyte; eryth1, Early erythrocyte; teno, Tenocyte; smm, Smooth muscle; fibro, Fibroblast; int/mus (22), Col1a1+ muscle 4). Left: *n* = 23 mus3 cells; *n* = 38 mus2 cells; *n* = 54 mus1 cells; *n* = 571 mesprox cells; *n* = 57 chon cells; *n* = 5 EMP cells; *n* = 10 mac cells; *n* = 7 endo cells; *n* = 139 pchon cells; *n* = 8 ecto cells; *n* = 8 neur cells. Right: *n* = 404 mus4 cells; *n* = 1764 mus3 cells; *n* = 3,625 mus2 cells; *n* = 1,875 mus1 cells; *n* = 22,925 mesprox cells; *n* = 17,205 mesdist cells; *n* = 114 mesX cells; *n* = 10536 chon cells; *n* = 494 chon Ihh cells; *n* = 86 ost cells; *n* = 238 EMP cells; *n* = 1,123 mac cells; *n* = 29 meg cells; *n* = 1,011 endo cells; *n* = 20,254 pchon cells; *n* = 912 pchon Fox cells; *n* = 2,719 ecto cells; *n* = 629 sup epi cells; *n* = 577 neur cells; *n* = 188 eryth2 cells; *n* = 425 eryth1 cells; *n* = 762 teno cells; *n* = 210 smm cells; *n* = 2,204 fibro cells; *n* = 328 int/mus(22) cells. **b**, Transcript coverage from 5′ to 3′ (left to right on *x* axis) in C1 single-cell libraries is uniform and consistent across the 11 different cell types. The *y*-axis is normalized, aggregate read counts. The centre values are median values for each bin; the shading represents standard deviations for each bin. *n* = 23 mus3 cells; *n* = 38 mus2 cells; *n* = 54 mus1 cells; *n* = 571 mesprox cells; *n* = 57 chon cells; *n* = 5 EMP cells; *n* = 10 mac cells; *n* = 7 endo cells; *n* = 139 pchon cells; *n* = 8 ecto cells; *n* = 8 neur cells. **c**, Probability of single-molecule capture (Psmc) estimates for each of the 11 different C1 cell types. *n* = 23 mus3 cells; *n* = 38 mus2 cells; *n* = 54 mus1 cells; *n* = 571 mesprox cells; *n* = 57 chon cells; *n* = 5 EMP cells; *n* = 10 mac cells; *n* = 7 endo cells; *n* = 139 pchon cells; *n* = 8 ecto cells; *n* = 8 neur cells. **d**, Estimated input (*x*-axis) and output (*y*-axis) amounts of ERCC spikes in each cell type. One cell is represented by one dot. The slopes of the fitted lines in log space have been labelled in each panel. **e**, Psmc estimates for each C1 run. Error bars are standard error. *n* = 23 mus3 cells; *n* = 38 mus2 cells; *n* = 54 mus1 cells; *n* = 571 mesprox cells; *n* = 57 chon cells; *n* = 5 EMP cells; *n* = 10 mac cells; *n* = 7 endo cells; *n* = 139 pchon cells; *n* = 8 ecto cells; *n* = 8 neur cells. All box plots are as in Fig. 3f.

**Extended Data Fig. 3:**
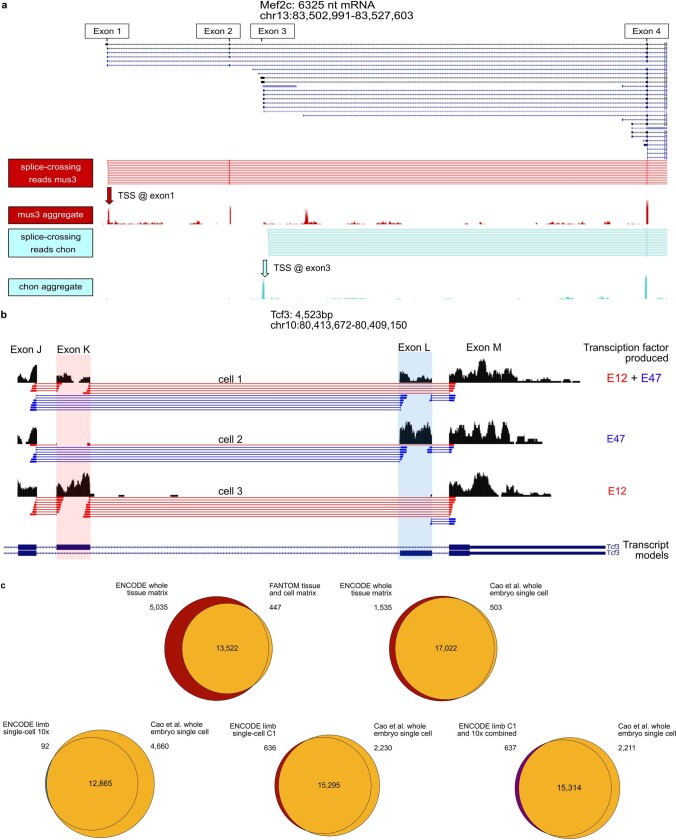
Quality metrics of bulk RNA-seq and scRNA-seq III. **a**, Cell-type-specific TSS choice for Mef2c in the developing limb identified by short-read RNA-seq. UCSC genome browser tracks display Fluidigm C1 data from muscle3 (dark red) and chondrocyte (cyan) cells at Mef2c with Gencode VM20 gene and transcript models. Splice-crossing reads document exon1/2, 2/4 (red) and 3/4 junctions. Aggregate signal tracks for mus3 and chon show that the TSS at exon 1 is used in mus3, whereas chondrocytes select the TSS at exon 3. Median expressed level for MEF2c in muscle3 cells 53.4 FPKM; in chondrocytes 40.3. **b**, Alternative splice choices in different single mesenchymal cells of the developing limb result in alternate forms of Tcf3 (E12 and E47 bHLH TFs) with different DNA binding specificities. Individual splice-crossing reads are displayed beneath the read tracks for each of 3 separate exemplar cells. **c**, Comparisons of whole tissue and single-cell transcriptome gene content with external whole tissue and single-cell resources^10,11^. For all datasets, comparisons were restricted to only protein-coding genes that were detected.

**Extended Data Fig. 4:**
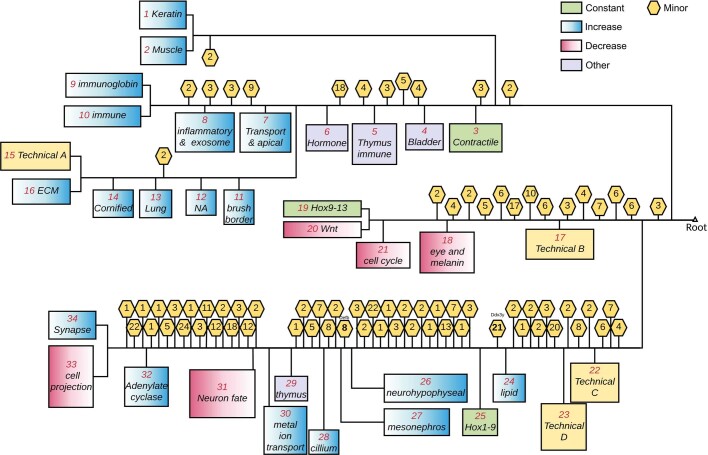
Summary of expression cluster dynamics and dominant functional themes for bulk RNA clusters. Rectangles represent major gene expression clusters with more than 30 members, labelled by the dominant features based on GO analysis, tissue specificity and gene class are labelled. Blue boxes indicate increase over time; pink decreases over time; green reflect relatively constant levels; lavender lacks coherent time course dynamics; yellow represent likely technical issues. The remainder are small clusters (<30 genes), labelled as hexagons with the cluster size given.

**Extended Data Fig. 5:**
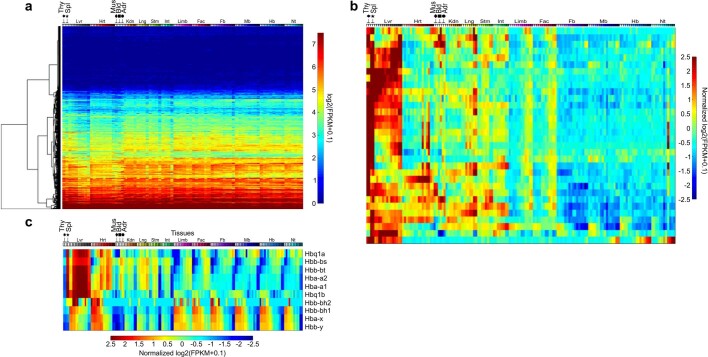
Additional groups of genes with diverse biological implications. In all plots, tissue identities are labelled on top (*x*-axis) matching Fig. 1, and genes are on the *y*-axis. **a**, Expression levels of ubiquitous genes are shown in the heat map according to the scale bar at right. **b**, **c**, normalized expression levels of genes associated with B-cell activation in cluster 10 (**b**) and haemoglobin genes (**c**).

From E10 to E15.5, the developing forelimb progresses from a simple limb bud composed mainly of undifferentiated mesoderm to a highly patterned structure with distinct skeletal, muscular, vascular, haematopoietic and dermal tissue systems (Fig. 3). We collected two types of scRNA-seq data (Fig. 3), each spanning the same time points as the parent bulk tissue study: 1) 920 cells from the C1 platform, sequenced to relatively high depth (about one million reads per cell), which achieved sensitive RNA detection rates, and full-length transcript coverage that was comparable with the bulk data (Extended Data Figs. 1c, e, 2a–e, 3a, b); and 2) about 90,000 cells from the 10x Genomics 3′end-tag platform, which expanded cell-type discovery (Extended Data Figs. 1c–e, 2a). In the higher-resolution data, we detected 15,931 protein-coding genes and 938 lncRNAs, of which 91% and 71%, respectively, overlapped with the limb whole tissue time-course (Extended Data Fig. 1c), while the 10x data captured 81% and 36%, respectively. Comparison of these data with published whole embryo scRNA-seq data^11^ showed the expected overlap of cell-type relationships (Extended Data Fig. 11b) coupled with a notably high overlap of expressed genes in which 15,314 protein-coding genes were in common and only 2,230 and 637 were found only in the whole embryo or in the forelimb, respectively. This is consistent with greater cellular breadth in the whole embryo study versus deeper cellular and molecular coverage in the forelimb study (Extended Data Figs. 1d, e, 2a).

## Resident and immigrating cell types

Clustering the most differentially expressed genes across all cells identified major progenitor and differentiating cell types and showed similarity relationships between them (Fig. 3a–c, Extended Data Figs. 11, 12; see Methods). Provisional cell identity assignments were based on GO enrichment analysis together with support from the published developmental studies for previously reported ‘marker’ genes (Supplementary Note 2, Supplementary Figs. 2, 3; Supplementary Tables 1, 2; references and discussion of marker gene limitations therein; Fig. 3a, b). Major cell types in both studies included resident limb-bud mesenchyme and its chondrogenic and osteogenic derivatives, plus independently immigrating lineages that give rise to myogenic, monocyte/macrophage, endothelial or neural crest derivatives. These 10x data also provided evidence for 14 more cell types or states. When projected into the whole-tissue transcriptome and compared with similarly projected whole-embryo scRNA-seq data, this deeper and more focused limb sampling showed lineage subdivisions and sharpening of some types compared with the whole embryo (for example, myocytes, connective progenitors, limb mesenchyme; Extended Data Fig. 11b).

## Lineage progression and inference

Whole-transcriptome *t*-SNE and uniform manifold approximation and projection (UMAP) and phylogenetic clustering analyses segregated cell types (Fig. 3a–c, Extended Data Figs. 11c, d, 12a) whose trajectories through time were then mapped (Fig. 3d). The extent of under-representation of large multinucleated myotubes, together with other possible disaggregation, differential cell capture and survival, and stochastic sampling artefacts, were assessed relative to unperturbed whole-limb RNA data using CIBERSORT^27^ to produce an adjusted tissue proportion model (Extended Data Fig. 12b, c).

Computed UMAP and Monocle lineage models (Fig. 3c, e, Extended Data Fig. 11c, d) were mainly consistent with classical and modern tracing studies and inferences from genetic knockouts, while also identifying new relationships and associated regulators. In the myogenic system, early progenitors require the TF PAX3 to migrate into the limb bud from adjacent axial somites^28–30^, and *Pax3* is indeed the strongest differential gene defining the Muscle1 cell cluster (Wilcoxon rank sum test: 3.7-fold enrichment in 10x data and 16.7-fold in C1 from both data-types), which mapped to the earliest Monocle pseudo-time group (Fig. 3e). The stages in the progression and inferred relationships among stages are defined by overall correlation patterns among differentially expressed genes (Extended Data Fig. 11a, b), while specific marker genes from the myogenesis literature provided biological interpretation and hypothesis generation (Fig. 3e, Extended Data Fig. 11d).

The Monocle myogenic lineage model showed two branch points (Fig. 3e). The first (in both real time and pseudotime) produces branch 1A, consistent with an important known population of muscle stem cells that later give rise to the regenerative cells of adult muscle. They are marked by the genetically pertinent PAX7 regulator (Extended Data Fig. 11d), and its direct target MSC (Fig. 3e), which represses myocyte differentiation^31,32^. From branch point 2, one arm leads to expected mature myocytes marked by *Tnnc* (branch 2B), whereas branch 2A was not expected. It models a cell population that expresses signatures of interstitial muscle fibroblasts (IMFs)^33^, such as *Col1a1* and *Osr1*/*2*, in addition to classic myogenic markers such as *Myod1* and *Myog* (Fig. 3e, Extended Data Fig. 12d). We confirmed that individual cells in the developing forelimb co-immunostained for muscle and IMF marker proteins (Extended Data Fig. 12e). This phenotype resembles the small and somewhat mysterious 10x cluster 22, and a second Monocle model incorporating cluster 22 supports that interpretation (Extended Data Fig. 12d). Considered in the light of earlier evidence that adult tissue IMFs have latent myogenic capacity^34–36^, this raises questions about their developmental origin (from resident mesenchyme or PAX3^+^ precursors); adult fate (whether to become an adult IMF and/or maintain myogenic potential); and biological importance. More broadly, we confirmed and extended previous microarray results on populations of muscle precursor cells enriched by fluorescence-activated cell sorting (FACS)^37,38^ and recent scRNA-seq of PAX3–GFP-selected cells^39^. Our Monocle myogenesis models share some basic characteristics with the pioneering one constructed by Trapnell and colleagues^40^, although the models also reflect substantial differences between adult human muscle regeneration in vitro and fetal mouse myogenesis in vivo.

Within the haematopoetic lineage, we identified both erythro-myeloid progenitors (EMPs) and macrophages at early stages of limb development, aided by their exceptionally robust sets of marker genes (Supplementary Note 2, Extended Data Fig. 12a), which is consistent with limb macrophage developing from limb-resident EMPs (Extended Data Fig. 11c) in situ. Finally, the skeletogenic system and its resident mesenchymal progenitors are the largest limb component throughout the time course. Condensation, expansion and differentiation into cartilage and bone is the primary fate of the resident limb mesenchyme^41–43^, represented here by UMAP (Fig. 3c) and Monocle models (Extended Data Fig. 11c) that focus on putative chondrocytes and fibroblast/perichondrial cells that form two dominant branches from the mesenchyme. The structure detected is much less clearly partitioned and ordered than was myogenesis, and a more refined single-cell-resolved model of skeletogenesis will probably require more focused cell sampling coupled with spatial genomics to capture additional anatomical clues^44–47^.

## *Trans*-acting cell-type TF networks

Each cell type cluster has a substantial set of differentially expressed TFs (Supplementary Data 4). In the myogenic lineage, these differential TFs were expressed in three modes with different regulatory and lineage inference implications (Fig. 3f, Extended Data Fig. 12f, Supplementary Fig. 3): 1) sharply stage-restricted Boolean patterns separate cell stages from each other, including the well-known causal transcription regulator genes *Pax3*, *Pax7*, *Msc*, and *Myog*; plus newly added ones such as *Sp5* and *Sox8*; 2) a few lineage-restricted uniformly expressed regulators whose expression pattern defines the entire lineage (*Pitx2* and *Six1*); and 3) multi-stage TFs with graded expression levels, such as *Myod1* and *Pitx3*, whose expression joins two or more stages together, while nevertheless discriminating stages quantitatively (Fig. 3f). Some regulators, including TFs that are widely understood to function only at later stages in the lineage, were detectably and precociously expressed at low levels, but only in the more sensitive C1 data (Fig. 3f, Extended Data Fig. 12f). For example, low level expression of *Myod1* is detected in *Pax3*-expressing cells ahead of  well-known myoblast- and myocyte-stage MYOD1 functions^48^. This implies that the *Myod1* locus is already open at this point, and visualization of the ENCODE DHS histone mark data at E10.5 identified specific distal and promoter-proximal sites that support this idea (Extended Data Fig. 15b).

**Fig. 4:**
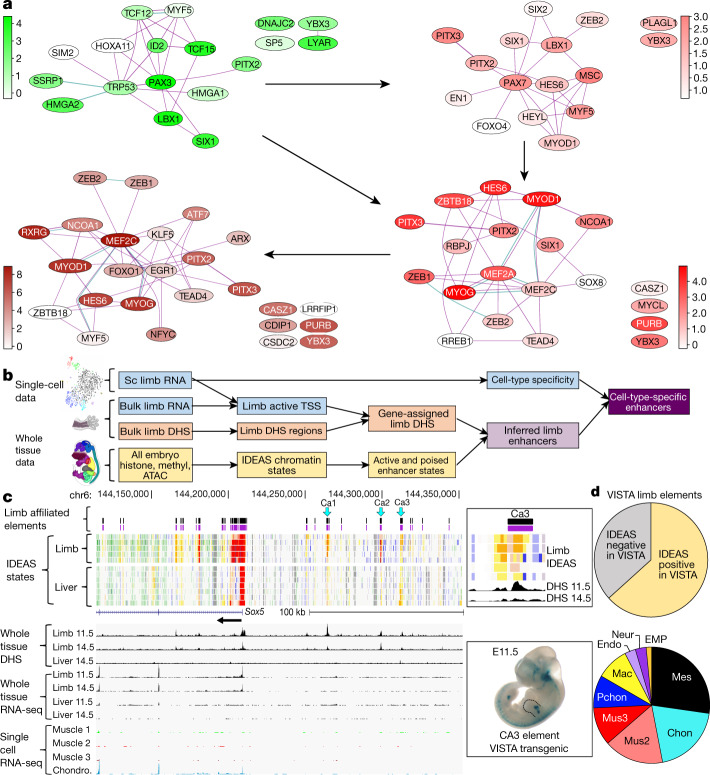
*Trans*-acting and *cis*-acting regulatory networks inferred for specific limb cell types. **a**, STRING networks of skeletal muscle lineage for cell-type differential TFs from 10x data (see Methods); edges are coloured by types of STRING evidence (cyan for database and magenta for experimental); nodes coloured according to 10x RNA-seq levels; arrows indicate lineage transitions (see text). **b**, Schematic for discovering cell-type enhancer and promoter elements using scRNA-seq and IDEAS chromatin state elements defined in whole tissue chromatin assays (see text, Methods and Extended Data Fig. 11a). **c**, Candidate upstream limb skeletal enhancers (CA1–CA3) for *Sox5* with in vivo enhancer data from VISTA for a CA3-containing segment at right (https://enhancer.lbl.gov/cgi-bin/imagedb3.pl?form=presentation&show=1&experiment_id=895&organism_id=1). Computed IDEAS limb cell-type elements (purple track); IDEAS epigenomic segmentation tracks below with poised and active enhancer type (orange) and promoter type (red) states below. **d**, Summary of IDEAS and scRNA-seq cell-type elements in the VISTA resource. Top, IDEAS limb elements in VISTA, *n* = 235/371 (63%). Bottom, VISTA-positive IDEAS elements by cell-type (*n* = 66 cell-type-specific elements).

We used known protein and genetic interactions to organize all cell-type differential TFs into their respective interaction networks (myogenic lineage Fig. 4a; all other cell type clusters Supplementary Note 3, Supplementary Figs. 4–7), showing that pan-lineage and graded factors extensively switch interacting partners across stages of the myogenic lineage progression. The inference leverage provided by the low-level graded-pattern genes was platform sensitive, with the higher sensitivity of the C1 data detecting anticipatory (and also trailing) expression in sequential stages that had escaped detection in our 10x data (Extended Data Fig. 12f).

## *Cis*-acting cell-type elements

The companion ENCODE whole-tissue histone modification, chromatin accessibility and DNA methylation datasets provide rich biochemical signatures from which candidate regulatory elements can be computationally inferred at the whole-tissue level^2,13,14^, but they lack cell-type resolution. To parse elements that are selectively active according to cell type or state (Fig. 4b), we first defined the boundaries of biochemically active sequence elements using the companion limb DNase peak calls. We then applied IDEAS^13,14^ to learn and summarize epigenomic features over fixed genomic segment bins, and extracted those DNase peaks that overlapped with active and bivalent IDEAs bins (the bivalent bins include both poised elements and active signals from minor cell types diluted by cells with alternative signatures). We assigned an element to a cell type on the basis of the differential expression of its associated gene measured by scRNA-seq. Summing the active and bivalent signatures, among 2,208 cell-type and lineage-specific genes, 2,018 (91.4%) had at least one affiliated active or poised element among the total collection of 22,230 (Supplementary Data 6). Individual loci with multiple candidate elements, plus supporting IDEAS state tracks, developmental DHS and RNA expression patterns, are shown for biologically important chondrogenic, myogenic and macrophage examples (Fig. 4c, Extended Data Figs. 13b, 14a). On the basis of our overall element recovery and prior limb tissue reconstruction results (Fig. 3d, Extended Data Fig. 12b, c), we estimate that the whole limb epigenomic data have the sensitivity to identify validated cell type enhancers for cells that comprise less than 5% of the starting population.

We evaluated all elements in the collection that overlapped with the independently derived VISTA transgenic mouse database of empirically tested candidate *cis*-regulatory elements. For this overlapping set, 63% were validated as active VISTA enhancers (https://enhancer.lbl.gov/) distributed across our major cell types^2,49^ (Fig. 4d). We did not expect all IDEAS overlapping elements to have scored positively in the VISTA assay paradigm for reasons summarized in the accompanying paper^2^ and because of VISTA’s narrower developmental time-window (E11.5–E12.5, compared to E10.5–E15 for our data). VISTA’s spatial domains typically included limb LacZ transgene staining but often showed added staining elsewhere in the embryo. This is expected, as our major cell types are represented elsewhere in the body and are not restricted to the limb. Conversely, some spatially patterned limb elements in VISTA (for example, Mm1505 and Mm1492; Extended Data Fig. 13b) do not appear limited to a cell type, and so are not in our collection. Compared with the mouse FANTOM candidate enhancer and promoter sets, which were computed from CAGE data and cover a much wider sampling of tissues^10^ (http://fantom.gsc.riken.jp/5/), our entire limb IDEAS set overlaps with 44% and 30% of all FANTOM promoters and enhancers, respectively. Of these, 14% of each (9,943 promoters and 2,147 enhancers) are in our cell-type collection. Another large group of ours (20,119 and 19,384 IDEAS cell-type enhancers and promoters) were not in the FANTOM database, which is overall a smaller collection (Extended Data Fig. 15a).

Transcription factor binding motifs enriched in cell-type IDEAS distal elements (more than 2 kb from the affiliated transcription start site (TSS)) or in promoters (Supplementary Data 5, 6), were organized in computed graphs that revealed lineage-related cluster nodes joined to each other by motif sharing across stages and related cell types (that is, muscle clusters 4, 12, 17; haematopoetic clusters 8, 13, 20, 21 in Extended Data Fig. 14b). Neural crest stood out for its large number of distal motifs, including many Hox family members, that are likely to reflect their use of positional signalling gradients for specification and migration. We similarly extracted motif codes for genes whose expression is significantly depleted in a cell-type-specific manner. Such genes were especially prominent in early haematopoetic cells, and their promoters were strikingly enriched in repressor and Hox motifs. We speculate that cells that traverse the entire embryo silence genes that, in other cell types, actively respond to positional signalling.

Overall, an advantage of the ENCODE fetal transcriptome compared to prior conceptually similar efforts is the opportunity to integrate companion epigenome and microRNA resources^2,19,50,51^. In the limb example above, we have shown that scRNA-seq can be used to decompose the tissue-level epigenome according to cell type, an approach that could be generalized and further strengthened by integrating single-cell assay for transposase-accessible chromatin using sequencing (scATAC–seq) together within more sophisticated algorithms^52–54^.

## Methods

No statistical methods were used to predetermine sample size. The experiments were not randomized and investigators were not blinded to allocation during experiments and outcome assessment.

### Bulk RNA-seq from mouse embryo tissues

Pulverized pooled mouse embryo tissue replicates from time points E10.5, E11.5, E12.5, E13.5 E15.5 and E16.5 were received from the Ren laboratory, which supplied these tissues for the entire mouse development project^50^. E14.5 and P0 tissues were dissected from single animals at Caltech. Replicate tissue samples were lysed and extracted using the Ambion mirVana protocol (AM1560). Residual genomic DNA was removed using the Ambion Turbo DNA-free kit (AM1907). Total RNA was quantified with Qubit and RIN values were collected with the BioAnalyzer Pico RNA kit (5067-1513). The median RIN value was 9.7 (CV = 4.4%). Each cDNA library was built using 10 ng total RNA spiked with ERCC spikes (AM4456740) diluted 1:5,000 in UltraPure H_2_O (InVitrogen 10977023) containing carrier tRNA (AM7119) at 100 ng/μl, RNase inhibitor (Clontech 2313A) at 1 unit/μl and DTT (Promega P1171) at 1 mM. cDNA was reverse-transcribed and amplified according to the protocol in the SMARTer UltraLow RNA kit for Illumina (634935) using Clontech SMARTScribe reverse transcriptase (639536), and TSO, dT priming and amplification primers from the Smart-seq2 protocol 5. The first-strand product was cleaned up on Ampure XP beads, and then amplified using the Clontech Advantage 2 PCR kit (639207) with 13 PCR cycles and an extension time of 12 min. After a second round of Ampure XP cleanup, the amplified cDNA was quantified on Qubit and the size distribution was checked with the HS DNA BioAnalyzer kit (5067-4626). cDNA libraries were then tagmented using the Illumina/Nextera DNA prep kit (FC 121-1030) with index tags from Illumina (FC 121-1031), cleaned up with Ampure XP beads, quantified on Qubit and sized with the Agilent HS DNA kit. Libraries were sequenced on the Illumina HiSeq 2500 as 100-bp single-end reads to 30M aligned reads depth. Inclusion for ENCODE submission required replicate concordance scores by Spearman correlation of FPKM values >0.9.

### Single-cell transcriptome measurements using the Fluidigm C1 and 10x Genomics v2

One pair of embryonic forelimbs from a single mouse was used at each time point (E10.5, E11.0, E11.5, E12.0, E13.0, E13.5, E14.0, E15.0). After dissection from the carcass, limbs were incubated in a 50 μl droplet of a 10% collagenase solution (Worthington LS004202) for 5 min at 37 °C. The limbs were then visualized under a dissecting scope and the ectoderm was removed manually with a pair of #5 Dumont forceps, which had the effect of reducing epithelial cell representation in the high resolution data. The mesenchymal core of the limb bud was then transferred to a 200 μl droplet of Accumax (AM105), and the dish was reincubated for 15 min at room temperature. The cells were then manually triturated once with a P200 tip to suspend them, and pipetted into 500 μl DMEM + 10% FBS. Limb cells were spun at 500*g* for 5 min at 4 °C, resuspended in 500 μl fresh DMEM + 10% FBS, and passed over a 20-μm mesh (Miltenyi 130-101-812). They were then counted and diluted in DMEM + 10% FBS to achieve a final concentration of 250,000 cells/ml. Twelve microlitres of this suspension was added to 8 μl Fluidigm Cell Suspension Reagent for loading on the Fluidigm IFC (10–17-μm size). Cells were then visually inventoried for doublets and empty chambers, and returned to the C1 for lysis, reverse transcription and amplification using the SMART-Seq v4 protocol. Lysis buffer: 8.6 μl water, 1 μl C1 loading buffer, 2.4 μl Smart-seq2 oligo dT primer (10 mM), 2.4 μl Clontech 10 mM dNTPs, 2 μl ERCC spikes (AM4456740) (diluted 1:40,000 in UltraPure H_2_O (InVitrogen 10977023) containing carrier tRNA (AM7119) at 200 pg/μl, RNase inhibitor (Clontech 2313A) at 1 unit/μl and DTT (Promega P1171) at 1 mM), 0.5 μl 100 mM DTT, 2.6 μl Clontech single-cell reaction buffer. Reverse transcription reaction: 5.6 μl Clontech 10x transcription buffer, 0.6 μl C1 loading buffer, 5.6 μl Smart-seq2 TSO (10 mM), 0.4 μl Clontech RNase inhibitor, 2.8 μl Clontech SMARTScribe. PCR reaction: 4.4 μl water, 4.5 μl C1 loading buffer, 75.2 μl Clontech SeqAmp buffer, 3 μl Smart-seq2 amplification primers (10 mM) and 2.9 μl Clontech SeqAmp polymerase.

Amplified cDNA samples were diluted in 10 μl of C1 DNA dilution reagent, and a 1 μl aliquot of each was quantified on Qubit. Eleven samples from the IFC were selected for BioAnalyzer sizing based on yield and chamber occupancy. An aliquot of the cDNA libraries was diluted to 0.1–0.3 ng/μl using C1 Harvest reagent, and the libraries were then tagmented using the Nextera XT DNA sample prep kit (FC 131-1096) and Nextera XT indices (FC 131-1002). After tagmentation and amplification, libraries were pooled, cleaned up twice with Ampure XP beads (0.9× volume), quantified on Qubit and sized on the BioAnalyzer using the HS DNA kit.

The libraries were then sequenced as 50-bp single reads to a depth of about 1M aligned reads on the Illumina Hi-Seq 2500.

10x Genomics single-cell libraries were prepared from the single-cell suspensions described above, targeting 10,000 cells per library, exactly as described in the manufacturer’s protocol. They were sequenced as 150-bp paired end libraries, to a depth of 400M reads each on the Illumina Hi-Seq 4000.

### Read mapping and quantification

All the whole-tissue RNA-seq and C1 single-cell RNA-seq data were processed through the standard ENCODE pipeline (https://www.encodeproject.org/pipelines/ENCPL002LSE/), which uses STAR to align raw reads against mm10 genome with spikes and quantifies transcript abundances using RSEM, which provides FPKM, TPM and count values. Downstream analyses were mainly done using MATLAB scripts (https://github.com/brianpenghe/Matlab-genomics). 10x single-cell RNA-seq data were processed using CellRanger with a compatible GTF annotation and “--expect-cells 10000”.

### Whole-tissue RNA-seq PCA, CCA and hierarchical clustering

tRNA genes and genes covered by fewer than 10 reads in all tissues were removed. PCA was performed over the log_2_-transformed FPKM values, with 0.1 added as pseudo-counts to unmask relatively lowly expressed transcripts in order to accommodate high sensitivity of whole-tissue RNA-seq assays. *Z*-scores of eigenvalues from PCA were used to visualize ‘PC scores’, while eigenvector coefficients from PCA were used to visualize ‘PC loadings’. Genes with the highest positive values and lowest negative values were used to interpret biological meanings for each PC.

**Extended Data Fig. 6:**
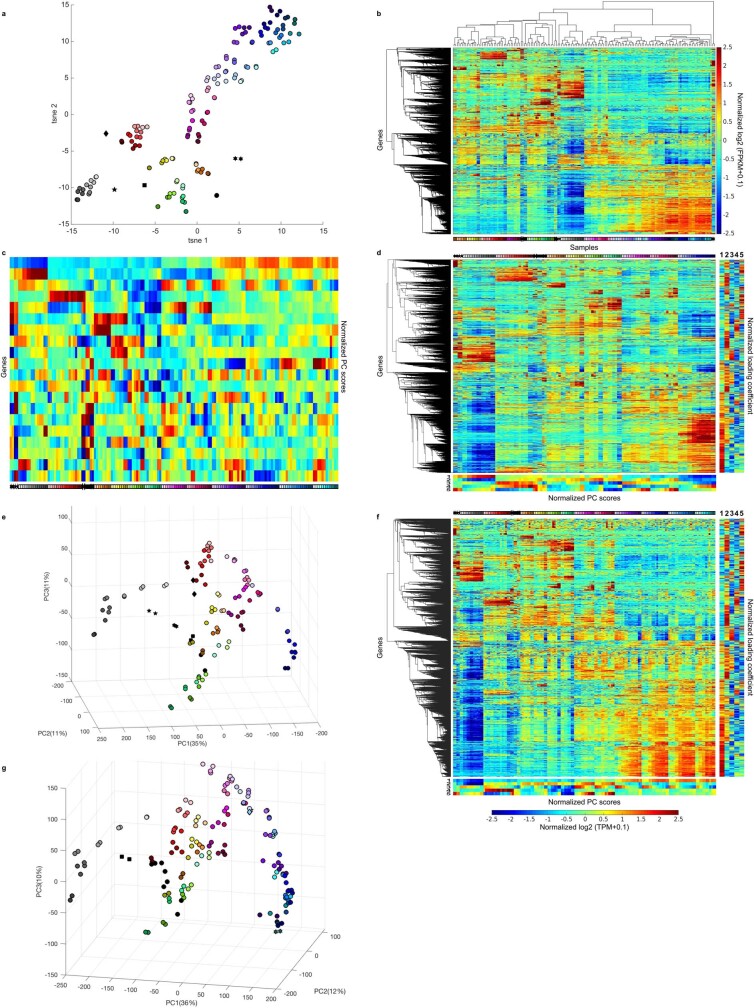
Alternative views of global bulk transcriptome. **a**, Bulk tissue transcriptome is organized on a 2D *t*-SNE plane, with colour code as in Fig. 1. *n* = 156 bulk RNA-seq libraries **b**, Two-way hierarchical clustering of differential genes in bulk data using Pearson correlation. **c**, Normalized principal component scores of the top 20 components. Tissue identities and stages are labelled at the bottom following colour codes in Fig. 1. **d**, **e**, One-way hierarchical clustering (**d**) and PCA projection (PC scores are labelled at the bottom and loading coefficients are on the right) (**e**) of whole transcriptome with forebrains, hindbrains and neural tubes removed to test robustness. *n* = 112 bulk RNA-seq libraries. Colour codes as in Fig. 1. **f**, **g**, One-way hierarchical clustering (**f)** and PCA projection (similar to **e**) (**g**) of whole transcriptome quantified by TPM instead of FPKM. *n* = 156 bulk RNA-seq libraries. Colour codes as in Fig. 1.

**Extended Data Fig. 7:**
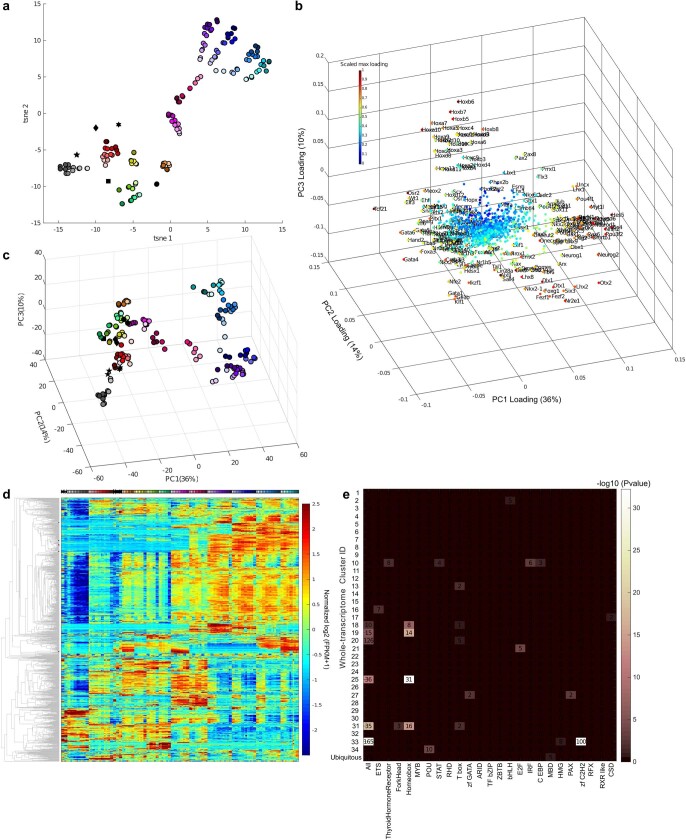
Transcription factor expressions in the bulk data. Colour codes in **a–d** as in Fig. 1. **a**, *t*-SNE representation of transcription factor expression profiles. *n* = 156 bulk RNA-seq libraries. **b**, 3-D projection of PC loading coefficients of transcription factors. **c**, 3-D projection of PC loadings of transcription factor expression profiles. *n* = 156 bulk RNA-seq libraries. **d**, One-way hierarchical clustering of transcription factor expressions in bulk data. Tissue identities are labelled following colour codes in Fig. 1. *n* = 156 bulk RNA-seq libraries. **e**, Abundance representation of transcription factor families in individual bulk expression clusters. Colours indicate Bonferroni-corrected *P* values from hypergeometric test.

**Extended Data Fig. 8:**
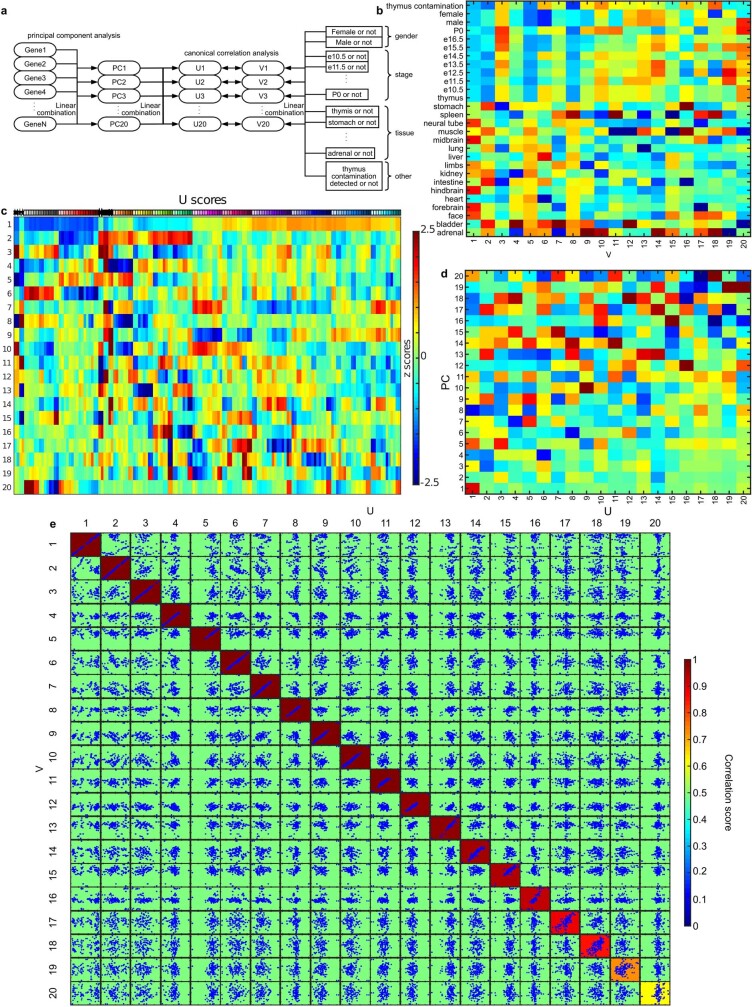
Canonical correlation analysis of the bulk data. **a**, A diagram showing the setup of canonical correlation analysis (more details in Supplementary Data 3). **b**, The vertically normalized loadings of the Boolean metadata variables. Tissue identities are labelled with colour codes in Fig. 1. **c**, The horizontally normalized scores of CCA variables across tissue samples. **d**, The vertically normalized loadings of principal components. **e**, The correlations between U and V variables. Pairwise relationships between U and V variables are shown by the corresponding scatter plots and heat map representing the Pearson correlation coefficient. *n* = 156 bulk RNA-seq libraries.

**Extended Data Fig. 9:**
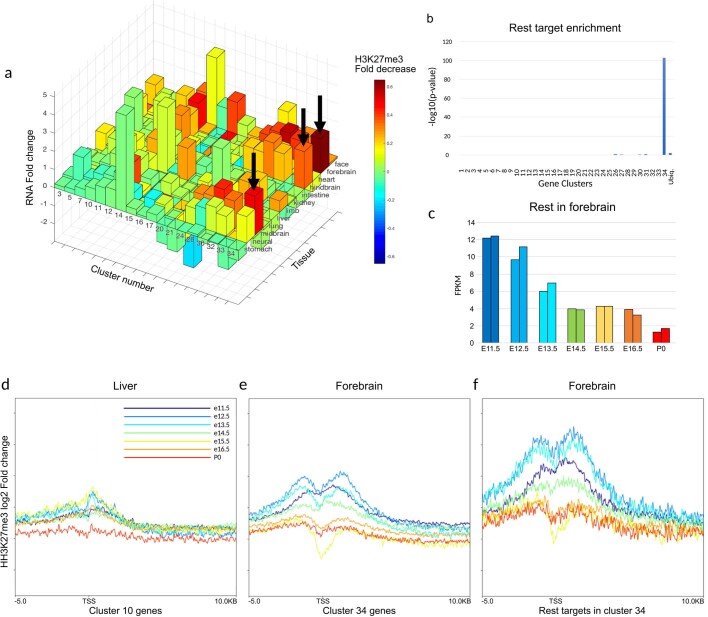
CNS-specific genes are associated with Rest/Nrsf binding and de-repression. **a**, H3K27me3 fold-decrease and RNA fold-change. Each bar represents a cluster of genes in a tissue type. The height represents RNA fold-increase between the earliest and latest time points, and the colours represent H3K27me3 ChIP signal fold decrease. The arrows point to the strongest decrease of H3K27me3 that happens in Cluster 34 in brain samples. **b**, Nrsf target enrichment in individual clusters. Bonferroni-corrected *P* values are calculated based on hypergeometric tests. Sample size (equal to that of Extended Data Fig. 2): Cluster 1 *n* = 121 genes, Cluster 2 *n* = 196 genes, Cluster 3 *n* = 693 genes, Cluster 4 *n* = 65 genes, Cluster 5 *n* = 474 genes, Cluster 6 *n* = 95 genes, Cluster 7 *n* = 226 genes, Cluster 8 *n* = 106 genes, Cluster 9 *n* = 103 genes, Cluster 10 *n* = 2182 genes, Cluster 11 *n* = 563 genes, Cluster 12 *n* = 536 genes, Cluster 13 *n* = 93 genes, Cluster 14 *n* = 341 genes, Cluster 15 *n* = 219 genes, Cluster 16 *n* = 1176 genes, Cluster 17 *n* = 338 genes, Cluster 18 *n* = 37 genes, Cluster 19 *n* = 45 genes, Cluster 20 *n* = 1319 genes, Cluster 21 *n* = 801 genes, Cluster 22 *n* = 44 genes, Cluster 23 *n* = 95 genes, Cluster 24 *n* = 283 genes, Cluster 25 *n* = 138 genes, Cluster 26 *n* = 30 genes, Cluster 27 *n* = 68 genes, Cluster 28 *n* = 200 genes, Cluster 29 *n* = 56 genes, Cluster 30 *n* = 236 genes, Cluster 31 *n* = 90 genes, Cluster 32 *n* = 256 genes, Cluster 33 *n* = 1,008 genes, Cluster 34 *n* = 3,073 genes, Ubiquitous *n* = 3,000 genes. **c**, Abundance of *Nrsf* mRNA in forebrain. The individual data points are shown as individual bars. **d–f**, Averaged H3K27me3 profiles near promoter regions (*x*-axis) for liver ChIP–seq signals over Cluster 10 genes (**d**), forebrain ChIP–seq signals over Cluster 34 genes (**e**) and forebrain ChIP–seq signals over Rest-targeted genes in Cluster 34 (**f**).

**Extended Data Fig. 10:**
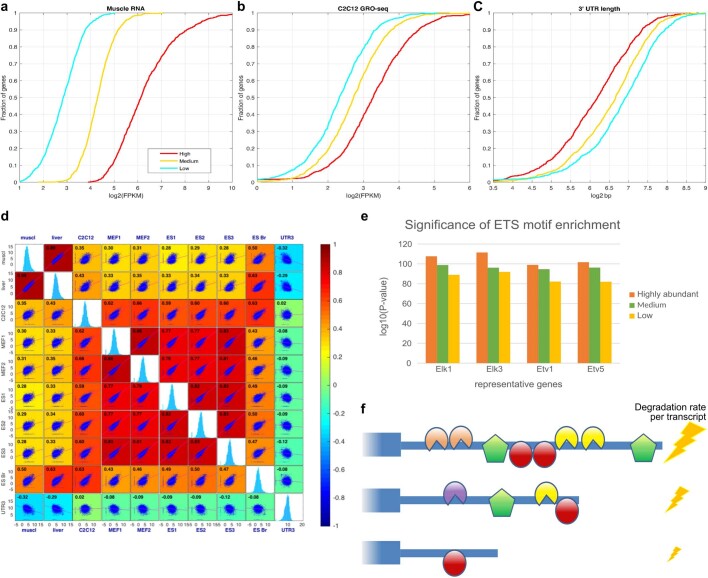
Regulatory mechanisms of ubiquitous genes. **a–c**, Cumulative distribution function plots of polyA RNA-seq measurements from skeletal muscle (**a**), C2C12 GRO-seq data (**b**) and average 3′UTR length (**c**) are compared among three equal-sized groups of ubiquitous genes defined by their RNA-seq abundance. **d**, Comparisons of 3′UTR length, GRO-seq, Bru-seq and polyA RNA-seq assays among multiple different samples. Pearson correlation scores between each pair of measurements on the columns and rows are visualized using a heat map. In the corresponding cell of the comparison, a scatter plot is provided. On the diagonal are histograms of each individual measurement. *n* = 24,832 detectable genes. **e**, Significance of ETS motif enrichment in the promoters of ubiquitous genes determined using AME in MEME suite. *n* = 1,000 each for high, medium and low groups. **f**, A model is proposed that longer 3′UTR may harbour more binding sites for RNA-decay apparatus, leading to lower abundance at steady states.

Canonical correlation analysis (CCA) was performed on the top 20 PCs and Boolean variables for tissue identities, stages, gender and dissection metadata. Standardized canonical variables scores were visualized using the heat map in Extended Data Fig. 6c, while *z*-scores of sample canonical coefficients were visualized using the heat map in Extended Data Fig. 6b, d. Canonical-correlation gene loading coefficients were calculated by multiplying the PC-gene loading coefficient matrix (from PCA) and canonical-correlation PC loading coefficients (from CCA). Genes with the highest positive values and lowest negative values were used to interpret biological meanings for each CC (Supplementary Data 3).

The dynamic genes were defined as those with at least tenfold difference in FPKM values between the most and least abundant RNA samples; genes with less than tenfold difference were defined as flat, or ubiquitous. Dynamic genes and ubiquitous genes were categorized into different classes (protein-coding etc.) on the basis of gene types annotated by GENCODE M4. One-way and two-way hierarchical clustering were done using Pearson correlation coefficient and average linkage for the dynamic genes. Clusters were defined by traversing from the root of the tree towards the leaves, and splitting out clades with different dominant tissues and GO terms, recognized manually, until no more major clusters could be split out. Clades with at least 30 nodes were defined as major clusters. In order to test the robustness of the results, we did an independent analysis with the forebrain, hindbrain and neural tube removed to decrease CNS representation, using the same methodology. Another independent analysis was performed using TPM values for all the tissues, using the same methodology. The main conclusions were largely the same.

### Whole-tissue RNA-seq transcription factor analysis

TF expression vectors were used to generate *t*-SNE and clustering maps using the same settings as the whole-transcriptome analysis. Transcription factor families were compared against cluster identities. The hypergeometric test was performed to assess enrichment.

### Embryo sex inference

For the samples that were made from single embryos, we inferred their sex by comparing gene expression levels of *Xist* (a female marker) and *Ddx3y* (a male marker). Embryos that expressed *Xist* only are female while those that express *Ddx3y* only are male. Mixed embryo pools had both genes detected.

### Ubiquitous gene analysis

Among the genes defined ubiquitous by the whole-tissue RNA-seq analysis, those with log_2_(FPKM + 0.1) values no higher than 2 were removed. The 3,000 genes with smallest sample variance were equally assigned into high, medium and low groups on the basis of their average FPKM values.

GRO-seq and Bru-seq reads were mapped and quantified using the ENCODE standard pipeline for computational consistency. Average 3′ UTR lengths for each gene were extracted from the GENCODE M4 annotation. The log_2_(FPKM + 0.1) values and log_2_(3′ UTR length) were used for comparisons and linear regressions.

### Histone modification analysis

Histone modification ChIP–seq data were processed using the ENCODE ChIP–seq pipeline (https://www.encodeproject.org/pipelines/ENCPL220NBH/), and log_2_ fold change for ChIP–seq samples over input controls were calculated and plotted using deepTools2.4.1 (https://github.com/fidelram/deepTools/tree/2.4.1). To summarize the fold decrease in histone modification signals in a specific sample among a specific cluster of genes, a 4-kb window enclosing the TSS at the centre was used and average log_2_ fold changes against input samples were calculated and visualized using a 3D heated barplot. The fold decrease was the difference between the fold changes of the earliest and latest time point. Rest target overlap *P* value was calculated based on the hypergeometric test using the iQNP Rest ChIP–seq target list published previously^24^.

### Gene ontology analysis

FuncAssociate 3.0 (http://llama.mshri.on.ca/funcassociate/) was used at its default settings for term calling.

### C1 scRNA-seq clustering and *t*-SNE visualization

Spike and tRNA gene FPKM values were removed to rescale FPKM values. Libraries with no cells or more than one cell in their corresponding C1 chambers spotted by microscope were removed. Libraries from the same C1 Fluidigm chip that had systemic 3′ coverage bias were all removed. Cells with fewer than 100,000 reads mapped to the transcriptome or fewer than 4,000 genes above 10 FPKM cutoff were removed. Genes that were expressed in fewer than 5 cells (0.5%), or at lower than 10 FPKM in all cells, or that were covered by fewer than 100 mapped reads in all cells were filtered out. We then used log_2_-transformed FPKM + 1 pseudo-count values for the following analyses. The genes were ranked based on their dispersion scores (defined by sample variance over sample mean). The top 1,500 genes were selected, from which non-coding genes and mitochondria genes were filtered out, leaving 1,269 genes. *t*-SNE projection was done based on these genes, using the top 30 PCs and 30 as perplexity parameter (default for Laurens van der Maaten’s original MATLAB script)^56^. Two-way hierarchical clustering was then performed on the log_2_-transformed FPKM values using complete linkage with Spearman rank correlation coefficient to cluster the cells. Cell types were annotated manually.

### 10x scRNA-seq clustering and *t*-SNE visualization

UMI counts from CellRanger were filtered first, where cells with fewer than 1,000 genes detected and genes detected in less than 0.1% of cells were removed. Within each cell, counts were divided by the sum and multiplied by 10,000, added to 1, and log-transformed. The top 4,000 high-dispersion genes were identified. To remove noise (https://github.com/brianpenghe/python-genomics), we first performed hierarchical clustering for these genes and then extracted genes that fell in ‘tight’ clusters (those with more than two members after cutting the dendrogram at 0.8 distance), removing a large number of sporadic genes which had high dispersion scores but were barely co-expressed with other genes. These genes were used in place of ‘highly-variable genes’ for the Seurat pipeline. Using the Seurat pipeline, cells with more than 20% mitochondria reads or more than 8,000 genes detected were removed. Genes were regressed against the number of UMIs per cell and mitochondria percentage and scaled. The resulting matrix, guided by the aforementioned feature genes, was used to perform PCA. Jackstraw was then performed using Seurat’s default settings, resulting in 42 significant PCs. These PCs were in turn used for Louvain cell clustering and *t*-SNE visualization. Clusters 3, 4, 5, 6, 8, 12 and 13 were further re-clustered using the same method, yielding clusters 17–24.

### Marker gene identification for C1 and 10x scRNA-seq data

Marker genes (Supplementary Data 4) were calculated using Seurat’s FindMarkers() for both C1 and 10x single-cell data with min.pct = 0.25 and its default Wilcoxon rank sum test with min.diff.pct set to be 0.2 or 0.4. For marker visualization, each cell type was down-sampled to at most 100 cells for 10x data and at most 30 cells for C1 data. Min.diff.pct was set to be 0.2 and the top 15 markers for each cell type were visualized.

### Comparing C1 and 10x cell types

Two methods were used to compare cell type annotations for C1 and 10x data. On the basis of Seurat3’s ‘Label transfer’ method, transfer anchors were calculated from 10x data and were used to predict cell types for C1 data. Independently, the scaled 10x data matrix was used to train a multinomial logistic regression model using scikit-learn package. The trained model was used to predict cell types for C1 data.

### Integrating C1 and 10x data for UMAP visualization

Seurat3 was used to calculate integration anchors and to integrate the two different types of datasets. The joint set was scaled and visualized on UMAP based on an arbitrary top 50 PCs.

### Lineage trajectory analyses

Prior to lineage inference, doublets were removed using a Scrublet-based^57,58^ subclustering scheme. Monocle3 alpha (2.99.3) was then used for trajectory analysis of the 10x data that contain a large number of cells. The function plot_pc_variance_explained() was used to select significant PCs above the knee cutoff. UMAP visualization and SimplePPT method were applied. The root node for each lineage tree was defined as the node that connects to the largest number of the cells from the earliest developmental time point (E10.5).

### Differential transcription factor analysis

Transcription factors recorded at TFDB (http://bioinfo.life.hust.edu.cn/AnimalTFDB/) were selected from marker genes derived at 0.2 cutoff (described above), to infer evidence-based interaction networks using STRING^59^ (https://string-db.org/). A Python interface for STRING was used to query the database directly and render the resulting graph using Graphviz^60^. Edges of type ‘database’ and ‘experimental’ were used, filtered to meet a confidence value of greater than 0.400. Nodes were coloured using normalized values obtained from Scanpy^61^. The graph was laid out using layout software included with the Graphviz package. The algorithm used was SFDP. The complete code base as well as Docker and Singularity container recipes can be accessed on the GitHub repository: https://github.com/hamrhein/mouse_embryo.

### IDEAS states

The IDEAS epigenetic states on the ENCODE3 mouse developmental data were generated by the IDEAS software^13,14^ using ten epigenetic marks: H3K27ac, H3K27me3, H3K36me3, H3K4me1, H3K4me2, H3K4me3, H3K9ac, H3K9me3, ATAC–seq and DNase methylation data. We first converted the raw data in each sample to –log_10_*P* values using a negative binomial model. The mean and variance parameters of the model for each sample were calculated using the bottom 99% of the data. We then adjusted the mean parameters at each genomic position from the input data to account for local genomic variations. Specifically, we downloaded the input data for each tissue (see list of datasets), and we calculated rolling means per genomic position using a 20-kb window centred at the position, for both signals and the input. The ratio between the two means at each position was multiplied to the overall mean estimate of the sample, and we normalized the ratios across the genome to have mean 1. We treated the –log_10_(*P* value) as input data for IDEAS, capped at 16, and we ran the program in its default setting. The output from IDEAS is a set of genome tracks to display in the genome browser, where each epigenetic state is assigned a colour as a weighted mixture of colours pre-assigned by the program to each epigenetic mark. The IDEAS segmentation can be accessed by the Hub link at http://woldlab.caltech.edu/ENCODE3_Mouse_RNA_paper_yuzhang_me66n/.

### Cell type and lineage-specific marker gene identification and cCRE assignment

Genes exclusively expressed in only one cell type or lineage were regarded as ‘marker genes’ for this series of analyses. Using the high-resolution C1 Fluidigm data, marker genes at 0.2 or 0.4 cutoff were cross-intersected to derive exclusively expressed markers of cell types or groups of related cell types (Muscle 1 + Muscle 2, Muscle 2 + Muscle 3, Muscle1–3, Chondrocyte + Perichondrium, EMP + Macrophage etc.). Candidate *cis*-regulatory elements (cCREs) were defined by merging all the DHS peaks called by the ENCODE HOTSPOT2 pipeline. These merged regions were assigned to closest transcription start sites of genes that are expressed (FPKM higher than 0.1 in at least one bulk limb tissue, or detected in more than four cells in single-cell limb data). These merged regions were then compared against IDEAS chromatin states generated from ENCODE3 mouse developmental time course data (see below). Only the peaks that overlapped with active (state 14, 19, 20, 21, 23, 24, 25, 27, 28, 30–32), poised (8 and 13) or bivalent (26 and 29) IDEAS states were regarded as ‘IDEAS active DHS’ (cCREs). Finally, these cCREs assigned to the aforementioned marker genes’ TSSs were regarded as cell-type or lineage-specific cCREs. On the basis of the distance between each cCRE and its assigned gene, cCREs were further divided into three categories: proximal (the distance is no greater than 200 bp in any direction), middle (the distance is longer than 200 bp and no greater than 2,000 bp in any direction) and distal (the distance is longer than 2,000 bp in any direction).

### Motif analysis

**Extended Data Fig. 11:**
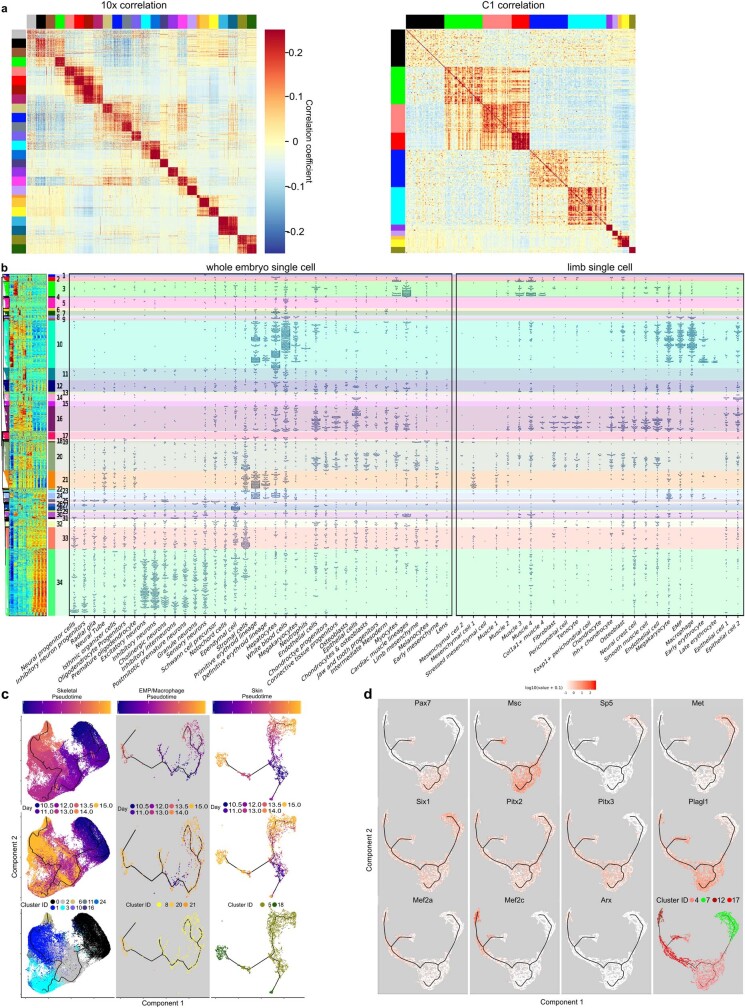
Cell-type relationships inferred from single-cell data I. **a**, Cell–cell correlations. Feature genes were used to calculate and visualize Pearson correlation coefficients between cells. Specific cell-type populations (indicated by colour bands on the axes) were downsampled to 100 (10x) or 30 (C1). **b**, Comparing whole-embryo single-cell data with limb single-cell data. As an extended version of Fig. 1d, this comparison added a panel for 10x single-cell RNA-seq limb data (far right). **c**, Lineage inference. Skeletal (left), myeloid (middle) and skin (right) cell types were used for lineage inference, respectively. Pseudotime, developmental time and cell type are presented from top to the bottom. **d**, Selected transcription factor expressions are displayed on the Monocle graphs produced from the 10x data for the four cell types comprising the myogenic lineage.

**Extended Data Fig. 12:**
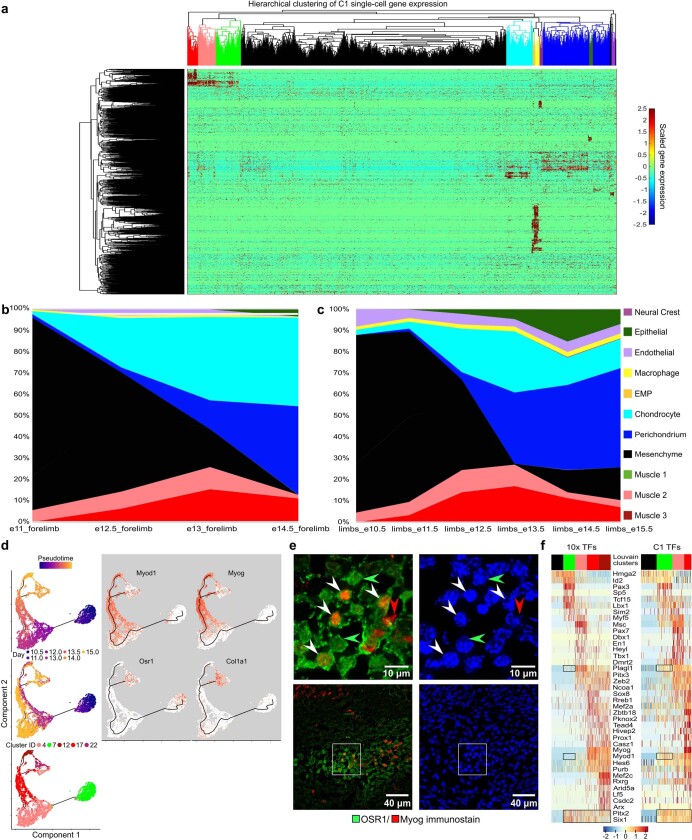
Cell-type relationships inferred from single-cell data II. **a**, Feature gene expression profiles of C1 single cells. Normalized log-transformed FPKM values (*y*-axis) are used for hierarchical clustering using Spearman coefficients with complete linkage. Major cell types (*x*-axis) together with an Lmo2^+^ mesenchyme subtype are highlighted using colours corresponding to Fig. 3a. The overall picture showed different numbers of marker genes across cell types. **b**, **c** CIBERSORT deconvolution of bulk data. CIBERSORT was used to deduce proportions of major cell types (*y*-axis) present in staged samples (*x*-axis) of independently produced forelimbs (**b**) and ENCODE mixed limb materials (**c**). The colour codes match Fig. 3a. **d**, Monocle lineage inference for four skeletal muscle clusters including cluster 22. Pseudotime, developmental time and cell type are shown on the left, and marker gene expression is mapped on the right. **e**, 20-micrometre sections of mouse E13.5 forelimb double-immunostained for Osr1 (green) and Myog (red) (left), with a DAPI (blue) counterstain (right). All images taken with 63X oil immersion objective. Images in upper panels are enlarged from boxed areas in lower panels. Arrowheads: green: Osr1(+) Myog(−) nucleus. Red: Myog(+) Osr1(−) nucleus. White: double (+/+) cells. Immunocytochemistry was repeated three times independently. **f**, TFs enriched in skeletal muscle cell types and mesenchyme in either 10x or C1 data. Cells were down-sampled for display (similar to Supplementary Fig. 3); cell types are colour-coded for cell cluster identity. Outlines highlight genes (Myod1;* Plagl1*) with early stage low-level expression detected in C1 but not 10x data versus pan-lineage markers (*Six1*; *Pitx2*) detected in both.

**Extended Data Fig. 13:**
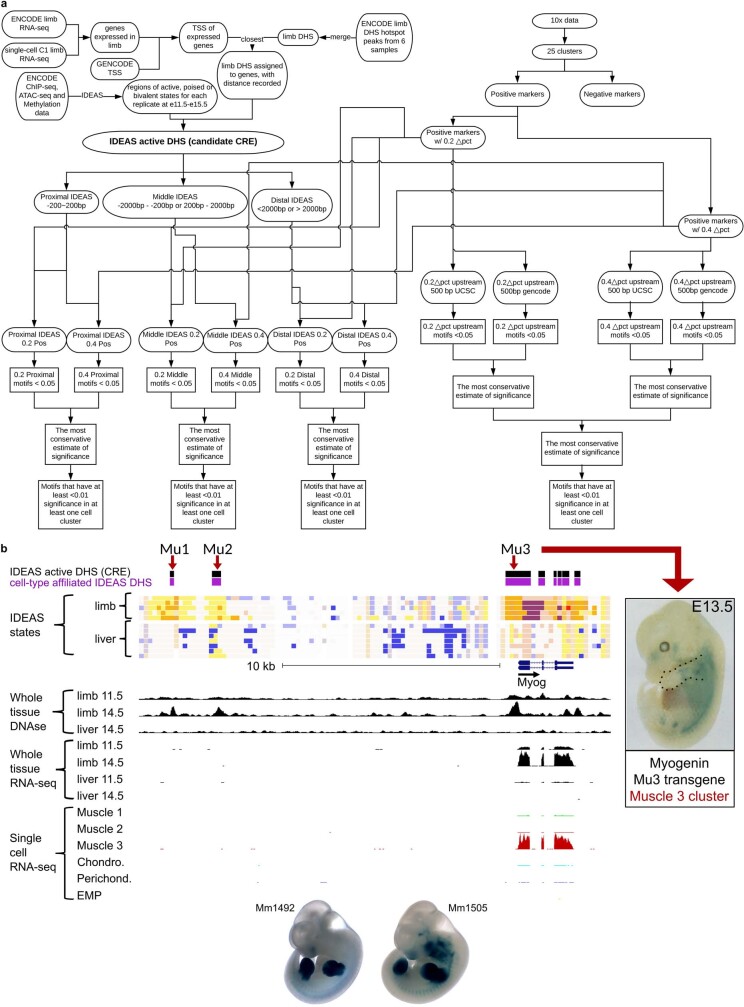
Analysis of CREs using ENCODE chromatin data and single-cell RNA-seq data I. **a**, A flowchart of the analysis. **b**, Computationally predicted regulatory elements at the Myog locus. From the top to the bottom are the tracks for limb IDEAS active DHS (black bars), cell-type affiliated ones among the former (purple bars), IDEAS scores of limb and liver samples from early to late time points, bulk DNase-seq raw data, bulk RNA-seq raw data, and aggregated C1 single-cell RNA-seq data per cell type. Validation of the Mu3 element in mouse embryo by enhancer assay is also included at the bottom right. (Modified, with permission, from Yee and Rigby 1993, © Cold Spring Harbour Laboratory Press^64^.) Bottom, examples of limb-positive enhancer results from the VISTA database that are not cell-type-specific.

**Extended Data Fig. 14:**
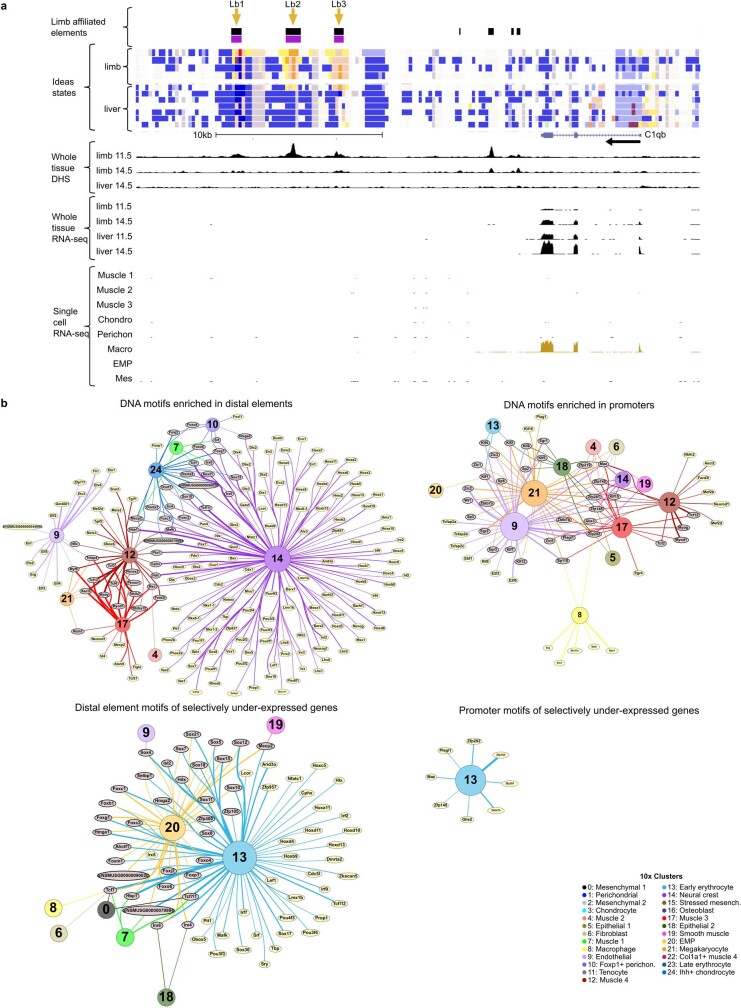
Analysis of CREs using ENCODE chromatin data and single-cell RNA-seq data II. **a**, UCSC genome browser visualization of the *C1qb* locus, which is in the limb macrophage cluster. Three candidate enhancers for limb-specific expression of this macrophage gene were identified (Lb1–Lb3). **b**, Enriched motifs over regulatory elements. Motifs enriched at the distal elements or promoters of positive and negative markers for each cell type found in the 10x data (see Methods) are visualized using a similar method as Fig. 2b. Colours and numbers of the round nodes correspond to 10x cell type identities (legend at bottom right), and the grey and yellow ovals represent shared and unique motifs, respectively.

**Extended Data Fig. 15:**
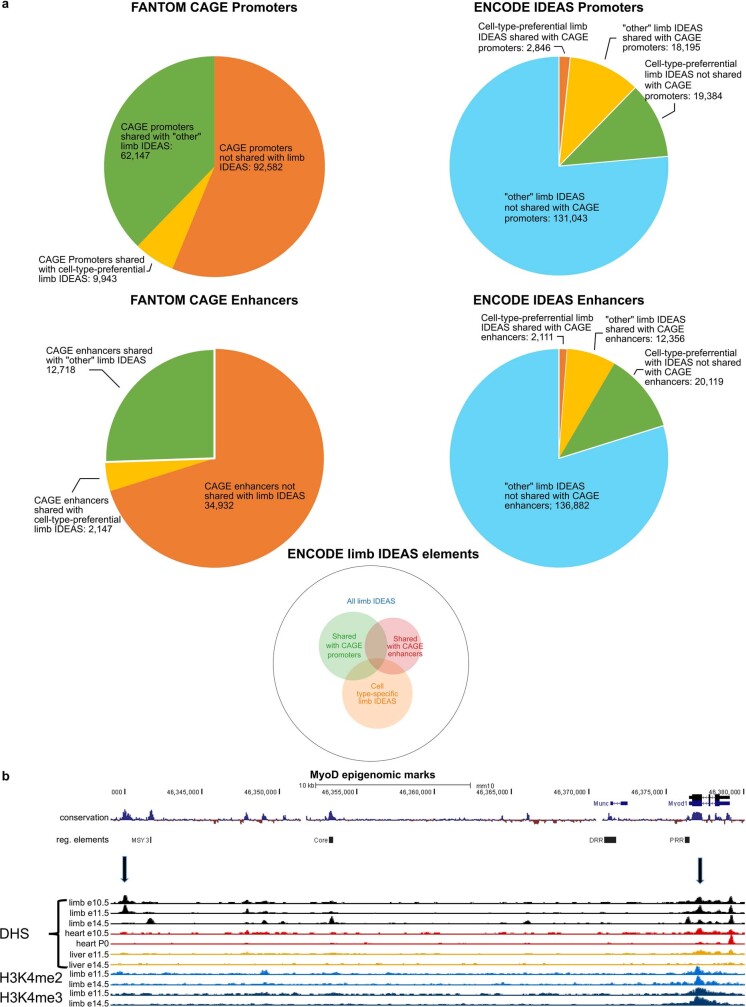
Analysis of CREs using ENCODE chromatin data and single-cell RNA-seq data III. **a**, Comparison between FANTOM5 detected promoters and enhancers with promoters and enhancers detected in this study. Elements labelled as “other” are either active or poised in limb generally, but are not cell-type preferential. **b**, UCSC Genome Browser shot at the MyoD1 locus. DHS locus accessibility data are shown, along with H3K4me2 and H3K4me3 histone ChIP–seq data. Vertical arrows indicate regions of early chromatin accessibility (see text and Fig. 4a).

For whole-tissue RNA-seq promoter motif analysis, the upstream 500 bp sequences of each co-expression cluster were extracted and pooled. For limb cell type-associated gene promoter analysis, the upstream 500 bp sequences of each cell type’s marker genes (derived from 10x data using Seurat, min.diff.pct = 0.4) were extracted and pooled. For limb cell type-associated cCRE analysis, the DNA sequences of proximal, middle, or distal cCREs for each cell type’s marker genes were extracted and pooled. These sequence pools were used for motif discovery. A detailed flowchart can be found in Extended Data Fig. 11.

The analysis of transcription factor recognition motifs was carried out using version 4.11.2 of the MEME-SUITE^62^. Motifs annotated in the CIS-BP database^55^ (http://cisbp.ccbr.utoronto.ca/) were used to evaluate motif enrichment in the sequence pools mentioned above; enrichment was scored by the AME program in the MEME-SUITE^63^. The analysis was carried out twice based on UCSC mm10 refFlat and GENCODE M4 separately and only motifs with corrected *P* values smaller than 0.01 in both analyses were called significant.

### Comparing whole-tissue RNA-seq and single-cell RNA-seq

10x single-cell data (without log transformation or Gaussian scaling) and the aforementioned 10x feature genes were used as input for CIBERSORT^27^ (https://cibersort.stanford.edu/) to compare against whole-limb RNA-seq data (without log transformation or Gaussian scaling). To compare cell type-associated gene signatures against ENCODE whole-tissue RNA-seq clusters, cell type-associated marker genes were acquired ref. ^11^ (Table S4 for gene names and Table S3 for cell type names from ref. ^11^) and filtered (p_val <0.05 and q_val <0.05). Noting that CIBERSORT is highly sensitive to the choice of input gene set, these signature genes were mapped to the ordered heat map of the bulk-tissue clustergram (Fig. 1d). For better visualization, we jittered individual dots, to create a re-purposed swarm plot to show distribution of the locations (instead of quantities) of signature genes for each cell.

### Immunocytochemical detection in tissue sections

Staged embryos were fixed in 4% PFA in PBS, cryoprotected with 30% sucrose in PBS, and frozen in OCT on dry ice. Ten-micrometre cryosections were blocked using the mouse on mouse blocking reagent from Vector (cat. # MKB-2213), and then stained with antibodies for OSR1 (mouse monoclonal Santa Cruz cat. # 376545 at 1:40) and myogenin (Abcam RabMab cat. # ab124800 at 1:40). Secondary detection was done with InVitrogen donkey anti-rabbit Alexa 594 cat. # A21207, and InVitrogen goat anti-mouse Alexa 488 cat. # A11029, both at 1:300 dilutions. Sections were first screened on a Zeiss Axio Observer Z.1 and then imaged for deconvolution microscopy using a Leica DMI6000, with a 63× oil immersion lens, and Huygens Professional deconvolution software from SVI.

### Reporting summary

Further information on research design is available in the Nature Research Reporting Summary linked to this paper.

## Online content

Any methods, additional references, Nature Research reporting summaries, source data, extended data, supplementary information, acknowledgements, peer review information; details of author contributions and competing interests; and statements of data and code availability are available at 10.1038/s41586-020-2536-x.

## Supplementary information

Supplementary Information This file contains Supplementary Notes 1-3, which include Supplementary Figures 1-7, Supplementary Tables 1-2 and Supplementary References.

Reporting Summary

Supplementary Data 1 Bulk RNA-Seq gene expression clusters.

Supplementary Data 2 PCA loading and Jackstraw analysis.

Supplementary Data 3 CCA metadata matrix and loading variables.

Supplementary Data 4 Cell-type markers and cluster-specific TFs.

Supplementary Data 5 Motifs from promoters from bulk and single-cells and motifs from enhancers in single-cells.

Supplementary Data 6 IDEAS elements and URLs for browser tracks.

Video 1 3D projection of bulk transcriptome PCA.

## Data Availability

These data are part of the ENCODE Consortium mouse embryo project, which provides companion microRNA-seq, DNA methylation, histone mark ChIP–seq, and chromatin accessibility datasets for the sample matrix (https://www.encodeproject.org/matrix/?type=Experiment&status=released&perturbed=false&lab.title=Barbara+Wold%2C+Caltech&award.rfa=ENCODE4). The raw and first level processed data can be accessed at the ENCODE portal (https://www.encodeproject.org) with the following experiment accession numbers: bulk RNA-seq: ENCSR574CRQ; Fluidigm C1 SMART-seq: ENCSR226XLF; 10x Genomics (raw data only): ENCSR713GIS. For convenient viewing on the UCSC single-cell browser (https://mouse-limb.cells.ucsc.edu/), we have uploaded the AnnData matrices corresponding to ENCSR226XLF (Fluidigm C1 SMART-Seq) and ENCSR713GIS (10x Genomics). The processed data matrix for the Fluidigm C1 is available at https://cells.ucsc.edu/mouse-limb/C1_200325/200315_C1_categorical.h5ad and the 10x Genomics processed matrix is available at https://cells.ucsc.edu/mouse-limb/10x/200120_10x.h5ad.

## Code availability

Standard ENCODE RNA-seq pipeline: https://www.encodeproject.org/pipelines/ENCPL002LSE/; ENCODE ChIP–seq pipeline: https://www.encodeproject.org/pipelines/ENCPL220NBH/; all MATLAB scripts: https://github.com/brianpenghe/Matlab-genomics. 10x single-cell RNA-seq data were processed using CellRanger with a compatible GTF annotation and default parameters. deepTools2.4.1: https://github.com/fidelram/deepTools/tree/2.4.1; FuncAssociate 3.0: http://llama.mshri.on.ca/funcassociate/; TFDB: http://bioinfo.life.hust.edu.cn/AnimalTFDB/; motifs annotated in the CIS-BP database: http://cisbp.ccbr.utoronto.ca/; STRING: https://string-db.org/. The complete code base for promoter motif graphs, STRING interaction graphs, as well as Docker and Singularity container recipes can be accessed on the GitHub repository: https://github.com/hamrhein/mouse_embryo. The IDEAS segmentation can be accessed by the Hub link at http://woldlab.caltech.edu/ENCODE3_Mouse_RNA_paper_yuzhang_me66n/. CIBERSORT: https://cibersort.stanford.edu/.
