# Supplementary material for: The changing mouse embryo transcriptome at whole tissue and single-cell resolution
Source: Nature. 2020 Jul 29;583(7818):760–7. doi: 10.1038/s41586-020-2536-x (PMC7410830; doi:10.1038/s41586-020-2536-x)
Supplement: Supplementary file 1 — This file contains Supplementary Notes 1-3, which include Supplementary Figures 1-7, Supplementary Tables 1-2 and Supplementary References. [file 41586_2020_2536_MOESM1_ESM.docx]

**Supplementary Note 1. Individual cluster analysis from hierarchical clustering of bulk RNA-seq data.**


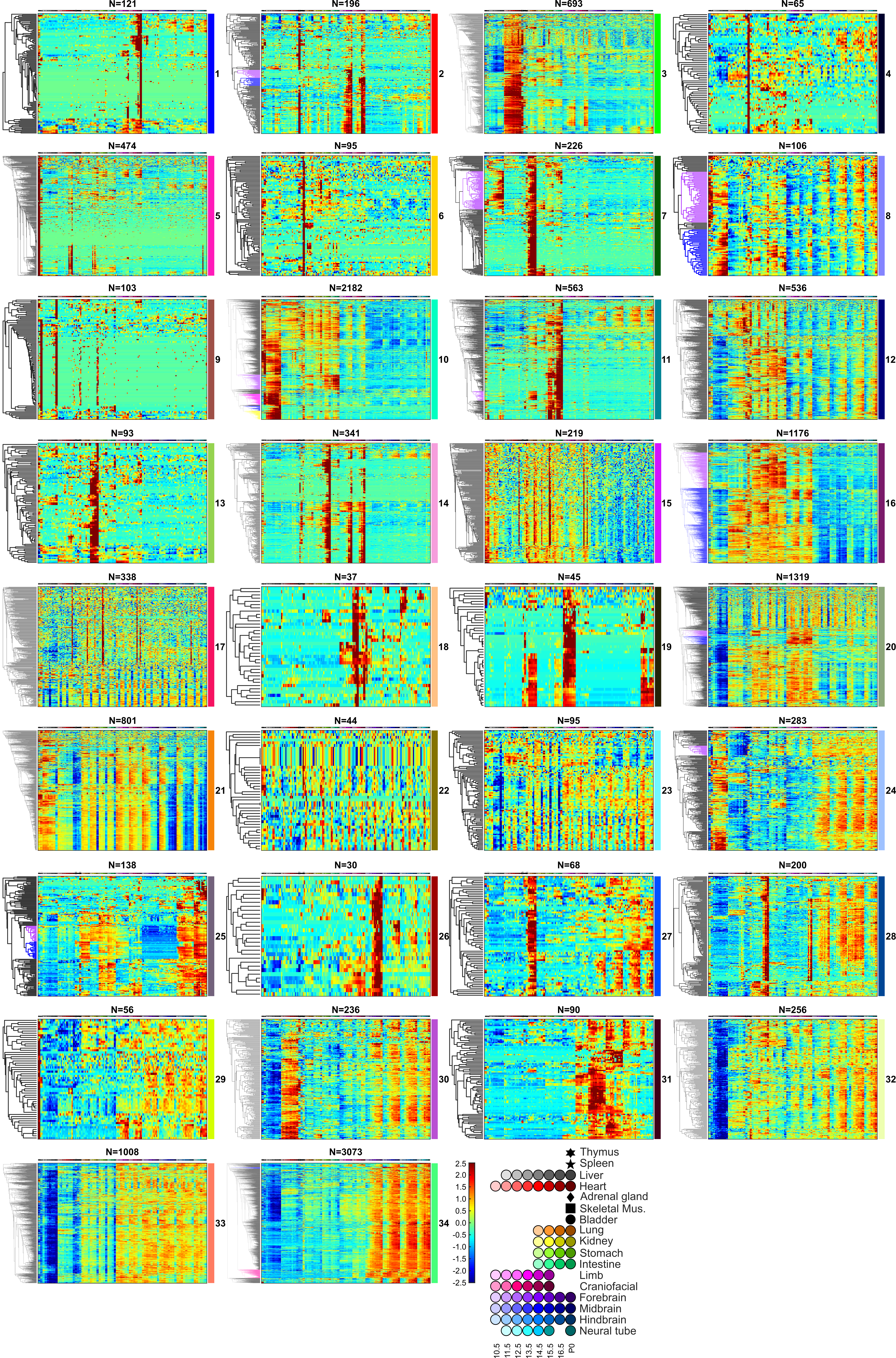


**Supplementary Figure 1.**

The tissue source color code bar and gene number (N) are shown at the top of each panel. The cluster identity number and corresponding color code from Fig.1 are shown on the right, and the hierarchical tree structure is displayed to the left of each cluster. Normalized RNA levels were mapped to the heatmap scale shown (bottom right). Further analysis of each cluster is given in the following legends to clusters 1 to 34.

**Cluster 1** from hierarchical clustering analysis of bulk RNA samples.

1. Cluster 1 has prominent increasing expression in limb and craniofacial prominence.
2. Over one third of the genes in this cluster are genes coding for keratin and keratin associated proteins. Top GO terms include “intermediate filament” (p=3.7e-34) and “hair cycle” (p=2.4e-7), pointing to development of skin and hair.

**Cluster 2** from hierarchical clustering analysis of bulk RNA samples.

1. Cluster 2 has prominent expression in skeletal muscle and an increasing trajectory in limb and craniofacial prominence.
2. Cluster 2 contains multiple muscle regulators like Myod1 and Myog. Its top GO terms include “muscle system process” (p=4.5e-18) and “contractile fiber part” (p=9.9e-14). The increasing expression in limb and craniofacial prominence is likely due to differentiation of muscle precursors and to increasing relative muscle mass as a fraction of the total tissue.
3. In addition to the dominant muscle-limb-face feature, there are two clades with different patterns that illustrate the informational leverage that comes from a more pure P0 dissected tissue (here muscle).
   1. The clade of 13 genes labeled in blue have increasing expression in limb and craniofacial prominence but not in the P0 pure skeletal muscle sample. Among the 13 genes, five (Dcstamp, Mmp13, Bglap, Ifitm5 and Ibsp) are associated in prior work with osteogenesis.
   2. The clade of 13 other genes labeled in purple is biased for limb alone, and not cranioface. It includes four major urinary protein (MUP) genes at low but detectable abundance. The mouse genome has 21 annotated Mup genes in a 2Mb cluster on Chromosome 4. Although none have human orthologs, members of the family have known functions in mouse chemical communication and nutrient metabolism. A recent study reported dramatic and unexpected upregulation of Mup1 in mouse embryos when Sox2, a transcription factor regulating proximal bone formation in limbs, is mutated. This raises the possibility that the MUPs in this limb cluster play a role in limb development.
4. ** Technical user note: Sporadic samples of adrenal gland, kidney, lung, stomach, hindbrain and neural tube from this mouse embryo bulk RNA ENCODE series show slight enrichments for genes from this cluster, implying variable minor tissue contamination during dissection.

**Cluster 3** from hierarchical clustering analysis of bulk RNA samples.

1. Most genes in this cluster have high and constant levels of expression in heart, and roughly half also have substantial expression in skeletal muscle-containing samples, which is expected due to contractile protein genes shared in both kinds of striated muscle.
2. GO terms are mainly about muscle, including “contractile fiber part” (p=8.9e-47) and “regulation of heart contraction” (p=4.3e-21)
3. *The clade in the upper half of the heatmap has narrow dark red bars, that indicate single replicate enrichment. This group of genes contains mostly pseudogenes (see also Cluster 15).

**Cluster 4** from hierarchical clustering analysis of bulk RNA samples.

1. Genes in Cluster 4 show differing degrees of bladder-specific expression, which may result from a bladder-specific cell type that has a unique transcriptome signature.
2. GO analysis produced no terms. Mouse bladder has not been extensively studied, and under-annotation may compromise the statistical power of GO in this case.

**Cluster 5** from hierarchical clustering analysis of bulk RNA samples.

1. Genes in Cluster 5 are very prominently expressed in thymus and most have minimal expression in other tissues.
2. Highly expressed genes also have positive signals in several non-thymus samples, with atypical irreproducibility between replicates. A candidate explanation is batch-specific contamination of thymus-proximate tissues with thymus during dissection. While this kind of contamination doesn’t greatly alter global QC scores, it is readily detectable in this clustering analysis (see also CCA analysis).
3. GO analysis revealed enrichment in later stage maturing immune components, especially T-cell terms. Top terms include “immune system process” (p=1.8e-18) and “regulation of T-cell activation” (p=3.0e-13).
4. Roughly one quarter of the genes are T-cell receptor components (alpha chain, gamma chain and delta chain). Interestingly, the two recombinases Rag1 and Rag2 are also in this cluster, indicating a TCR VDJ theme for this cluster.

**Cluster 6** from hierarchical clustering analysis of bulk RNA samples.

1. The unifying theme of this cluster is high expression in the adrenal gland.
2. Top GO terms include “hormone biosynthetic process” (p=1.5e-7) and “hormone metabolic process” (p=7.3e-7). More specifically, Cyp11b1, Cyp21a1 and Cyp11b2 contribute to the term “mineralocorticoid biosynthetic process” (p=2.8e-7). These cytochrome P450 genes are involved in biosynthesis of aldosterone which, unlike many other hormones, is produced only in the adrenal gland. However, these genes also have detectable expression signals in E15.5 and E16.5 samples of kidney. Their presence at E15.5 and E16.5 stages and absence in E14.5 and P0 may be due to contamination in E15.5 and E16.5 pooled samples, while E14.5 and P0 samples from individual embryos were more contamination-free.

**Cluster 7** from hierarchical clustering analysis of bulk RNA samples.

1. The central theme of this cluster is prominent expression in the developing kidney, where the RNA trajectories generally increase over time. Roughly 40% of these genes are also expressed in liver, again with increasing trajectories, plus some smaller subclades that are shared with gut or lung samples.
2. Top GO terms of this cluster include transporter-related categories such as “sodium ion transport” (p=2.0e-14) and “anion transport” (p=4.7e-9) and structural terms like “apical plasma membrane”. This cluster is dominated by genes responsible for transporter machinery and epithelial cell organization in the kidney. This cluster was also highlighted by CCA analysis. Further examination found that the increasing number of nephrons through time, and the up-regulation, in particular, of genes of the proximal tubule are explanatory.
3. The clade of 72 genes labeled in purple contains genes enriched in both liver and kidney. The top enriched GO terms for this group are for amino acid catabolic processes performed in both liver and kidney (“organic acid metabolic process” (p=7.7e-12), “fatty acid metabolic process” (p=1.2e-7) and “alpha-amino acid catabolic process” (p=1.5e-6). 20 of these genes are enriched in kidney proximal tubule brush border cells while 7 are enriched in hepatocytes^12^.

**Cluster 8** from hierarchical clustering analysis of bulk RNA samples.

1. The genes in Cluster 8 have increasing expression patterns in almost all tissues, although the kinetics of increase differ.
2. Top enriched GO terms include “inflammatory response” (p=1.0e-6) and “extracellular exosome” (p=1.5e-5).
3. There are two major clades. The clade labeled in purple is consistent with genes marking the immune system, whose levels are highest in thymus and spleen, but also include expression in the hematopoietic fetal liver. Subsets of these genes increase at later times in other tissues. GO analysis called terms including “regulation of T cell activation” (p=8.8e-5) and “inflammatory response” (p=2.2e-5). The second major clade, labeled in blue, is dominated by increasing expression in liver and gut tissues. Top GO terms included “extracellular exosome” (p=3.3e-9).

**Cluster 9** from hierarchical clustering analysis of bulk RNA samples.

1. The genes in Cluster 9 have highest enrichment by far in spleen and on the P0 liver, but not at earlier times. Moderate abundance is also seen in adrenal gland and lung. There is minimal but detectable expression in all other tissues at P0 but very little at all times before birth.
2. GO analysis did not yield significantly enriched terms, but more than half of the genes in this cluster are immunoglobulin components (kappa and lambda light chain variables, heavy chain variables and constant regions, consistent with B-cell maturation, appearing in liver, spleen and in lesser proportions in lung and other lymphatic-containing dissections.

**Cluster 10** from hierarchical clustering analysis of bulk RNA samples.

1. Over 60% of the genes in Cluster 10 are preferentially expressed in liver, much lower in CNS tissues and variously detected in other tissues. The RNA abundances mainly increase with time, but with differing kinetics.
2. Top GO terms of Cluster 10 include the immune system, such as “immune system process” (p=4.8e-101) and “regulation of immune system process” (p=2.0e-62). The additional prominence of many genes in the P0 thymus and/or spleen, along with other non-CNS tissues point to the lymphatic system.
3. In addition to the main immune theme, four clades with distinctions emerged. The one containing 267 genes labeled in purple are most enriched in liver, as well as stomach and intestine, increasing over time. Its top GO terms focus on lipids, including “lipid metabolic process” (p=3.8e-13) and “lipid transport” (p=4.1e-11), pointing to metabolic functions shared by hepatocytes and gut tissues.
4. The clade of 200 genes labeled in pink contains genes enriched in spleen and liver only and point to erythropoiesis. Its top GO terms are mainly related to maturing red blood cells, such as “tetrapyrrole biosynthetic process” (p=1.2e-20) and “erythrocyte development” (p=3.9e-10). DNA motif analysis of promoters in this clade revealed a significant enrichment of Tal1:Gata1, a known pair of regulators essential for hematopoiesis.
5. Members of the clade of 91 genes labeled in blue are mainly expressed in late stage liver and are hepatic functions, as well as in the adrenal gland. Top GO terms include “monooxygenase activity” (p=1.1e-31), “steroid hydroxylase activity” (1.7e-20) and “steroid metabolic process” (p=5.3e-9). More than a quarter of these are protein-coding components of cytochrome P450, which are involved in steroid and drug metabolism. Additionally, six sulfotransferase genes are also in this group. Sulfotransferase plays an important role in the metabolism of drugs, hormones and bile acids.
6. Lastly, the clade of 155 genes labeled in yellow show more constant levels through time in liver, with additional expression detected in adrenal gland, kidney, stomach and intestine. Its top GO terms include “blood coagulation” (1.7e-29) and “alpha-amino acid metabolic process” (3.1e-12), with six coagulation factors, six complement factors, fibrinogens and regulators (protein C and serpins) are found in this clade.

**Cluster 11** from hierarchical clustering analysis of bulk RNA samples.

1. The main theme of Cluster 11 is gut development and differentiation. Its genes are most highly expressed in intestine and are also enriched in stomach, with sharing of specific clades with either kidney (purple) or CNS (blue) tissues.. For the stomach, E14.5 and P0 timepoints show lower expression for multiple clades, which likely reflects systematic dissection differences at the boundaries between the two gut tissues.
2. Top GO terms are mainly about intestine structure, including “brush border” (p=2.3e-11) and “brush border membrane” (p=3.1e-9). Interestingly, out of 16 genes contributing to the term “brush border”, 8 are in the small clade of 43 genes labeled in purple. This clade also has prominent increasing expression in kidney, representing a shared program of brush border genes between kidney and intestine. Other terms include “sodium ion transport” (p=1.0e-6), “digestive system process” (p=1.0e-6) and “alpha-amylase activity” (p=1.8e-6). Additionally, several gut hormones or peptides are found in this group such as cholecystokinin, gastrin, vasoactive intestinal polypeptide, ghrelin, glucagon and insulin genes.

**Cluster 12** from hierarchical clustering analysis of bulk RNA samples.

1. Most genes in Cluster 12 are expressed widely and with an increasing trend, except in the liver, where most of the cluster is depleted at all times.
2. The most prominent secondary theme is strong up-regulation at birth in multiple organs.
3. No GO terms were significantly enriched.
4. This cluster, and subclusters within, are candidates for novel DNA sequence motif-derivation or for correlated mir-signatures that could mediate the birth transition pattern and/or the liver suppression pattern.

**Cluster 13** from hierarchical clustering analysis

1. Genes in Cluster 13 are mostly enriched in lung, especially at later stages.
2. Partly because of the small cluster size, Gene Ontology didn’t provide highly significant terms. However, 4 surfactant-associated proteins contributing to the term “multivesicular body” (p=4.3e-6) are included in this cluster, indicating a possible link to Type II alveolar cells in the lung.

**Cluster 14** from hierarchical clustering analysis.

1. Cluster 14 contains genes that are highly expressed in stomach. Most are also highly expressed in limb and craniofacial prominence at very late stages. About a quarter of them are also expressed in the P0 bladder.
2. The top GO terms are “cornified envelope” (p=6.7e-26) , “keratinization” (p=1.7e-27), “epidermis development” and “keratinocyte differentiation” (p=1.6e-16). The cornified envelope is composed of a layer of dead cells found in skin epidermis and forestomach for protection against the environment. Its major components include loricrin, filaggrin, Involucrin, keratins and small proline-rich protein (SPR) genes that are all found in this cluster, together with the genes required for generating the cornified envelope, such as transglutaminase, cystatin and envoplakin.

**Cluster 15** from hierarchical clustering analysis of bulk RNA samples.

1. Genes in this group are coherently enriched in specific replicate samples, but they do not reproduce between replicates or among related tissues.
2. Almost all genes in this cluster are annotated as known pseudogenes or protein-coding genes with low mappability. These low-mappability genes’ top-abundance mappable counterparts (their corresponding protein-coding genes or paralogs) do not display similar variation (data not shown).

**Cluster 16** from hierarchical clustering analysis of bulk RNA samples.

1. The broad theme of this cluster is expression in most tissues and organs, with the exception of the CNS and liver, both of which show little expression.
2. Over the developmental time course, most members increase in limb and craniofacial prominence but are relatively less changing or decreasing in other tissues.
3. The top GO terms of Cluster 16 are dominated by extracellular matrix (ECM) components, such as “extracellular matrix” (p=8.7e-58), “extracellular region part” (p=6.0e-42) and ‘’basement membrane” (p=4.4e-34). Other significant terms include “regulation of cell migration” (p=8.0e-26), “angiogenesis” (p=1.1e-25) and “cell junction” (p=2.3e-18).
4. This cluster contains two major clades, highlighted in purple and blue, that share expression in bladder, kidney, lung, stomach, intestine, limb and craniofacial prominence. The blue clade is distinct in also showing strong expression in heart. Their GO terms identify different biases. The purple clade features “occluding junction” (p=7.6e-9) in addition to ECM terms, while “angiogenesis” is absent. The blue clade includes most cluster 16 thematic terms, but also emphasizes “anchoring junction” (p=7.3e-19) and particularly “adherens junction” (p=3.0e-18), consistent with epithelial/endothelial cell junction formation and tube morphogenesis. Thus the purple clade focuses on tight junctions that consist of an epithelial barrier and molecular gate between a cell mass and the environment, while the blue clade concentrates on angiogenesis and adherens junctions that link cells together and also carry cadherin receptors important for tissue morphogenesis.

**Cluster 17** from hierarchical clustering analysis of bulk RNA samples.

1. This cluster is divided into two major clades. The upper clade, similar to cluster 15, is coherently enriched in a few individual samples that do not replicate nor do they reproduce among related tissues. Apart from these individual samples, the pattern is noisy across developmental time. This pattern does not correspond to any known dissection or global QC issue. Also similar to cluster 15, no GO term enrichment was found.
2. The lower clade contains genes that are widely expressed among different tissues that are also systematically depleted in the E11.5 and E14.5 samples. This reflects known batch effects at tissue collection/dissection steps (see also CCA).

**Cluster 18** from hierarchical clustering analysis of bulk RNA samples.

1. Most genes in Cluster 18 are enriched in the craniofacial prominence at early stages but not later.
2. Its top GO terms mainly concern eye development, including “structural constituent of eye lens” (p=1.6e-17) and “eye development” (p=1.1e-15). Genes include crystallins, retinoic acid-metabolizing enzymes (Cyp26a1 and Cyp26c1), lens membrane protein (Lim2), melanin regulators (Tyrp1, Tyr and Pmel) and one developmental regulator (Vax2).
3. The dissection plan for cranioface was to exclude the eyes, but at earlier stages it appears not to have been fully successful. The expression pattern and Gene Ontology, of Cluster 18 genes in the early craniofacial prominence samples (E10.5, E11.5 and E12.5), including sharp transitions between adjacent timepoints, are likely due to imperfect removal of early eyes. Sporadic enrichment of these genes in later stage craniofacial prominence samples (E16.5) is likely due to a few imperfect dissections in a large embryo pool.

**Cluster 19** from hierarchical clustering analysis of bulk RNA samples.

1. Genes in this cluster are mostly enriched in bladder, kidney, limb and neural tube. Within these tissues, expression levels are relatively constant over developmental time.
2. The lower half of this cluster contains 5’ Hox genes (9-13) and lincRNAs localized in the 5’ region of Hox clusters (Hotair and Hottip). Genes with names beginning with “Gm” that are clustered together with Hox genes are also localized in 5’ Hox gene regions, suggesting shared transcriptional regulatory elements or RNA precursors. Among these Hox-cluster genes, there are distinctions, with 5’ Hox expression being more abundant in posterior tissues (e.g. bladder, kidney and intestine), consistent with previous findings.
3. As the time course begins at E10.5, we could not follow the well-known upregulation sequence of Hox genes which displays “temporal co-linearity”, except for a gradual increase in Hoxc12 and Hoxc13 in the limb, which represent the distal ends of limbs and whose upregulation pattern is late enough to be captured in our time window.
4. In E14.5 neural tube samples the 5’ most Hox genes Hox11-13 are missing, because that batch of embryo dissections did not include the posterior tip of the tube.
5. Four major urinary protein (MUP) genes are enriched in limb, similar to the MUP paralogs in Cluster 2. However, unlike those in Cluster 2, they are also enriched in early neural tube samples.

**Cluster 20** from hierarchical clustering analysis of bulk RNA samples.

Overall, genes in Cluster 20 are prominently absent from the liver at all stages and are absent or strongly reduced at P0 in most tissues having P0 data. The top ~1/3 of the cluster contributes little to the two major expression and GO themes. It contains mainly pseudogenes and lncRNAs. In limb, craniofacial prominence and brain, depletion of some of these genes is evident at E11.5 and E14.5 similar to Cluster 17, and possibly related to the batch effect discussed before.

1. In the remaining bottom 2/3, there is considerable substructure among expressing tissues due to the two major biological themes: The first GO enrichment theme is tissue morphogenesis and development, such as “skeletal system morphogenesis” (p=3.3e-13), “branching morphogenesis of an epithelial tube” (p=5.5e-12), “sensory organ development” (p=7.7e-11), “odontogenesis” (p=1.3e-10), “gland development” (4.5e-10), “ossification” (p=9.1e-10), “limb morphogenesis” (p=2.1e-9) and “kidney development” (6.1e-9). The second GO theme is Wnt signaling, such as “regulation of Wnt signaling pathway” (8.2e-12), “Wnt signaling pathway” (2.0e-10) and “Wnt-protein binding” (3.2e-10). Although this cluster called terms covering a variety of different aspects of development demonstrated in the first theme, the driving genes are often shared among multiple terms referring to different tissues. This likely reflects the broad usage of these signaling pathways in patterning and morphogenesis. Moreover, roughly a quarter of the genes contributing to any Theme 1 terms also contribute to the Wnt theme. Other Theme 1 genes that do not currently contribute to the Wnt GO terms, such as Irx3, Runx2, TWIST, Bmp4, Tbx1 and Tbx3 have been independently associated with this signaling system. This is consistent with the current appreciation that Wnt signaling plays an important and widely distributed role in different individual anlage, including stem cell renewal.
2. The clade of genes colored in purple are highly enriched in kidney and moderately enriched in limb and craniofacial prominence. The themes are suggested by Gene Ontology with terms “skeletal system morphogenesis” (p=1.2e-6) and “branching morphogenesis of an epithelial tube” (2.3e-6). These terms of this clade are consistent with the overall theme of this big cluster but the distinct gene expression pattern suggests intensive usage of this subprogram of genes in the kidney.
3. The clade of genes labeled in blue has prominent enrichment in limbs and craniofacial prominence, and is lower but still detectable and decreasing in other tissues. The top GO terms are similar to those called from the whole cluster, but with much enhanced significance for “embryonic skeletal system morphogenesis” (p=1.8e-15) and “cartilage development” (p=1.5e-9).

**Cluster 21** from hierarchical clustering analysis of bulk RNA samples.

1. Genes in Cluster 21 are expressed in all the fetal tissues and decrease over time in most. At P0 the majority are expressed in thymus and spleen but are notably depleted elsewhere.
2. Top GO terms are mainly cell division and nucleus components, such as “chromosomal part” (p=1.2e-93) and “cell cycle process” (p=1.6e-87) consistent with genes involved in executing the cell cycle, especially components of the chromosome and its associated proteins. The global decrease in RNA levels from these genes over time is consistent with shifting from fast growing proliferating cells to more differentiated ones.
3. Fetal hemoglobins are also found in this cluster such as Hbb-bh1, Hbb-y and Hba-x.

**Cluster 22** from hierarchical clustering analysis of bulk RNA samples.

1. Genes in Cluster 22 show distinct enrichment at E11.5 and E14.5 stages in some tissues, and they do so reproducibly among the replicates.
2. Most of these genes are pseudogenes and low-mappability protein-coding genes. They are similar to the batch-effect clades in Cluster 17 and Cluster 20 but display the inverse pattern trend.

**Cluster 23** from hierarchical clustering analysis of bulk RNA samples.

1. This small cluster contains genes most prominently expressed at early times in CNS tissues. They are also depleted preferentially at E16.5 in many other tissues. Unlike the candidate batch effects of clusters like 15, 17, and 22 that are heavily enriched in pseudogenes, this cluster is not explained by annotated pseudogenes. There was no significant GO enrichment.

**Cluster 24** from hierarchical clustering analysis of bulk RNA samples.

1. Genes in this cluster are widely expressed and are preferentially higher in the CNS regions and/or in the developing liver. Most, but not all, increase during development of these tissues.
2. Top Gene Ontology terms are dominated by lipid metabolism, such as “lipid metabolic process” (p=2.7e-13) and “cholesterol biosynthetic process” (p=1.7e-11). Interestingly, all of the nine genes contributing to the term “cholesterol biosynthetic process” are localized in a tiny clade of 23 genes labelled in purple. These 23 genes are all very abundant and highly correlated among themselves.

**Cluster 25** from hierarchical clustering analysis of bulk RNA samples.

1. More than half of the genes in Cluster 25 are consistently and highly enriched in the hindbrain and neural tube plus stomach, intestine and adrenal gland. Kidney and lung also express distinct subsets of these genes.
2. This cluster contains most of the 3’ Hox genes, almost all located in the two clades labeled in purple and blue. The purple clade consists of the 3’ most Hox genes and genes sitting in the 3’ end of Hox gene clusters while the blue clade is made of Hox genes and non-Hox genes in the center (less 3’ but not 5’) of Hox clusters. The purple-clade genes are expressed in lung while the blue ones are mostly not. This is probably because lung is relatively anterior to other endoderm tissues assayed, which corresponds to the 3’ end of the endoderm Hox A/P axis.
3. For the genes outside the Hox gene clades combined, Gene Ontology generated terms related to the neural system, such as “neuron differentiation” (p=1.4e-7); focusing on “enteric nervous system development” (p=2.4e-7).
4. In E14.5 neural tube samples some genes are more depleted compared to E13.5 and E15.5. We think this might result from a dissection protocol detail that produced shorter spinal cord and depleted the 3’-most Hox expressing tissue.

**Cluster 26** from hierarchical clustering analysis of bulk RNA samples.

1. Genes in Cluster 26 are mostly enriched in forebrain at late stages.
2. Avp and Oxt encode neuropeptides synthesized in the hypothalamus that regulate complex maternal and sexual behaviors. They are clustered together within 6kb on chromosome 2.

**Cluster 27** from hierarchical clustering analysis of bulk RNA samples.

1. Cluster 27 contains genes highly enriched in kidney. Most are also expressed in brain and neural tube at later stages but less abundantly than in kidney.
2. Pax2, Pax8 and their target Gata3 are found in this cluster which specify the nephric lineage and regulate branching morphogenesis in the developing kidney. Pax2 and Pax8 are also reported to specify GABAergic and glycinergic neuronal fates, partly explaining expression in the hindbrain and neural tube. It’s possible that this cluster concerns two independent cell fate specification and morphogenesis programs that use overlapping regulatory factor sets, such as Pax2 and 8 and Gata3.

**Cluster 28** from hierarchical clustering analysis of bulk RNA samples.

1. Genes in Cluster 28 are expressed in many tissues, but with lung and craniofacial prominence being highest, followed by CNS regions. Almost all increase over time, but with differing kinetics in different tissues and brain regions.
2. Most of the significant GO terms are about ciliogenesis, such as “cilium movement” (p=1.4e-19), “cilium” (1.2e-17) and “outer dynein arm assembly” (1.5e-14). The contributing genes include components of dynein arms and radial spokes, genes coding for assembly machinery such as dynein docking complex, tubulin modifying enzyme and the nexin-dynein regulatory complex. Two known cilium regulators, Foxj1 and Mcidas, are also in this cluster. The cilium is a fundamental structure, with primary cilia being ubiquitous while secondary and sensory cilia having more specialized distributions that correspond well with the pattern for the majority of genes in cluster 28. The pattern can be explained by the emergence of airway cilia in the lung, the airways of the craniofacial prominence, and the ependymal cilia of the CNS.

**Cluster 29** from hierarchical clustering analysis of bulk RNA samples.

1. Most genes in this relatively small cluster are distinguished by highest expression in the thymus, but more than half are also expressed substantially in brain or in face/limb, or in kidney/lung and gut.
2. Gene Ontology failed to identify a significantly enriched term for this group.

**Cluster 30** from hierarchical clustering analysis of bulk RNA samples.

1. Cluster 30 contains genes expressed most prominently in heart and/or CNS samples, with the admixture among the tissues varying across different clades.
2. Top enriched GO terms mainly identify transport of metal ions, such as “metal ion transport” (p=4.1e-8), metal ion transmembrane transporter activity (p=1.3e-7) and “potassium ion transmembrane transporter activity” (p=2.0e-7).

**Cluster 31** from hierarchical clustering analysis of bulk RNA samples.

1. Genes in Cluster 31 are mainly enriched in brain and neural tube, with different regionalization for subclusters, plus the facial prominence (perhaps partly driven by cross-contamination of face with forebrain dissection at early times).
2. More than a third of the genes in this cluster are transcription factors (“sequence-specific DNA binding”, p=1.0e-26), most of which also contribute to the GO term “neuron differentiation” (p=4.4e-18). It is likely that this group of genes are involved in neuron maturation, such as Dlx1, Dlx2 and Helt which specifies GABAergic neuron differentiation. Genes responsible for cerebral cortex GABAergic interneuron migration (Lhx6, Arx and Fezf2) are also found in this cluster.

**Cluster 32** from hierarchical clustering analysis of bulk RNA samples.

1. Genes in Cluster 32 are expressed in nearly all the tissues with increasing trajectories over time, with the notable exception of liver where they are expressed at very low levels and then decrease. This cluster contributes to the global separation of the CNS (where expression is strongest) from the developing liver.
2. Gene Ontology offered little specific annotation, except “positive regulation of adenylate cyclase activity” (p=1.7e-5).

**Cluster 33** from hierarchical clustering analysis of bulk RNA samples.

1. Genes in the large cluster 33 are expressed in most tissues prior to P0, except liver. CNS and face/limb are by far the most prominent. Most of these genes are time-course variant. Timecourses in different tissues display distinctive trajectories, with decreasing courses being more common, unlike most other major clusters. Thus genes in this cluster vanish very early in liver; decrease monotonically in kidney, lung, stomach and intestine; remain constant early and slightly decrease at later stages in heart, craniofacial prominence, and limb.
2. Gene Ontology enrichment produced three major themes. First, 159 genes (16%) encode DNA binding proteins - especially transcription factors - contributing to “DNA binding” (p=1.7e-17) and “RNA biosynthetic process” (p=7.5e-14). As discussed in the Results section, zinc finger presumptive repressors are especially prominent (Ext. Data Fig. 7e). Second, this cluster contains genes regulating different aspects of morphogenetic processes, with enrichment in the term “embryonic morphogenesis” (p=1.9e-12). This is similar to Cluster 20, which also has a broadly decreasing pattern, though it features an emphasis on the Wnt pathway that does not apply to Cluster 33. Finally, significant overlaps of Cluster 33 with cell projection-related genes are called by terms “cell projection organization” (p=1.3e-15), “cilium assembly” (p=1.9e-14), “neuron projection guidance” (p=4.4e-11) and “regulation of nervous system development” (p=4.2e-12). This cluster of genes is different from the cilium-related Cluster 28 in expression dynamics, showing opposite temporal trajectories that argue strongly for distinct regulation.

**Cluster 34** from hierarchical clustering analysis of bulk RNA samples.

1. The theme of this large cluster is expression in all four CNS tissues, with a dominant upward temporal pattern. While most increase over time, they do so with varying kinetics among subclades and between brain regions. Gene Ontology revealed enrichment for a large number of neuron-identity and structure terms associated with neuronal differentiation and maturation, with the most dominant ones being “synapse” (p=1.0e-93), “neuron projection” (p=6.9e-55), “behavior” (p=2.3e-42) and “regulation of nervous system development” (p=2.9e-34).
2. Apart from the central neuronal theme, subclades (colored purple, blue and pink) differ from each other and from the major neural cluster. All three are significantly enriched with transcription factors and neural development regulators, and they display diverse tissue patterns relative to each other. The small purple clade at the top features genes enriched caudally in neural tube and hindbrain. The blue clade below it is enriched in midbrain and significantly but less so in hindbrain and neural tube, with overall downward trajectories. The pink clade near the bottom features genes expressed earliest in all four CNS regions, diminishing in later stages.

**Supplementary Note 2: Inferring cell types, states, and stages**

Differentially expressed genes for each cell cluster were calculated separately for C1 and 10X datasets using Seurat’s FindMarkers (https://satijalab.org/seurat/seurat_clustering_tutorial_part2.html) with min.pct = 0.25, applying its Wilcoxon rank sum test, and setting min.diff.pct to be 0.2 or 0.4. The differential gene lists are provided in full in Supplementary Data 4, and representative top markers for each cluster are given in Supplementary Tables 1 and 2. The top marker genes for each cell cluster are shown in Fig. 3b where each cell cluster was down-sampled to at most 100 cells for 10x data and at most 50 cells for C1 data. To associate cell clusters with likely cell types/stages, the differential marker expression lists with min.diff.pct = 0.2 for each cluster were entered into FuncAssociate 3.0 (http://llama.mshri.on.ca/funcassociate/)^1^, which returned GO attributes that were evaluated by LOD score and P values, requiring that all terms used be Padj <.05, corrected for multiple testing by empirical resampling. Attributes emphasizing terms that are explicitly or implicitly pertinent to developmental processes and cell types were selected by inspection and the appropriate attribute/entity tables were downloaded. The gene symbols from the attribute/entity tables were then used to search the literature to assemble curated marker genes and references (Supplementary Tables 1 and 2). Key confirmatory literature markers for a candidate cell type were evaluated for significantly differential marker status (Supplementary Data 4) and included as literature markers. In cases where the literature provided a clear naming convention for a cell type association, we adopted that name in the tables. In other cases, the name is assigned from this work (e.g. Ihh+ chondrocyte). When marker ambiguity prevented an unequivocal cell type assignment, we chose a designation based on the weight of the available evidence that is consistent with known fate mapping, tracing and cell sorting studies.

Our cell type identification was performed independently for data from the two platforms. We then evaluated correlations between the clusters, labeled by their cell type assignments, by logistic regression using all the detected genes (Supplementary Fig. 2). The core set of 11 major cell types and stages, displayed identity broadly across their entire transcriptomes (Fig 3). Cell types with multiple developmental stages or subdivisions show splitting of C1 state-specific genes across 2 or more 10x clusters (e.g. mus1, mus2, mus3 and mus4), as would be expected from differences in cell sampling depth and transcriptome-depth.


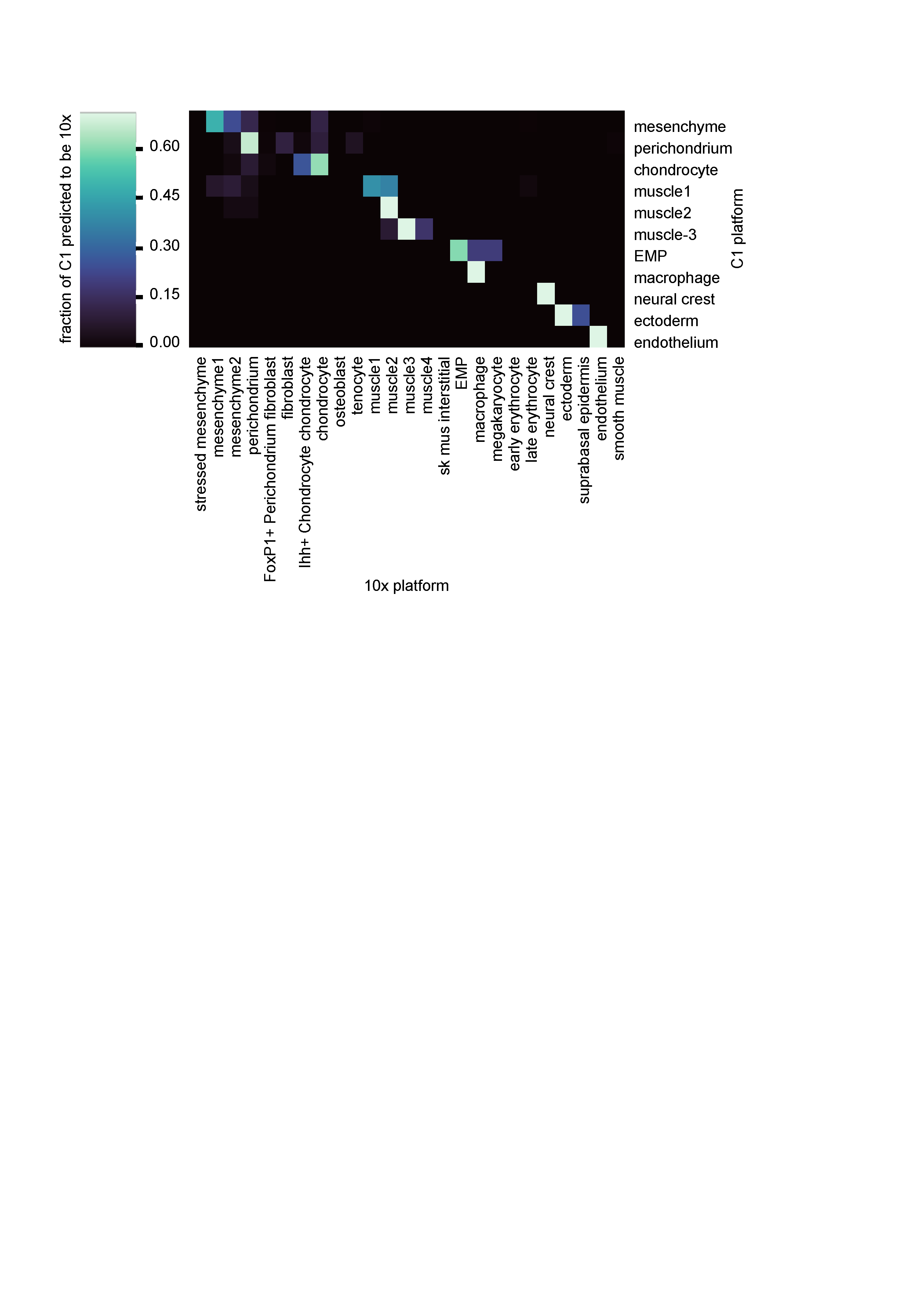


**Supplementary Figure 2.** **Cross-comparison of cell type assignments across the two scRNA-Seq platforms**. Logistic regression results are plotted for all pairwise comparisons of cell type assignments across both scRNA-Seq platforms. Scale bar represents the fractional proportion of a C1 cell type predicted to be each 10x cell type. For cell types found in the 10x Genomics data that were not found in the C1 Fluidigm data, the scores are set at 0. Both 10x and C1 data were normalized, log-transformed and scaled before regression. To train a multinomial logistic regression model using the scikit-learn python package, the categorical information of the 25 cell types from 10x were used as the dependent variable. The trained model was then used to predict the C1 data. This model assigned each C1 single-cell into one of the 25 cell type categories defined by 10x data. The distribution (fraction) of the assignment per each C1 cell type into 10x categories was visualized as a row in the heatmap.

Fidelity of cell stage identification was preserved at the level of TF expression. In the compartments of the skeletal muscle lineage (Supplementary Figure 3 and Extended Data Fig. 12f) the sensitivity of transcript detection and cell type identification between the two methods can be compared at a glance. The 10x Genomics method (left panel) identifies an additional late stage myocyte compartment (dark red block) that is absent in the C1 data, due to cell population undersampling and possible mechanical loss of large multinucleate nuclei in the fixed channel size Fluidigm apparatus. The higher sensitivity C1 method (right panel) identifies precocious lineage-specific expression of MyoD1, Hes6 and Zbtb18 in muscle precursor cells (green block) that is missed in the 10x method which has a lower Psmc.


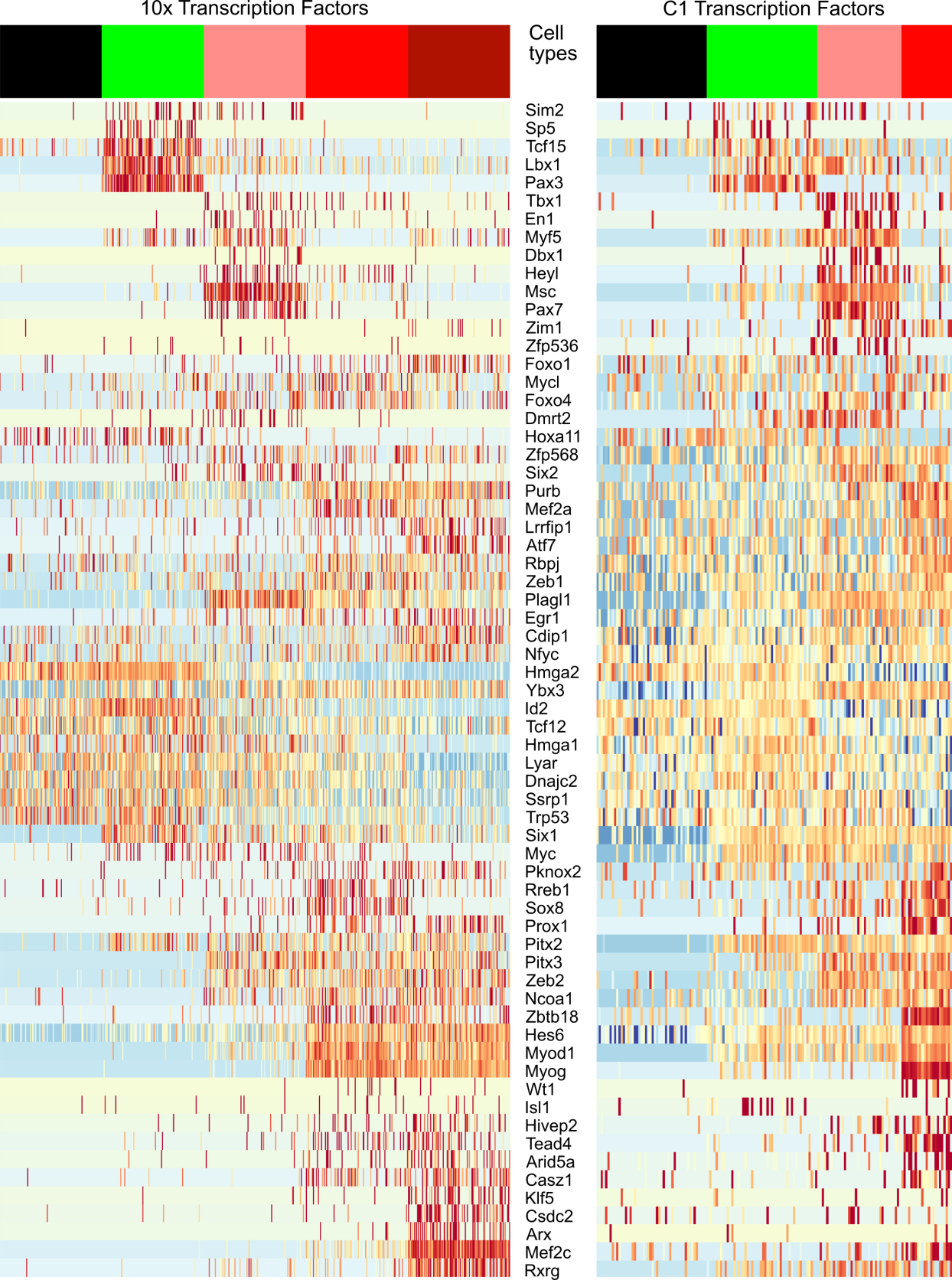


**Supplementary Figure 3:** Heatmaps for all TFs that scored as differential genes for cell types in the myogenic lineage plus limb resident mesenchyme, at 0.2 ∂pct cutoff, downsampled to 100 (10x) or 30 (C1). Heat map code per Extended Data Fig. 12f. Cell stages across the top: black-Mesenchyme1; green-Muscle1, pink-Muscle2; red-Muscle3; dark red-Muscle4.

**Cell type sensitivity.** The greater cell sampling depth of the 10x Genomics data (~90,000 cells) detected 14 additional clusters with their own provisional cell-type assignments. Three of the 10x-only cell types were expected to be absent from C1 data due to platform-specific details of cell-size filtration (early and late erythrocytes) and sample preparation (prior removal of sticky epidermal cells). The remaining 10x only clusters include rarer cell types/states with no C1 equivalent, presumably due mainly to sparse cell sampling with C1 plus some additional subdivision of C1 types into multiple 10x types.

**Marker gene sensitivity.** For several of the best-studied cell types we found that the C1 data identified key known markers from the literature that were not in the corresponding 10x differential lists. Examples that are functionally important include Pthlh, a regulator of the proliferation/differentiation choice in early chondrocytes^2,3^; Spry1, which regulates quiescence in Pax7 muscle precursor cells^4^ that comprise the Mus2 cluster; and Dmrt2, which is directly regulated by Pax3 in muscle precursors (Mus1), and in turn directly regulates myf5^5^, which marks mus1 and mus2 in C1 data. None of these were significant markers in the corresponding 10x cell types, and all three were expressed at low levels in the corresponding C1 clusters (Pthlh: 6 copies per cell (cpc)); Spry1, 1 cpc; and Dmrt2, 3 cpc).

**Marker gene context.** Defining a marker gene or a multigene cell-type signature also depends heavily on the tissue sample context. This is a pertinent caution for future uses of the marker gene sets derived here, if they are applied to other studies in different biological settings. Conversely, this issue also informed our use of markers from the literature, where we favored the closest context available, although a strong match was often unavailable. Overall, our cell type identity assignments did prove consistent with classical and modern tracing studies and genetic knockouts for the better-studied cell types. We further observed that cell-type marker signatures increased in complexity as several lineages progressed (Fig. 3b and Extended Data Fig. 12a), making the more mature types (e.g. Muscle 3 or Macrophage) easier to define with high confidence. By contrast, their progenitors (Muscle1, EMP and Mesenchyme1) displayed lower salience signatures, with few progenitor-unique genes compared with their more differentiated counterparts.

**Cluster structure sensitivity**. It is important to recognize that the relationship of a cell cluster with a dominant cell identity does not (and is not expected to) preclude the presence of additional cell types within the cluster. At this early level of single cell resolution, it is expected that much additional cell type structure is unresolved. For example, in the cell clusters identified here as predominantly EMP, close examination of candidate marker gene sets from the literature found some evidence for the presence of related cells (mast cells), as noted in Supplementary Tables 1 and 2.

**Supplementary Table 1**

**Fluidigm C1 marker genes and inferred cell identities, states and stages**

| **cell Cluster #** | **cell cluster name** | **provisional cell type/state** | **GO ID** | **GO terms from Fluidigm data** | **LOD score** | **P unadj.** | **P adj.** | **top5 genes from C1 clusters** | **selected literature marker genes** |
| --- | --- | --- | --- | --- | --- | --- | --- | --- | --- |
| 0 | mesenchyme (mes) | mesenchyme | GO:0035115 | embryonic forelimb morphogenesis | 1.67 | 2.71E-07 | <0.001 | [Mecom, Hsd11b2, Lix1, Car14, Hoxd10] | [Hoxd10^6^, Hoxd11^6^, Hoxd12^6^, Msx1^7^, Twi1^8,9^] |
|  |  |  | GO:0009954 | proximal/distal pattern formation | 1.67 | 4.69E-06 | 0.006 |  |  |
|  |  |  | GO:0042733 | embryonic digit morphogenesis | 1.66 | 4.75E-12 | <0.001 |  |  |
|  |  |  | GO:0009952 | anterior/posterior pattern specification | 1.15 | 3.95E-07 | <0.001 |  |  |
|  |  |  |  |  |  |  |  |  |  |
| 1 | perichondrium (pchon) | perichondrium | GO:0060351 | cartilage development involved in endochondral bone morphogenesis | 1.41 | 7.20E-06 | 0.032 | [Col6a1, Egfl6, Creb3l1, Ogn, Col1a1] | [Col1a1^10^, Dcn^11^, Dkk3^12^, Ogn^11^, Thbs2^12^] |
|  |  |  | GO:0030199 | collagen fibril organization | 1.27 | 1.33E-08 | <0.001 |  |  |
|  |  |  | GO:0001649 | osteoblast differentiation | 0.98 | 7.79E-06 | 0.039 |  |  |
|  |  |  | GO:0001503 | ossification | 0.94 | 7.67E-09 | <0.001 |  |  |
|  |  |  |  |  |  |  |  |  |  |
| 2 | chondrocytes (chon) | immature chondrocytes | GO:0060351 | cartilage development involved in endochondral bone morphogenesis | 1.64 | 4.74E-08 | <0.001 | [Susd5, Matn1, Foxa3, Ncmap, Acan] | [Acan^3^, Col2a1^3^, Dlx5^13^, Ihh^3^, Pthlh^3^, Runx2^13^, Runx3^14^, Sox5^3^, Sox6^3^, Sox9^3^, Sp7^13^] |
|  |  |  | GO:0032331 | negative regulation of chondrocyte differentiation | 1.55 | 1.35E-07 | <0.001 |  |  |
|  |  |  | GO:0001958 | endochondral ossification | 1.54 | 1.01E-09 | <0.001 |  |  |
|  |  |  | GO:0001502 | cartilage condensation | 1.50 | 2.47E-06 | 0.006 |  |  |
|  |  |  | GO:0030279 | negative regulation of ossification | 1.16 | 8.08E-11 | <0.001 |  |  |
|  |  |  | GO:0001649 | osteoblast differentiation | 1.16 | 7.21E-08 | <0.001 |  |  |
|  |  |  |  |  |  |  |  |  |  |
| 3 | muscle3 (mus3) | myocyte | GO:0003009 | skeletal muscle contraction | 1.96 | 1.29E-18 | <0.001 | [Gm7325, Ablim3, Kcnk13, Smyd1, Klhl41] | [Actc1^15^, Act3^15^, Fgfr4^16^, Myod1^16^, Myog^16^, Myot^15^, Ryr1^15^, Tead4^15^] |
|  |  |  | GO:0048741 | skeletal muscle fiber development | 1.54 | 1.33E-10 | <0.001 |  |  |
|  |  |  | GO:0007520 | myoblast fusion | 1.42 | 6.50E-07 | <0.001 |  |  |
|  |  |  |  |  |  |  |  |  |  |
| 4 | muscle1 (mus1) | migratory limb muscle precursor cell | GO:0007517 | muscle organ development | 1.45 | 2.67E-10 | <0.001 | [Pax3, Lbx1, Pitx2, Myod1, Eya1] | [Dmrt2^5^, Eya1^16^, Eya2^16^, Lbx1^16^, Met^17^, Msc^16^, Myf5^16^, Myod1^16^, Pax3^18–20^, Pitx2^16^, Pitx3^16^] |
|  |  |  | GO:0007519 | skeletal muscle tissue development | 1.50 | 1.32E-08 | <0.001 |  |  |
|  |  |  | GO:0001756 | somitogenesis | 1.35 | 8.55E-06 | 0.018 |  |  |
|  |  |  |  |  |  |  |  |  |  |
| 5 | neural crest (neur) | neural crest | GO:0007422 | peripheral nervous system development | 1.62 | 8.84E-06 | 0.015 | [Gpr17, Foxd3, St8sia5, Lgi4, Insc] | [Dlx1^21^, Dlx2^21^, Foxd3^22^, Sox2^22^, Sox10^22^, Zeb2^22^] |
|  |  |  | GO:0042552 | myelination | 1.17 | 1.68E-06 | 0.003 |  |  |
|  |  |  | GO:0045666 | positive regulation of neuron differentiation | 0.76 | 3.46E-07 | <0.001 |  |  |
|  |  |  |  |  |  |  |  |  |  |
| 6 | erythro-myeloid precursor (EMP)* | erythro-myeloid precursor | GO:0006909 | phagocytosis | 1.05 | 1.36E-07 | 0.001 | [Slc22a3, 1110028F11Rik, Ubash3a, Rab44, Gata1] | [Fcgr3^23^, Gata1^23^, Gata2^23^, Gfi1b^24^, Spi1^23^] |
|  |  |  | GO:0051707 | response to other organism | 0.53 | 3.08E-07 | 0.001 |  |  |
|  |  |  | GO:0002478 | antigen processing and presentation of exogenous peptide antigen | 1.39 | 9.96E-07 | 0.003 |  |  |
|  |  |  |  |  |  |  |  |  |  |
| 7 | endothelium (endo) | endothelium | GO:0001945 | lymph vessel development | 1.81 | 9.26E-09 | <0.001 | [Ccm2l, Myct1, Pcdh12, Sox17, Robo4] | [Aplnr^25^, Flt4^26^, Kdr^26^, Lmo2^27^, Sox17^27^] |
|  |  |  | GO:0002040 | sprouting angiogenesis | 1.43 | 5.46E-08 | <0.001 |  |  |
|  |  |  | GO:0061028 | establishment of endothelial barrier | 1.39 | 8.34E-07 | <0.001 |  |  |
|  |  |  | GO:0001525 | angiogenesis | 1.17 | 1.67E-28 | <0.001 |  |  |
|  |  |  |  |  |  |  |  |  |  |
| 8 | muscle2 (mus2) | myoblast | GO:0007517 | muscle organ development | 1.31 | 1.57E-12 | <0.001 | [Pax7, Ntrk1, Scn3b, Gm9947, Myf5] | [Dmrt2^5^, En1^28^, Eya1^16^, Eya2^16^, Hes6^29^, Lbx1^16^, Met^17^, Msc^16^, Myf5^16^, Myod1^16^, Notch3^30^, Pax7^31^, Pitx2^16^, Pitx3^16^, Six1^16^, Six2^16^, Sox8^32^, Spry1^4^, Vgll2^33^, Vgll3^34,35^] |
|  |  |  | GO:0061061 | muscle structure development | 1.27 | 4.10E-12 | <0.001 |  |  |
|  |  |  | GO:0051147 | regulation of muscle cell differentiation | 1.05 | 4.95E-12 | <0.001 |  |  |
|  |  |  |  |  |  |  |  |  |  |
|  |  |  |  |  |  |  |  |  |  |
| 9 | macrophage (mac) | tissue resident macrophage | GO:0098542 | defense response to other organism | 1.01 | 5.56E-44 | <0.001 | [Lrrc25, Clec4a3, Cybb, Fcgr1, AI607873] | [Csf1r^23^, Cx3cr1^23^, Emr1^23^, Fcer1g^23^, Irf8^23^, Runx1^36^, Spi1^23^] |
|  |  |  | GO:0045087 | innate immune response | 1.03 | 1.00E-41 | <0.001 |  |  |
|  |  |  | GO:0001819 | positive regulation of cytokine production | 0.95 | 2.31E-37 | <0.001 |  |  |
|  |  |  | GO:0006935 | chemotaxis | 0.98 | 9.62E-28 | <0.001 |  |  |
|  |  |  |  |  |  |  |  |  |  |
| 10 | ectoderm (ecto) | ectoderm | GO:0005109 | frizzled binding | 1.55 | 1.13E-06 | 0.001 | [4631405K08Rik, Ap1m2, Fermt1, Krt5, Krt14] | [Fzd6^37^, Grhl2^38^, Grhl3^38^, Klf5^39^, Krt1^39^, Krt17^39^, Wnt3^37^, Wnt4^37^, Wnt6^37^, Wnt7a^37^, Wnt7b^37^, Wnt10a^37^] |
|  |  |  | GO:0035567 | non-canonical Wnt signaling pathway | 1.53 | 1.38E-06 | 0.002 |  |  |
|  |  |  | GO:0030216 | keratinocyte differentiation | 1.41 | 5.53E-08 | <0.001 |  |  |
|  |  |  | GO:0035136 | forelimb morphogenesis | 1.28 | 1.87E-05 | 0.038 |  |  |
|  |  |  | GO:0016055 | Wnt signaling pathway | 0.89 | 2.29E-07 | <0.001 |  |  |
|  |  |  | GO:0001228 | transcriptional activator activity, RNA polymerase II transcription regulatory region sequence-specific binding | 0.80 | 4.68E-08 | <0.001 |  |  |
|  |  |  | GO:0007389 | pattern specification process | 0.68 | 1.45E-05 | 0.031 |  |  |
|  |  |  | GO:0048646 | anatomical structure formation involved in morphogenesis | 0.61 | 9.81E-07 | 0.001 |  |  |

* 2 cells in this cluster expressed mast cell genes.

**Supplementary Table 2**

**10x Genomics marker genes and inferred cell identities, states and stages**

| **Cell Cluster #** | **cell cluster name** | **provisional cell type/state** | **GO ID** | **GO terms from 10x Genomics data** | **LOD score** | **P unadj.** | **P adj.** | **top5 genes from 10x clusters** | **selected literature marker genes** |
| --- | --- | --- | --- | --- | --- | --- | --- | --- | --- |
| 0 | proximal mesenchyme (mesprox) | proximal mesenchyme | GO:0030326 | embryonic limb morphogenesis | 1.22 | 3.72E-09 | <0.001 | [Lix1, Asb4, Igdcc3, Hmga2, Rrm2] | [Hoxa9^6^, Hoxd9^6^, Hoxd10^6^, Hoxd11^6^, Shox2^40^] |
|  |  |  | GO:0048704 | embryonic skeletal system morphogenesis | 1.06 | 7.90E-06 | 0.018 |  |  |
|  |  |  | GO:0022402 | cell cycle process | 0.70 | 1.06E-08 | <0.001 |  |  |
|  |  |  |  |  |  |  |  |  |  |
| 1 | perichondrium (pchon) | perichondrium | GO:0005583 | fibrillar collagen trimer | 2.48 | 2.86E-12 | <0.001 | [Dlk1, Meg3, Col3a1, Igf1, Col1a1] | [Col1a1^10^, Col5a1^41^, Igfbp3^42^, Shox2^40^] |
|  |  |  | GO:0001649 | osteoblast differentiation | 1.44 | 3.22E-08 | 0.001 |  |  |
|  |  |  | GO:0009653 | anatomical structure morphogenesis | 0.59 | 3.33E-07 | 0.002 |  |  |
|  |  |  |  |  |  |  |  |  |  |
| 2 | distal mesenchyme (mesdist) | distal mesenchyme | GO:0030326 | embryonic limb morphogenesis | 2.63 | 8.06E-21 | <0.001 | [Msx1, Hoxd13, Prrx2, Hoxa11os, Hoxd12 ] | [Hoxa10^6^, Hoxd12^6^, Hoxd13^6^, Msx1^7^, Msx2^7^] |
|  |  |  | GO:0042733 | embryonic digit morphogenesis | 2.48 | 1.37E-14 | <0.001 |  |  |
|  |  |  | GO:0007389 | pattern specification process | 1.74 | 1.31E-10 | <0.001 |  |  |
|  |  |  | GO:0009952 | anterior/posterior pattern specification | 1.93 | 1.24E-09 | <0.001 |  |  |
|  |  |  | GO:0051216 | cartilage development | 2.12 | 2.98E-09 | <0.001 |  |  |
|  |  |  |  |  |  |  |  |  |  |
| 3 | chondrocytes (chon) | immature chondrocytes | GO:0060174 | limb bud formation | 2.54 | 3.21E-07 | <0.001 | [Sox9, Col2a1, Wwp2, Col9a1, Gdf5] | [Acan^3,14^, Col2a1^3,14^, Sox5^3,14^, Sox6^3,14^, Sox9^3,14^] |
|  |  |  | GO:0002063 | chondrocyte development | 2.38 | 1.09E-08 | <0.001 |  |  |
|  |  |  | GO:0001503 | ossification | 1.63 | 4.41E-07 | 0.001 |  |  |
|  |  |  |  |  |  |  |  |  |  |
| 4 | muscle2 (mus2) | early myoblast | GO:0007519 | skeletal muscle tissue development | 1.57 | 4.40E-09 | <0.001 | [Msc, Kcne1l, Itm2a, Pdgfa, Vgll2] | [Msc^16^, Myf5^16^, Myod1^16^, Pax7^31^, Pitx2^16^, Six1^16^, Vgll2^33^] |
|  |  |  | GO:0014706 | striated muscle tissue development | 1.39 | 7.43E-09 | <0.001 |  |  |
|  |  |  | GO:0007389 | pattern specification process | 0.93 | 3.52E-08 | <0.001 |  |  |
|  |  |  |  |  |  |  |  |  |  |
| 5 | ectoderm (ecto) | ectoderm | GO:0010482 | regulation of epidermal cell division | 3.02 | 3.11E-07 | <0.001 | [Cxcl14, Pdgfa, Wnt6, Krt14, Gjb2] | [Cxcl14^43^, En1^44^, Fzd6^37^, Fzd10^37^, Wnt4^37^, Wnt6^37^, Wnt7b^37^, Wnt10a^37^] |
|  |  |  | GO:0003334 | keratinocyte development | 1.85 | 1.42E-06 | 0.001 |  |  |
|  |  |  | GO:0043588 | skin development | 1.67 | 1.39E-15 | <0.001 |  |  |
|  |  |  | GO:0009954 | proximal/distal pattern formation | 1.53 | 1.44E-06 | 0.001 |  |  |
|  |  |  |  |  |  |  |  |  |  |
| 6 | fibroblast (fibro) | fibroblast | GO:0048407 | platelet-derived growth factor binding | 2.14 | 1.41E-10 | <0.001 | [Crabp1, Crip1, Twist2, Lum, Rspo1] | [Irx1^45^, Irx2^45^, Irx3^45^, Irx5^45^, Tbx15^46^, Twist2^47^] |
|  |  |  | GO:0045667 | regulation of osteoblast differentiation | 1.11 | 5.88E-09 | <0.001 |  |  |
|  |  |  | GO:0030278 | regulation of ossification | 0.97 | 1.06E-08 | <0.001 |  |  |
|  |  |  | GO:0048705 | skeletal system morphogenesis | 0.97 | 7.70E-06 | 0.011 |  |  |
|  |  |  |  |  |  |  |  |  |  |
| 7 | muscle1 (mus1) | migratory limb muscle precursor cells | GO:0014706 | striated muscle tissue development | 1.13 | 3.02E-06 | 0.007 | [Pax3, Lbx1, Tcf15, Ppp1r14b, Slc25a5] | [Lbx1^16^, Met^17^, Pax3^18–20^] |
|  |  |  | GO:0048562 | embryonic organ morphogenesis | 1.06 | 3.72E-07 | <0.001 |  |  |
|  |  |  | GO:0060537 | muscle tissue development | 1.05 | 9.13E-06 | 0.021 |  |  |
|  |  |  |  |  |  |  |  |  |  |
| 8 | macrophage (mac) | macrophage | GO:0048246 | macrophage chemotaxis | 1.41 | 1.52E-06 | 0.002 | [Apoe, Fcer1g, Tyrobp, C1qb, C1qc] | [Aif1^23^, Cx3cr1^23^, Emr1^23^, Csf1r^23^, Fcgr1^23^, Fcgr3^23^, Grn^23^, Irf8^23^, Maf^23^, Spi1^23^, Zeb2^23^] |
|  |  |  | GO:0019884 | antigen processing and presentation of exogenous antigen | 1.40 | 6.53E-11 | <0.001 |  |  |
|  |  |  | GO:0006911 | phagocytosis, engulfment | 1.19 | 1.57E-08 | <0.001 |  |  |
|  |  |  |  |  |  |  |  |  |  |
| 9 | endothelium (endo) | endothelium | GO:0001945 | lymph vessel development | 1.55 | 2.91E-07 | <0.001 | [Gng11, S100a16, Egfl7, Cdh5, Crip2] | [Aplnr^25^, Efnb2^48^, Ets1^49^, Ets2^49^, Flt4^26^, Kdr^26^, Sox17^27^] |
|  |  |  | GO:0045446 | endothelial cell differentiation | 1.35 | 2.77E-07 | <0.001 |  |  |
|  |  |  | GO:1903672 | positive regulation of sprouting angiogenesis | 1.24 | 1.14E-06 | <0.001 |  |  |
|  |  |  |  |  |  |  |  |  |  |
| 10 | FoxP + perichondrium (pchon^FoxP+)^ | perichondrial cells expressing FoxP1 | GO:0030199 | collagen fibril organization | 1.98 | 3.15E-10 | <0.001 | [Foxp1, Foxf2, Foxq1, Rprm, Pid1] | [Col1a1^10^, Emx2^50^, FoxP1^51^, Lmx1b^50,52^] |
|  |  |  | GO:0001501 | skeletal system development | 1.32 | 1.15E-05 | 0.021 |  |  |
|  |  |  | GO:0009653 | anatomical structure morphogenesis | 0.84 | 5.30E-08 | <0.001 |  |  |
|  |  |  |  |  |  |  |  |  |  |
| 11 | tenocytes (teno) | tenocytes | GO:0035989 | tendon development | 2.98 | 3.88E-07 | 0.002 | [Col1a1, Tnmd, Col1a2, Scx, Ogn] | [Col5a1^53^, Col5a2^53^, Col11a1^53^, Mkx^54^, Scx^55^, Selm^56^, Thbs4^55^, Tnmd^55^] |
|  |  |  | GO:0005583 | fibrillar collagen trimer | 2.31 | 2.93E-11 | <0.001 |  |  |
|  |  |  | GO:0061448 | connective tissue development | 1.24 | 4.21E-06 | 0.007 |  |  |
|  |  |  |  |  |  |  |  |  |  |
| 12 | muscle4 (mus4) | myocyte | GO:0035995 | detection of muscle stretch | 2.60 | 2.43E-07 | 0.001 | [Mylpf, Myl1, Actc1, Tnnc1, Myl4] | [Actc1^15^, Chrng^15^, Jsrp1^15^, Myl1^15^, Mylpf^15^, Ryr1^15^, Sln^15^, Tnnc1^15^, Tnnt1^15^] |
|  |  |  | GO:0070296 | sarcoplasmic reticulum calcium ion transport | 1.98 | 2.95E-10 | <0.001 |  |  |
|  |  |  | GO:0003009 | skeletal muscle contraction | 1.81 | 1.18E-16 | <0.001 |  |  |
|  |  |  |  |  |  |  |  |  |  |
| 13 | early erythrocyte (eryth1) | early erythrocyte | GO:0005833 | hemoglobin complex | 3.38 | 3.95E-16 | <0.001 | [Hba-x, Hbb-bh1, Hba-a1, Hba-a2, Hbb-bt] | [Gata1^57^, Hbb-y^57^, Klf1^57^, Lyl1^57^, Spta1^57^, Sptb^57^, Tal1^57^, Zfpm1^57^] |
|  |  |  | GO:0048821 | erythrocyte development | 1.98 | 7.18E-13 | <0.001 |  |  |
|  |  |  | GO:0030097 | hemopoiesis | 1.13 | 5.32E-07 | 0.002 |  |  |
|  |  |  |  |  |  |  |  |  |  |
| 14 | neural crest (neur) | neural crest | GO:0014044 | Schwann cell development | 1.72 | 5.81E-06 | 0.017 | [Ednrb, Arpc1b, Plp1, Fabp7, Phactr1] | [Ets1^22^, Foxd3^22^, Lmo4^58^, Metrn^59^, Pax3^22^, Sox5^22^, Sox10^22^, Zeb2^22^] |
|  |  |  | GO:0001755 | neural crest cell migration | 1.25 | 6.86E-07 | 0.002 |  |  |
|  |  |  | GO:0010001 | glial cell differentiation | 1.19 | 4.66E-11 | <0.001 |  |  |
|  |  |  |  |  |  |  |  |  |  |
| 15 | (mesX) | mesenchymal cells expressing cell stress genes | GO:0034663 | endoplasmic reticulum chaperone complex | 1.84 | 1.17E-10 | <0.001 |  | [Dnajc10^60^, Hspa5^61^, Hsp90^62^] |
|  | stressed mesenchyme | | GO:0098803 | respiratory chain complex | 1.19 | 6.90E-13 | <0.001 |  |  |
|  |  |  | GO:0045454 | cell redox homeostasis | 1.10 | 1.82E-10 | <0.001 |  |  |
|  |  |  |  |  |  |  |  |  |  |
| 16 | osteoblast (ost) | osteoblast | GO:0001958 | endochondral ossification | 1.40 | 1.80E-09 | <0.001 | [Ibsp, Ifitm5, Smpd3, Sgms2, Sp7] | [Col1a1^10^, Dlx5^13^, Mef2c^63^, Pth1r^64^, Runx2^13^, Sp7^13^] |
|  |  |  | GO:0002062 | chondrocyte differentiation | 1.21 | 7.45E-09 | <0.001 |  |  |
|  |  |  | GO:0001649 | osteoblast differentiation | 1.04 | 3.86E-08 | 0.001 |  |  |
|  |  |  |  |  |  |  |  |  |  |
| 17 | muscle3 (mus3) | late myoblast | GO:0003009 | skeletal muscle contraction | 1.88 | 1.71E-14 | <0.001 | [Myog, Actc1, Tnnt1, Acta2, Vgll2] | [Dll1^65^, Mef2c^16^, Myod1^16^, Myog^16^, Pitx2^16^, Six1^16^, Sox8^32^, Tnnc1^15^, Tnnt1^15^, Vgll2^33^, Zbtb18^66^] |
|  |  |  | GO:0007519 | skeletal muscle tissue development | 1.36 | 3.09E-11 | <0.001 |  |  |
|  |  |  | GO:0045661 | regulation of myoblast differentiation | 1.24 | 1.78E-08 | <0.001 |  |  |
|  |  |  |  |  |  |  |  |  |  |
| 18 | suprabasal epithelium (sup epi) | suprabasal epithelium | GO:0061436 | establishment of skin barrier | 1.75 | 3.75E-13 | <0.001 | [Krtdap, Perp, Hspb1, Sfn, Krt5] | [Krt1^39^, Krt10^39^, Krt14^39^, Krtdap^39^, Notch1^39^, Sbsn^39^, Trp63^39^] |
|  |  |  | GO:0003334 | keratinocyte development | 1.72 | 2.91E-08 | <0.001 |  |  |
|  |  |  | GO:0008544 | epidermis development | 1.31 | 2.35E-12 | <0.001 |  |  |
|  |  |  |  |  |  |  |  |  |  |
| 19 | smooth muscle (smm) | smooth muscle | GO:0014910 | regulation of smooth muscle cell migration | 1.04 | 1.49E-07 | <0.001 | [Acta2, Rgs5, Tagln, Ndufa4l2, Rasgrp2] | [Acta2^67^, Cav1^68^,Coro1b^69^, Cspg4^68^, Cyr61^68^, Egr1^68^, Jag1^68^, Pten^67^, Tagln^67^, Tagln2^67^] |
|  |  |  | GO:0001525 | angiogenesis | 0.93 | 2.08E-14 | <0.001 |  |  |
|  |  |  | GO:0048514 | blood vessel morphogenesis | 0.91 | 1.98E-06 | 0.006 |  |  |
|  |  |  | GO:0060537 | muscle tissue development | 0.88 | 3.59E-06 | 0.012 |  |  |
|  |  |  |  |  |  |  |  |  |  |
| 20 | erythro-myeloid precursor (EMP) * | erythro-myeloid precursor | GO:0030099 | myeloid cell differentiation | 0.90 | 9.59E-14 | <0.001 | [Cma1, Srgn, Cpa3, Rac2, Tyrobp] | [Cd34^70^, Fcgr3^23^, Gata1^23^, Gata2^23^, Kit^23^, Spi1^23^] |
|  |  |  | GO:0050764 | regulation of phagocytosis | 0.85 | 2.17E-06 | 0.009 |  |  |
|  |  |  | GO:0002821 | positive regulation of adaptive immune response | 0.79 | 2.80E-06 | 0.011 |  |  |
|  |  |  | GO:0002819 | regulation of adaptive immune response | 0.79 | 9.95E-09 | <0.001 |  |  |
|  |  |  |  |  |  |  |  |  |  |
| 21 | megakaryocyte (meg) | megakaryocyte | GO:0030220 | platelet formation | 1.57 | 7.03E-12 | <0.001 | [Pf4, Ppbp, Ctla2a, Gp1bb, Gp9] | [Fli1^71^, Gata1^71^, Nfe2^71^, Mef2c^71^, Meis1^72^, Runx1^71^] |
|  |  |  | GO:0070527 | platelet aggregation | 1.29 | 4.23E-09 | <0.001 |  |  |
|  |  |  | GO:0035855 | megakaryocyte development | 1.25 | 1.70E-06 | 0.006 |  |  |
|  |  |  |  |  |  |  |  |  |  |
| 22 | interstitial fibroblast (int/mus) | interstitial fibroblast | GO:0005861 | troponin complex | 3.33 | 4.35E-18 | <0.001 | [Mylpf, Acta1, Myl1, Myl4, Sln] | [Col3a1^73^, Col6a1^73^, Col6a3^73^, Cxcl12^73^, Lum^73^, Osr1^73^, Vim^73^] |
|  |  |  | GO:0005583 | fibrillar collagen trimer | 2.45 | 8.44E-09 | <0.001 |  |  |
|  |  |  | GO:0003009 | skeletal muscle contraction | 2.12 | 2.50E-09 | <0.001 |  |  |
|  |  |  |  |  |  |  |  |  |  |
| 23 | late erythrocyte (eryth2) | late erythrocyte | GO:0031722 | hemoglobin beta binding | 3.50 | 2.62E-06 | 0.008 | [Snca, Alas2, Car2, Gypa, Ube2l6] | [Bpgm^57^, Hbb-bt^57^, Hbb-bs^57^, Hbb-bh1^57^, Hba-a2^57^, Hba-a1^57^] |
|  |  |  | GO:0031721 | hemoglobin alpha binding | 3.18 | 1.63E-08 | <0.001 |  |  |
|  |  |  | GO:0048821 | erythrocyte development | 2.07 | 6.14E-06 | 0.01 |  |  |
|  |  |  |  |  |  |  |  |  |  |
| 24 | Ihh + chondrocyte (chon^Ihh+)^ | chondrocyte expressing Ihh | GO:0005594 | collagen type IX trimer | 2.90 | 6.82E-07 | 0.003 | [Col2a1, Col11a1, Hapln1, Col9a3, Col9a1] | [Acan^3^, Col2a1^3^, Ihh^3^, Mef2c^3^, Pth1r^74^, Runx2^74^, Sox9^14^] |
|  |  |  | GO:0002063 | chondrocyte development | 1.81 | 2.69E-10 | <0.001 |  |  |
|  |  |  | GO:0060351 | cartilage development involved in endochondral bone morphogenesis | 1.78 | 7.57E-09 | <0.001 |  |  |
|  |  |  | GO:0045667 | regulation of osteoblast differentiation | 1.08 | 2.97E-09 | <0.001 |  |  |

* Two markers of mast cells that are not expressed in macrophages are seen in this cluster.

**Supplementary Tables 1 and 2 Legend:**

Sample sizes for input to FindMarkers before setting min. diff. pct =0.2: mesenchyme, 1242 genes; perichondrium, 3108 genes; chondrocytes, 3756 genes, muscle 3, 2090 genes, muscle 1, 962 genes; neural crest, 2933 genes; EMP, 3463 genes; endothelial, 3145 genes; muscle 2, 2114; macrophage, 3022 genes; epithelial, 2625 genes. Sample sizes for 10x clusters are: Cluster 0, 398 genes; Cluster 1, 248 genes; Cluster 2, 70 genes; Cluster 3, 164 genes; Cluster 4, 146 genes; Cluster 5, 217 genes; Cluster 6, 262 genes; Cluster 7, 265 genes; Cluster 8, 734 genes; Cluster 9, 622 genes; Cluster 10, 160 genes; Cluster 11, 349 genes; Cluster 12, 556 genes; Cluster 13, 156 genes; Cluster 14, 334 genes; Cluster 15, 623 genes; Cluster 16, 542 genes; Cluster 17, 322 genes; Cluster 18, 431 genes; Cluster 19, 397 genes; Cluster 20, 592 genes; Cluster 21, 881 genes; Cluster 22, 117 genes; cluster 23, 42 genes; Cluster 24, 336 genes.

The statistical test for Gene Ontology analysis is a single-hypothesis P-value of the association between attribute and query (based on Fisher’s Exact Test). P-adj: fraction (as a %) of 1000 null-hypothesis simulations having attributes with this single-hypothesis P value or smaller. GO search sample sizes for C1 clusters are: mesenchymal, 90 genes; perichondrial, 369 genes; chondrocyte, 279 genes; muscle 3, 365 genes; muscle 1, 117 genes; neural crest, 165 genes; EMP, 393 genes; endothelial, 314 genes; muscle 2, 236 genes; macrophage, 632 genes; epithelial, 169 genes. GO search sample sizes for 10x clusters are: cluster 0, 177 genes; cluster 1, 116 genes; cluster 2, 16 genes; cluster 3, 30 genes, cluster 4, 100 genes, cluster 5, 158 genes, cluster 6, 173 genes, cluster 7, 153 genes, cluster 8, 679 genes; cluster 9, 561 genes; cluster 10, 50 genes; cluster 11, 170 genes; cluster 12, 516 genes; cluster 13, 149 genes; cluster 14, 305 genes; cluster 15, 2 genes; cluster 16, 450 genes, cluster 17, 263 genes, cluster 18, 380 genes, cluster 19, 323 genes; cluster 20, 520 genes; cluster 21, 833 genes; cluster 22, 60 genes; cluster 23, 38 genes; cluster 24, 205 genes.

**References**

1. Berriz, G. F., Beaver, J. E., Cenik, C., Tasan, M. & Roth, F. P. Next generation software for functional trend analysis. *Bioinformatics* **25**, 3043–3044 (2009).

2. Hsu, S.-H. C. *et al.* Suppressor of fused (Sufu) mediates the effect of parathyroid hormone-like hormone (Pthlh) on chondrocyte differentiation in the growth plate. *J. Biol. Chem.* **287**, 36222–36228 (2012).

3. Kozhemyakina, E., Lassar, A. B. & Zelzer, E. A pathway to bone: signaling molecules and transcription factors involved in chondrocyte development and maturation. *Development* **142**, 817–831 (2015).

4. Shea, K. L. *et al.* Sprouty1 Regulates Reversible Quiescence of a Self-Renewing Adult Muscle Stem Cell Pool during Regeneration. *Cell Stem Cell* **6**, 117–129 (2010).

5. Sato, T., Rocancourt, D., Marques, L., Thorsteinsdóttir, S. & Buckingham, M. A Pax3/Dmrt2/Myf5 Regulatory Cascade Functions at the Onset of Myogenesis. *PLoS Genet.* **6**, e1000897 (2010).

6. Zakany, J. & Duboule, D. The role of Hox genes during vertebrate limb development. *Curr. Opin. Genet. Dev.* **17**, 359–366 (2007).

7. Bensoussan-Trigano, V., Lallemand, Y., Saint Cloment, C. & Robert, B. Msx1 and Msx2 in limb mesenchyme modulate digit number and identity. *Dev. Dyn.* **240**, 1190–1202 (2011).

8. Krawchuk, D. *et al.* Twist1 activity thresholds define multiple functions in limb development. *Dev. Biol.* **347**, 133–146 (2010).

9. Loebel, D. A. F. *et al.* Regionalized Twist1 activity in the forelimb bud drives the morphogenesis of the proximal and preaxial skeleton. *Dev. Biol.* **362**, 132–140 (2012).

10. Maes, C. *et al.* Osteoblast Precursors, but Not Mature Osteoblasts, Move into Developing and Fractured Bones along with Invading Blood Vessels. *Dev. Cell* **19**, 329–344 (2010).

11. Guntur, A. R., Rosen, C. J. & Naski, M. C. N-cadherin adherens junctions mediate osteogenesis through PI3K signaling. *Bone* **50**, 54–62 (2012).

12. Bandyopadhyay, A., Kubilus, J. K., Crochiere, M. L., Linsenmayer, T. F. & Tabin, C. J. Identification of unique molecular subdomains in the perichondrium and periosteum and their role in regulating gene expression in the underlying chondrocytes. *Dev. Biol.* **321**, 162–174 (2008).

13. Hojo, H., Ohba, S., He, X., Lai, L. P. & McMahon, A. P. Sp7/Osterix Is Restricted to Bone-Forming Vertebrates where It Acts as a Dlx Co-factor in Osteoblast Specification. *Dev. Cell* **37**, 238–253 (2016).

14. Hartmann, C. Transcriptional networks controlling skeletal development. *Curr. Opin. Genet. Dev.* **19**, 437–443 (2009).

15. Terry, E. E. *et al.* Transcriptional profiling reveals extraordinary diversity among skeletal muscle tissues. *eLife* **7**, e34613 (2018).

16. Buckingham, M. & Rigby, P. W. J. Gene Regulatory Networks and Transcriptional Mechanisms that Control Myogenesis. *Dev. Cell* **28**, 225–238 (2014).

17. Bladt, F., Riethmacher, D., Isenmann, S., Aguzzi, A. & Birchmeier, C. Essential role for the c- met receptor in the migration of myogenic precursor cells into the limb bud. *Nature* **376**, 768 (1995).

18. Goulding, M., Lumsden, A. & Paquette, A. J. Regulation of Pax-3 expression in the dermomyotome and its role in muscle development. *Dev. Camb. Engl.* **120**, 957–971 (1994).

19. Williams, B. A. & Ordahl, C. P. Pax-3 expression in segmental mesoderm marks early stages in myogenic cell specification. *Dev. Camb. Engl.* **120**, 785–796 (1994).

20. Bober, E., Franz, T., Arnold, H. H., Gruss, P. & Tremblay, P. Pax-3 is required for the development of limb muscles: a possible role for the migration of dermomyotomal muscle progenitor cells. *Dev. Camb. Engl.* **120**, 603–612 (1994).

21. Ishii, M. *et al.* A Stable Cranial Neural Crest Cell Line from Mouse. *Stem Cells Dev.* **21**, 3069–3080 (2012).

22. Simoes-Costa, M. & Bronner, M. E. Establishing neural crest identity: a gene regulatory recipe. *Development* **142**, 242–257 (2015).

23. Mass, E. *et al.* Specification of tissue-resident macrophages during organogenesis. *Science* **353**, aaf4238–aaf4238 (2016).

24. McKinney-Freeman, S. *et al.* The transcriptional landscape of hematopoietic stem cell ontogeny. *Cell Stem Cell* **11**, 701–714 (2012).

25. Devic, E., Rizzoti, K., Bodin, S., Knibiehler, B. & Audigier, Y. Amino acid sequence and embryonic expression of msr/apj, the mouse homolog of Xenopus X-msr and human APJ. *Mech. Dev.* **84**, 199–203 (1999).

26. Kaipainen, A. The related FLT4, FLT1, and KDR receptor tyrosine kinases show distinct expression patterns in human fetal endothelial cells. *J. Exp. Med.* **178**, 2077–2088 (1993).

27. Jeong, H.-W. *et al.* Transcriptional regulation of endothelial cell behavior during sprouting angiogenesis. *Nat. Commun.* **8**, (2017).

28. Atit, R. *et al.* Beta-catenin activation is necessary and sufficient to specify the dorsal dermal fate in the mouse. *Dev. Biol.* **296**, 164–176 (2006).

29. Gao, X. *et al.* HES6 acts as a transcriptional repressor in myoblasts and can induce the myogenic differentiation program. *J. Cell Biol.* **154**, 1161–1172 (2001).

30. Gagan, J., Dey, B. K., Layer, R., Yan, Z. & Dutta, A. Notch3 and Mef2c proteins are mutually antagonistic via Mkp1 protein and miR-1/206 microRNAs in differentiating myoblasts. *J. Biol. Chem.* **287**, 40360–40370 (2012).

31. Buckingham, M. & Relaix, F. PAX3 and PAX7 as upstream regulators of myogenesis. *Semin. Cell Dev. Biol.* **44**, 115–125 (2015).

32. Schmidt, K., Glaser, G., Wernig, A., Wegner, M. & Rosorius, O. Sox8 Is a Specific Marker for Muscle Satellite Cells and Inhibits Myogenesis. *J. Biol. Chem.* **278**, 29769–29775 (2003).

33. Maeda, T., Chapman, D. L. & Stewart, A. F. R. Mammalian Vestigial-like 2, a Cofactor of TEF-1 and MEF2 Transcription Factors That Promotes Skeletal Muscle Differentiation. *J. Biol. Chem.* **277**, 48889–48898 (2002).

34. Mielcarek, M., Piotrowska, I., Schneider, A., Günther, S. & Braun, T. VITO-2, a new SID domain protein, is expressed in the myogenic lineage during early mouse embryonic development. *Gene Expr. Patterns GEP* **9**, 129–137 (2009).

35. Figeac, N. *et al.* Vgll3 operates via Tead1, Tead3 and Tead4 to influence myogenesis in skeletal muscle. *J. Cell Sci.* jcs.225946 (2019). doi:10.1242/jcs.225946

36. Li, Z., Chen, M. J., Stacy, T. & Speck, N. A. Runx1 function in hematopoiesis is required in cells that express Tek. *Blood* **107**, 106–110 (2006).

37. Summerhurst, K., Stark, M., Sharpe, J., Davidson, D. & Murphy, P. 3D representation of Wnt and Frizzled gene expression patterns in the mouse embryo at embryonic day 11.5 (Ts19). *Gene Expr. Patterns* **8**, 331–348 (2008).

38. Auden, A. *et al.* Spatial and temporal expression of the Grainyhead-like transcription factor family during murine development. *Gene Expr. Patterns GEP* **6**, 964–970 (2006).

39. Bazzi, H., Fantauzzo, K. A., Richardson, G. D., Jahoda, C. A. B. & Christiano, A. M. Transcriptional profiling of developing mouse epidermis reveals novel patterns of coordinated gene expression. *Dev. Dyn.* **236**, 961–970 (2007).

40. Yu, L. *et al.* Shox2 is required for chondrocyte proliferation and maturation in proximal limb skeleton. *Dev. Biol.* **306**, 549–559 (2007).

41. Imamura, Y., Scott, I. C. & Greenspan, D. S. The pro-alpha3(V) collagen chain. Complete primary structure, expression domains in adult and developing tissues, and comparison to the structures and expression domains of the other types V and XI procollagen chains. *J. Biol. Chem.* **275**, 8749–8759 (2000).

42. Späth, S., Andrade, A. C., Chau, M., Baroncelli, M. & Nilsson, O. Evidence That Rat Chondrocytes Can Differentiate Into Perichondrial Cells. *JBMR Plus* **2**, 351–361 (2018).

43. García-Andrés, C. & Torres, M. Comparative expression pattern analysis of the highly conserved chemokines SDF1 and CXCL14 during amniote embryonic development. *Dev. Dyn.* **239**, 2769–2777 (2010).

44. Kimmel, R. A. *et al.* Two lineage boundaries coordinate vertebrate apical ectodermal ridge formation. *Genes Dev.* **14**, 1377–1389 (2000).

45. McDonald, L. A., Gerrelli, D., Fok, Y., Hurst, L. D. & Tickle, C. Comparison of Iroquois gene expression in limbs/fins of vertebrate embryos: Iroquois gene expression. *J. Anat.* **216**, 683–691 (2010).

46. Singh, M. K. *et al.* The T-box transcription factor Tbx15 is required for skeletal development. *Mech. Dev.* **122**, 131–144 (2005).

47. Li, L., Cserjesi, P. & Olson, E. N. Dermo-1: A Novel Twist-Related bHLH Protein Expressed in the Developing Dermis. *Dev. Biol.* **172**, 280–292 (1995).

48. Gerety, S. S. & Anderson, D. J. Cardiovascular ephrinB2 function is essential for embryonic angiogenesis. *Dev. Camb. Engl.* **129**, 1397–1410 (2002).

49. Wei, G. *et al.* Ets1 and Ets2 are required for endothelial cell survival during embryonic angiogenesis. *Blood* **114**, 1123–1130 (2009).

50. Feenstra, J. M. *et al.* Detection of genes regulated by Lmx1b during limb dorsalization: Lmx1b regulated genes in limb dorsalization. *Dev. Growth Differ.* **54**, 451–462 (2012).

51. Zhao, H. *et al.* Foxp1/2/4 regulate endochondral ossification as a suppresser complex. *Dev. Biol.* **398**, 242–254 (2015).

52. Gu, W. X. W. & Kania, A. Identification of genes controlled by LMX1B in E13.5 mouse limbs. *Dev. Dyn.* **239**, 2246–2255 (2010).

53. Wenstrup, R. J. *et al.* Regulation of Collagen Fibril Nucleation and Initial Fibril Assembly Involves Coordinate Interactions with Collagens V and XI in Developing Tendon. *J. Biol. Chem.* **286**, 20455–20465 (2011).

54. Ito, Y. *et al.* The Mohawk homeobox gene is a critical regulator of tendon differentiation. *Proc. Natl. Acad. Sci.* **107**, 10538–10542 (2010).

55. Yin, Z. *et al.* Single-cell analysis reveals a nestin ^+^ tendon stem/progenitor cell population with strong tenogenic potentiality. *Sci. Adv.* **2**, e1600874 (2016).

56. Grosch, M., Fuchs, J., Bösl, M., Winterpacht, A. & Tagariello, A. Selenoprotein M is expressed during bone development. *EXCLI J.* **12**, 967–979 (2013).

57. Choi, J. *et al.* Haemopedia RNA-seq: a database of gene expression during haematopoiesis in mice and humans. *Nucleic Acids Res.* **47**, D780–D785 (2019).

58. Ferronha, T. *et al.* LMO4 is an Essential Cofactor in the Snail2-Mediated Epithelial-to-Mesenchymal Transition of Neuroblastoma and Neural Crest Cells. *J. Neurosci.* **33**, 2773–2783 (2013).

59. Nishino, J. *et al.* Meteorin: a secreted protein that regulates glial cell differentiation and promotes axonal extension. *EMBO J.* **23**, 1998–2008 (2004).

60. Choi, J., Djebbar, S., Fournier, A. & Labrie, C. The co-chaperone DNAJC12 binds to Hsc70 and is upregulated by endoplasmic reticulum stress. *Cell Stress Chaperones* **19**, 439–446 (2014).

61. Richter, K., Haslbeck, M. & Buchner, J. The Heat Shock Response: Life on the Verge of Death. *Mol. Cell* **40**, 253–266 (2010).

62. Wang, C.-H., Wu, S.-B., Wu, Y.-T. & Wei, Y.-H. Oxidative stress response elicited by mitochondrial dysfunction: Implication in the pathophysiology of aging. *Exp. Biol. Med.* **238**, 450–460 (2013).

63. Stephens, A. S. *et al.* Myocyte Enhancer Factor 2C, an Osteoblast Transcription Factor Identified by Dimethyl Sulfoxide (DMSO)-enhanced Mineralization. *J. Biol. Chem.* **286**, 30071–30086 (2011).

64. Qiu, T. *et al.* PTH receptor signaling in osteoblasts regulates endochondral vascularization in maintenance of postnatal growth plate. *J. Bone Miner. Res. Off. J. Am. Soc. Bone Miner. Res.* **30**, 309–317 (2015).

65. Schuster-Gossler, K., Cordes, R. & Gossler, A. Premature myogenic differentiation and depletion of progenitor cells cause severe muscle hypotrophy in Delta1 mutants. *Proc. Natl. Acad. Sci.* **104**, 537–542 (2007).

66. Yokoyama, S. *et al.* A Systems Approach Reveals that the Myogenesis Genome Network Is Regulated by the Transcriptional Repressor RP58. *Dev. Cell* **17**, 836–848 (2009).

67. Majesky, M. W., Dong, X. R., Regan, J. N. & Hoglund, V. J. Vascular Smooth Muscle Progenitor Cells: Building and Repairing Blood Vessels. *Circ. Res.* **108**, 365–377 (2011).

68. Breland, A. *et al.* Smooth Muscle Transcriptome Browser: offering genome-wide references and expression profiles of transcripts expressed in intestinal SMC, ICC, and PDGFRα+ cells. *Sci. Rep.* **9**, (2019).

69. Williams, H. C. *et al.* Role of Coronin 1B in PDGF-Induced Migration of Vascular Smooth Muscle Cells. *Circ. Res.* **111**, 56–65 (2012).

70. McGrath, K. E., Fegan, K. H., Frame, J. M., Kingsley, P. D. & Palis, J. Definitive Erythro-Myeloid Progenitors (EMP) Emerge in the Yolk Sac From Hemogenic Endothelium and Share Transcriptional Regulators with Adult Hematopoiesis. *Blood* **118**, 910–910 (2011).

71. Bianchi, E., Norfo, R., Pennucci, V., Zini, R. & Manfredini, R. Genomic landscape of megakaryopoiesis and platelet function defects. *Blood* **127**, 1249–1259 (2016).

72. Wang, H. *et al.* MEIS1 Regulates Hemogenic Endothelial Generation, Megakaryopoiesis, and Thrombopoiesis in Human Pluripotent Stem Cells by Targeting TAL1 and FLI1. *Stem Cell Rep.* **10**, 447–460 (2018).

73. Vallecillo-García, P. *et al.* Odd skipped-related 1 identifies a population of embryonic fibro-adipogenic progenitors regulating myogenesis during limb development. *Nat. Commun.* **8**, (2017).

74. Amano, K., Densmore, M., Nishimura, R. & Lanske, B. Indian Hedgehog Signaling Regulates Transcription and Expression of Collagen Type X via Runx2/Smads Interactions. *J. Biol. Chem.* **289**, 24898–24910 (2014).

**Supplementary Note 3:**

**Cell-type transcription factor networks.** Transcription factors among the marker genes for multiple cell types were used to build StringDB interaction network graphs (see Methods). Both database (teal) and experimental (magenta) evidence codes were used to produce edges. Node color intensities reflect the abundance of RNA-Seq expression measurements in the 10x data, and the colors correspond to the 10x cluster code of Fig. 3A and their corresponding cell identity associations (Supplementary note 2 Table 2). The color intensity reflects RNA expression level. Each graph node is labeled with its transcription factor gene name.


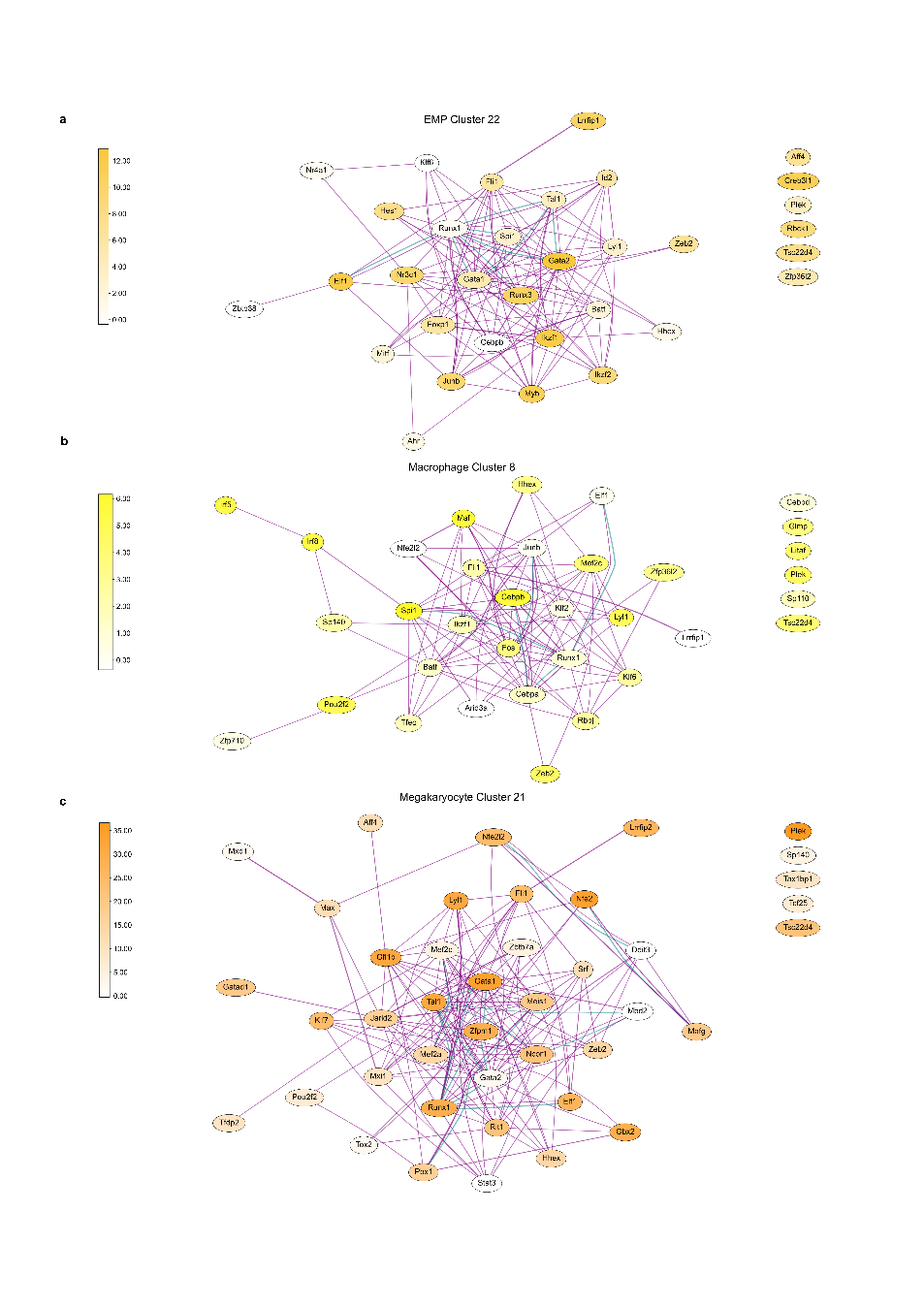


**Supplementary Figure 4: myeloid cell types**. Transcription factors among the marker genes of (**a**) EMP, (**b**) macrophage or (**c**) megakaryocytes were used (see Methods) for interaction analysis by StringDB, and the results were organized into graphs.


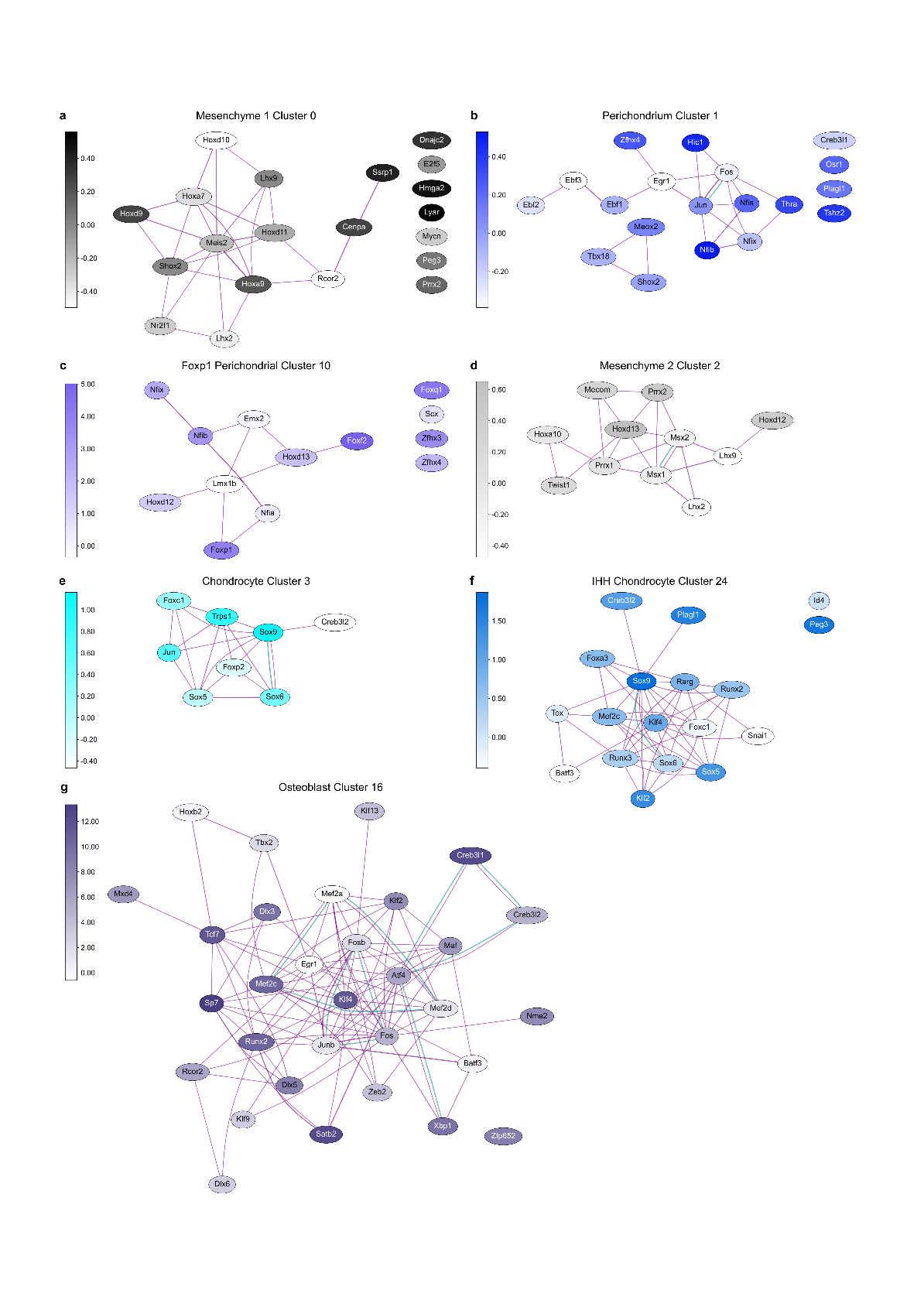


**Supplementary Figure 5: cells of the limb skeleton**. Transcription factors among the marker genes of (**a**) mesenchyme 1, **(b**) perichondrium, (**c**) FoxP1(+) perichondrium, (**d**) mesenchyme 2, (**e**) chondrocyte, (**f**) IHH(+) chondrocyte, or (**g**) osteocyte cells were used (see Methods) for interaction analysis by StringDB, and the results were organized into graphs.


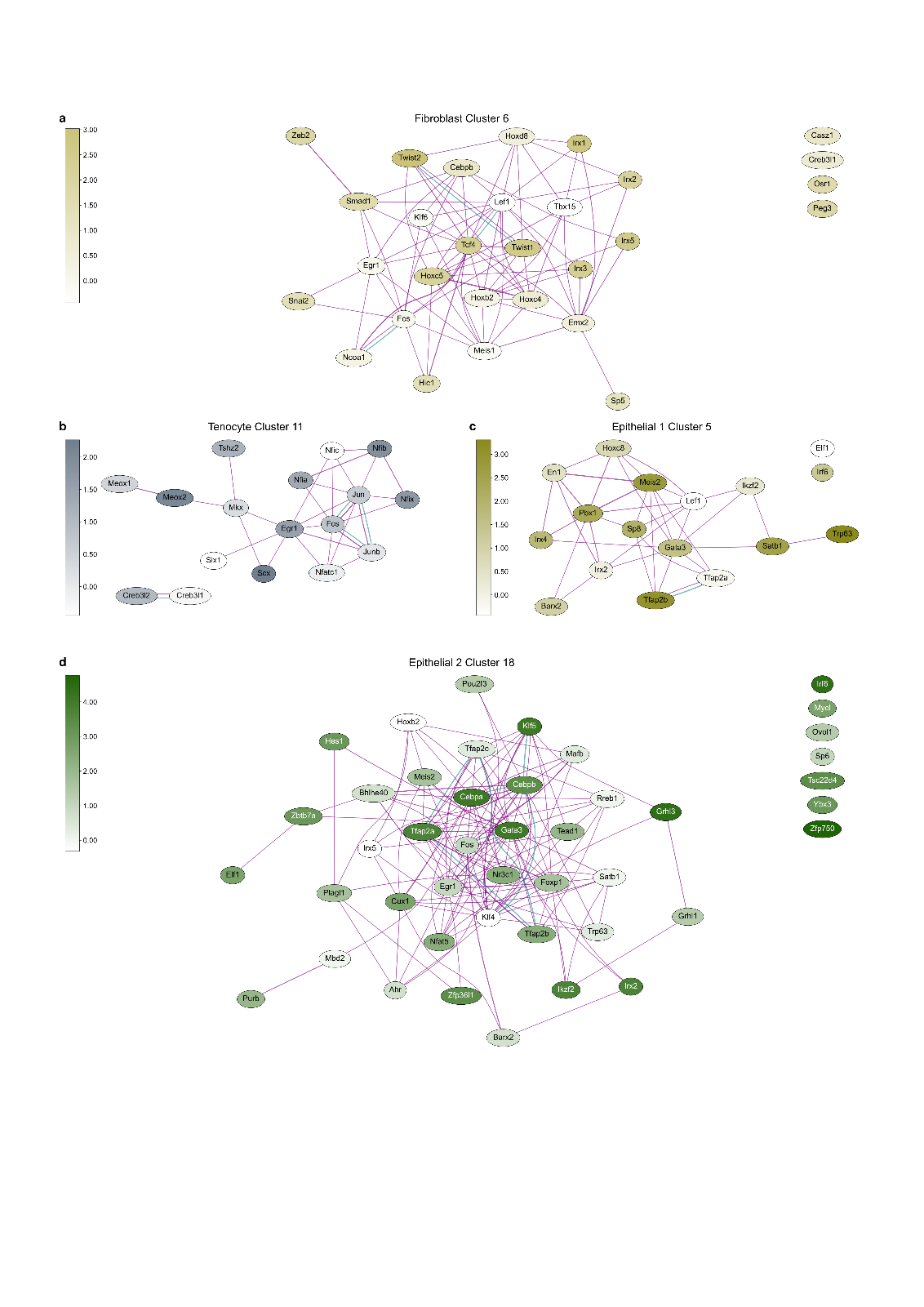


**Supplementary Figure 6:** **connective and epithelial cells**. Transcription factors among the marker genes of (**a)** fibroblasts, (**b**) tenocytes, (**c**) epithelial 1 and (**d**) epithelial 2 cells were used (see Methods) for interaction analysis by StringDB, and the results were organized into graphs.


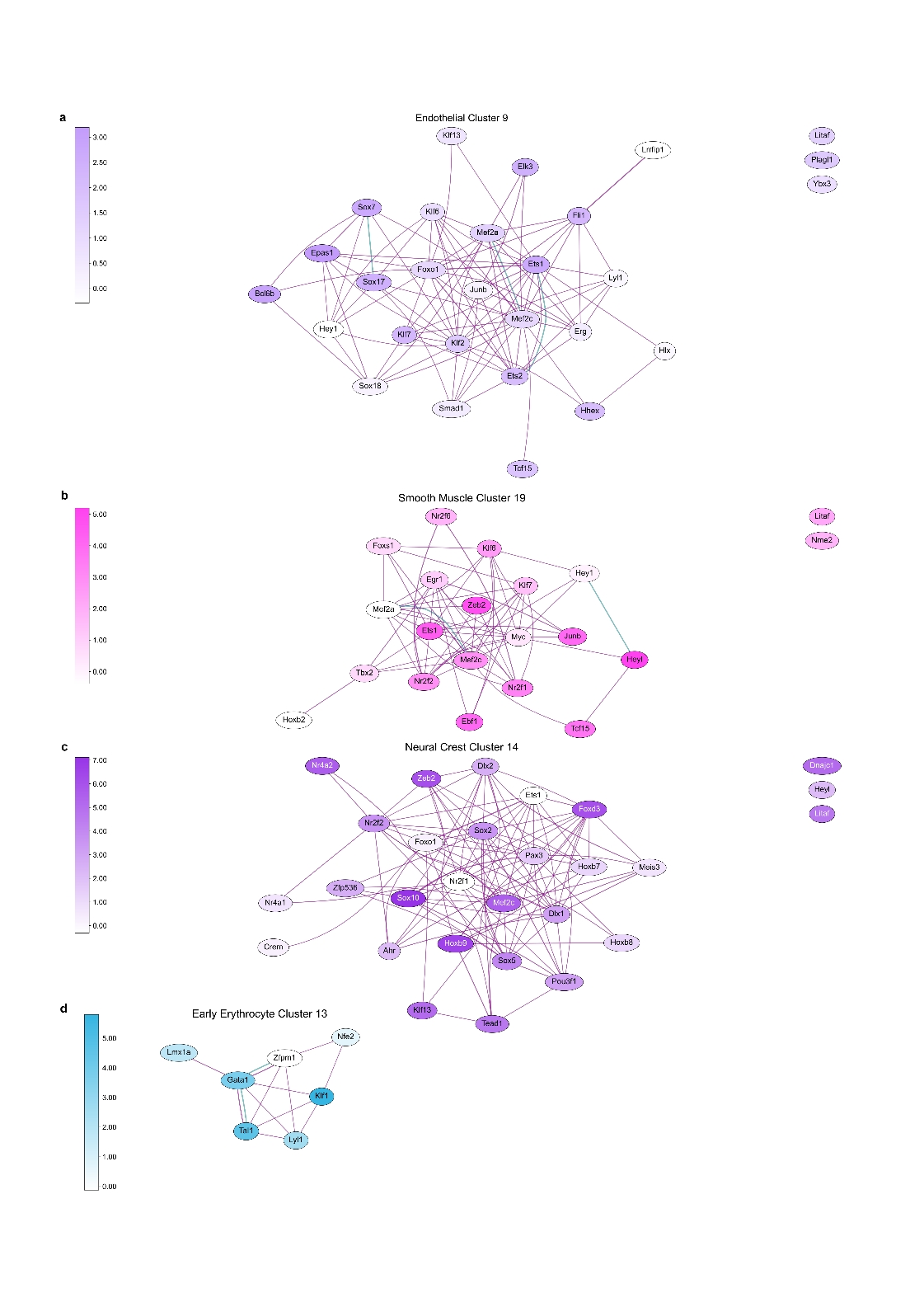


**Supplementary Figure 7:** **vascular and neural crest cells**. Transcription factors among the marker genes of (**a**) endothelial, (**b**) smooth muscle, (**c**) neural crest and (**d**) early erythrocyte cells were used (see Methods) for interaction analysis by StringDB, and the results were organized into graphs.
